# Foamy microglia link oxylipins to disease progression in multiple sclerosis

**DOI:** 10.1038/s41593-026-02302-3

**Published:** 2026-05-21

**Authors:** Daan van der Vliet, Xinyu Di, Tatiana M. Shamorkina, Claire Coulon-Bainier, Anto Pavlovic, Iris A. C. M. van der Vliet, Yingyu Zeng, Will Macnair, Noëlle van Egmond, J. Q. Alida Chen, Aletta M. R. van den Bosch, Hendrik J. Engelenburg, Dennis Wever, Matthew R. J. Mason, Wouter P. F. Driever, Berend Gagestein, Elise Dusseldorp, Marco van Eijk, Uwe Grether, The Netherlands Brain Bank, J. Q. Alida Chen, Aletta M. R. van den Bosch, Mignon de Goeij, Annemieke J. M. Rozemuller, Inge Huitinga, Amy C. Harms, Thomas Hankemeier, Ludovic Collin, Albert J. R. Heck, Inge Huitinga, Mario van der Stelt

**Affiliations:** 1 Department of Molecular Physiology, Leiden Institute of Chemistry, Leiden University, Leiden, the Netherlands; 2 Neuroimmunology Research Group, Netherlands Institute for Neuroscience, Amsterdam, the Netherlands; 3 Biomolecular Mass Spectrometry and Proteomics, Bijvoet Center for Biomolecular Research and Utrecht Institute for Pharmaceutical Sciences, University of Utrecht, Utrecht, the Netherlands; 4 Netherlands Proteomics Center, Utrecht, the Netherlands; 5 Pharma Research and Early Development, Roche Innovation Center Basel, F. Hoffmann-La Roche, Basel, Switzerland; 6 Methodology and Statistics Unit, Institute of Psychology, Leiden University, Leiden, the Netherlands; 7 Department of Bio-organic Synthesis, Leiden Institute of Chemistry, Leiden University, Leiden, the Netherlands; 8 Metabolomics and Analytics Centre, Leiden Academic Centre for Drug Research, Leiden University, Leiden, the Netherlands; 9 Center for Neuroscience, Swammerdam Institute for Life Sciences, Faculty of Science, University of Amsterdam, Amsterdam, the Netherlands; 10 The Netherlands Brain Bank, Netherlands Institute for Neuroscience, Amsterdam, the Netherlands; 11 Department of Pathology, Amsterdam University Medical Centre, Amsterdam, the Netherlands

**Keywords:** Multiple sclerosis, Lipidomics, Proteomic analysis, Neuroimmunology, Microglial cells

## Abstract

Multiple sclerosis (MS) is a chronic neuroinflammatory disease in which demyelinating white matter lesions accumulate and expand, driving irreversible disability. Here we identify a distinct population of foamy GPNMB^+^ microglia/macrophages associated with lesion expansion in secondary progressive MS. Using integrated lipidomic, transcriptomic, proteomic, chemical proteomic and histological analyses of human postmortem MS lesions, we show that lesions containing foamy microglia/macrophages exhibit disrupted lipid metabolism, lysosomal stress and markers associated with heightened phagocytosis and antigen presentation without classical pro-inflammatory signatures. These lesions are enriched for oxylipins, bismonoacylglycerolphosphates and cholesterol esters, and are associated with increased B cell infiltration and IgG1. Monoacylglycerol lipase (MAGL), a lipid-metabolizing enzyme enriched in lesions with foamy microglia/macrophages, emerged as a potential therapeutic target. Inhibition of MAGL promoted lesion recovery and reduced microgliosis in a mouse model of demyelination. Finally, oxylipins in cerebrospinal fluid correlate with the proportion of foamy lesions, suggesting potential biomarkers for progression. Our findings implicate disturbed lipid metabolism in chronic MS pathology and suggest that foamy microglia/macrophages are an interesting cell type to target for progressive disease.

## Abstract (web summary)

Foamy microglia are associated with multiple sclerosis progression, linking phagocytosis, altered lipid metabolism (oxylipins) and lysosomal stress to nonclassical neuroinflammation and disease severity, highlighting potential therapeutic targets.

## Main

Multiple sclerosis (MS) is a complex and debilitating neurological disorder characterized by the formation of inflammatory demyelinating lesions throughout the central nervous system (CNS). Unresolved lesions are associated with extensive axonal damage, gliosis and cell death that result in severe, often irreversible, disability in patients^1,2^. Despite important advances in the development of disease-modifying therapies that are effective in the early phases of MS, there remains a critical unmet need for treatments that can halt disease progression^3^.

**Fig. 1:**
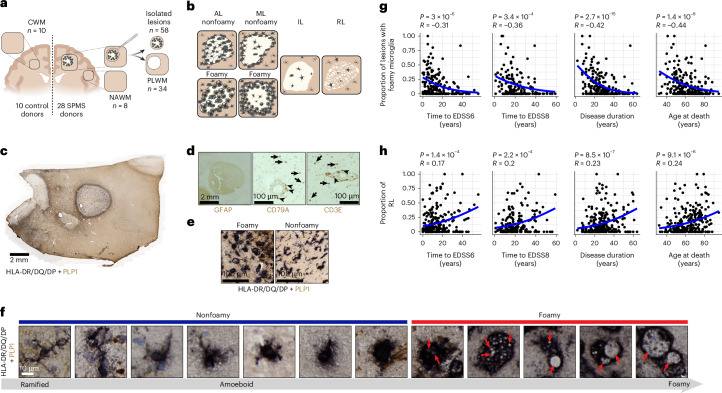
Foamy microglia associate with faster disease progression. **a**, Tissue sampling in this study. SPMS, secondary progressive MS. **b**, Graphical representation of WM lesions. ALs and MLs are subcategorized based on the foamy phenotype. **c**,**d**, Histological analysis of a representative WM lesion, stained for PLP1 (myelin) and HLA-DR/DQ/DP (microglia/macrophages) (**c**), GFAP (astrocytes), CD79A (B cells) or CD3 (T cells) (**d**). All lesions included in this study were histologically analyzed using these stainings (*n* = 58). Arrows indicate parenchymal cells, while arrowheads indicate perivascular cells. **e**, Representative images of a lesion classified as foamy or as nonfoamy. **f**, The continuum of microglial morphologies in MS lesions. Representative images were obtained from stainings for HLA-DR/DQ/DP and PLP1 in **c**. Lipid-filled vacuoles of varying sizes are indicated with red arrows. The rounded morphology in combination with vacuoles is necessary to categorize the microglia as foamy. **g**,**h**, Proportion of lesions with foamy microglia (**g**) or RL (**h**) associates with disease course parameters (*n* = 250 patients with MS). Associations were estimated using quasibinomial generalized linear modeling with a likelihood ratio test and Bonferroni multiple testing correction. Panels created in BioRender: **a**, Van der Vliet, D. https://biorender.com/vb1yn2k (2026); **b**, Van der Vliet, D. https://biorender.com/72njpi8 (2026).

The progression of MS is notably heterogeneous, both in clinical presentation and in the underlying pathology observed in postmortem studies^4^. This heterogeneity is reflected in the diverse pathology of MS lesions, which vary in size, cellular composition, immune cell activity and the extent of demyelination^5–7^. These observations have led to the classification of MS lesions into the following four distinct subtypes: active lesion (AL), mixed active/inactive lesion (ML), inactive lesion (IL) and remyelinated lesion (RL)^1^ (Fig. 1b). Active and mixed active/inactive lesions (AL and ML, respectively) are marked by active microglia and infiltrating macrophages expressing high levels of HLA-DR/DQ/DP, suggesting ongoing demyelination, whereas ILs are characterized by severe astrogliosis and a lack of remyelination, indicating a failure of repair processes and permanent axonal damage. RLs, often referred to as ‘shadow plaques’, exhibit partial repair, which is thought to contribute to the relapsing-remitting nature of early-stage MS. However, capacity for remyelination diminishes with age, often failing in the progressive stages of the disease, leading to chronic loss of neurological function^3,8–10^. A key factor associated with disease progression is the presence of iron rim lesions^11–13^. These lesions are characterized by a persistent rim of iron-laden microglia/macrophages and are strongly associated with ongoing tissue damage and lesion expansion. However, the molecular mechanisms driving lesion expansion, while other lesions resolve, remain elusive.

A particularly intriguing aspect of MS pathology is the presence of foamy microglia within ALs and MLs. These microglia/macrophages, characterized by their lipid-laden appearance^6,14–18^, are associated with increased axonal damage, higher levels of neurofilament light chain in the cerebrospinal fluid (CSF), and greater overall lesion load, indicating a detrimental effect of foamy microglia/macrophages in MS lesions^5,6,19^. However, foamy microglia/macrophages have also been described as anti-inflammatory or immunosuppressed^14,18^. The exact role of foamy microglia in lesion etiology is currently still unclear.

Here we have addressed these gaps by conducting a comprehensive multi-omics analysis, integrating lipidomics, transcriptomics and proteomics to systematically profile well-characterized postmortem white matter (WM) lesions from patients with MS. Our findings highlight an important role for foamy microglia/macrophages in determining the molecular profile of MS lesions. Lesions with foamy microglia/macrophages were characterized by cholesterol-ester and bismonoacylglycerolphosphate (BMP) accumulation, and the production of oxidized free fatty acids (oxylipins). In contrast, lesions with ramified microglia are more closely associated with successful remyelination. We identified possible drug targets to address the foamy phenotype using activity-based protein profiling. Finally, we show that oxylipins in CSF associate with the foamy phenotype. Our findings suggest that modulating lipid metabolism in foamy microglia/macrophages may offer a promising therapeutic strategy to modulate disease progression in MS.

## Results

### Lesions with foamy microglia associate with faster MS disease progression

**Extended Data Fig. 1:**
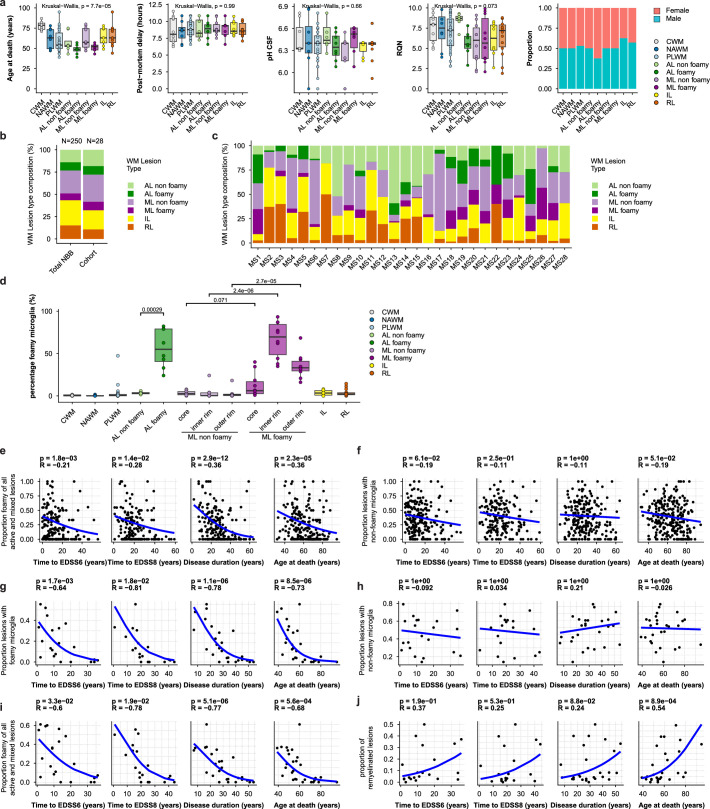
Clinical characteristics of the selected cohort. **a**, Baseline characteristics of the donors. RQN indicates the RNA quality number from the RNA isolated from the isolated lesions. Data are expressed as individual values (circles), the median as the center, boxes representing the first and third quartiles and whiskers extending to datapoints within the 1.5× interquartile range. Number of replicates are specified in Table 1. P-values were calculated by a Kruskal–Wallis test. **b**, Distribution of lesion types across all lesions in the donors from the entire Netherlands Brain Bank (N = 250) or the cohort analyzed here (N = 28). **c**, Distribution of lesion types within individual donors shows variability of lesion types between individuals. **d**, Quantification of the number of microglia classified as foamy with lesions. MLs were analyzed, separating the areas of the lesion: core, inner rim, outer rim. The demyelination border separates the inner rim from the outer rim. Data are expressed as individual values (circles), the median as the center, boxes representing the first and third quartiles and whiskers extending to datapoints within the 1.5× interquartile range. P-values represent two-sided Wilcoxon rank-sum tests without correction for multiple testing. **e**–**j**, Association of lesion type proportion with disease progression parameters for the entire NBB cohort (**e**,**f**) or the cohort analyzed here (**g**–**j**). A generalized linear model was used to model the association using a quasibinomial distribution for lesion type proportion, with a likelihood-ratio test to obtain a p-value, which was corrected for multiple testing by Bonferroni correction. A Spearman correlation (expressed as Spearman’s correlation coefficient R) was performed to assess the variance explained by the association. Source data

The heterogeneity of MS disease progression is reflected by diverse postmortem WM pathology^6,7^. Thus, by studying the molecular profile of diverse WM lesions across a diverse MS cohort, we hypothesized to uncover molecular patterns related to disease progression. To this end, we isolated fresh-frozen subcortical WM samples from 28 individuals diagnosed with secondary progressive MS with diverse WM pathology (Extended Data Fig. 1c) and from 10 controls from the Netherlands Brain Bank (NBB; Fig. 1a–f). A total of 110 samples were isolated and histologically analyzed using staining for PLP1 (myelin) and HLA-DR/DQ/DP (microglia/macrophages), GFAP (astrocytes), CD79A (B cells) or CD3 (T cells). This yielded 58 active, mixed active/inactive, inactive and remyelinated lesions^1,6^ (AL, ML, IL and RL, respectively), alongside 52 samples from normal-appearing white matter (NAWM), peri-lesional white matter (PLWM) and control white matter (CWM; Fig. 1a,b and Table 1). ALs and MLs were further subcategorized based on microglial morphology—either foamy or nonfoamy^6,14–17^ (Fig. 1e and Table 1). Foamy microglia/macrophages were defined as HLA^+^ cells with an amoeboid or rounded morphology containing a vacuolated cytoplasm and as nonfoamy if these vacuoles were not present^6,14,15,17^ (Fig. 1f). In our cohort, lesions classified as foamy contained on average 52% foamy microglia/macrophages of the total pool of microglia/macrophages (range = 24–93%; Extended Data Fig. 1d). They were particularly enriched in the demyelinated areas of ML rims and throughout ALs. In contrast, CWM and NAWM contained on average less than 1% foamy microglia/macrophages, while lesions classified as nonfoamy contained on average 3.2% foamy microglia/macrophages (Extended Data Fig. 1d). Of note, currently there is no staining available to distinguish activated microglia from infiltrating macrophages in human postmortem tissue, but we refer to HLA-DR/DQ/DP-positive cells as microglia in the main text for the ease of readability.

**Table 1:** Overview of replicates for each lesion type in each analysis. Replicates are expressed as lesions/donors

Analysis	CWM	NAWM	PLWM	AL nonfoamy	AL foamy	ML nonfoamy	ML foamy	IL	RL	Total
Cytokines	6/6	8/8	**–**	**–**	**–**	5/5	7/7	**–**	**–**	26/26
Lipidomics	10/10	8/8	32/24	5/4	6/6	9/9	9/7	7/6	14/10	100/37
Proteomics	10/10	8/8	30/23	5/4	7/7	10/9	10/7	6/4	14/10	100/38
RNA-seq	10/10	8/8	34/26	8/7	7/7	10/9	10/7	6/6	12/9	105/38
ABPP	10/10	8/8	31/24	6/5	8/8	10/9	10/7	8/7	14/10	105/37
Total	10/10	8/8	34/26	8/7	8/8	10/9	10/7	8/7	14/10	110/38

To assess the clinical relevance of the subcategorization of ALs and MLs in either foamy or nonfoamy, we coupled pathological data from all patients with MS in the NBB (*n* = 250 MS donors, 8,708 WM lesions analyzed) to the time to reach expanded disability status scales 6 and 8 (EDSS6 and EDSS8), the total disease duration and age at death as disease trajectory parameters. A high proportion of ALs and MLs with foamy microglia in the donor’s WM lesions was associated with faster progression (Fig. 1g and Extended Data Fig. 1e). In contrast, ALs and MLs with nonfoamy microglia did not correlate with progression, while a high proportion of RLs correlated with slower progression (Fig. 1h and Extended Data Fig. 1f). These associations were also observed in this study’s cohort for molecular analyses (*n* = 28; Extended Data Fig. 1g–j).

### Lipidomics reveals a disturbed lipid profile of lesions with foamy microglia

**Fig. 2:**
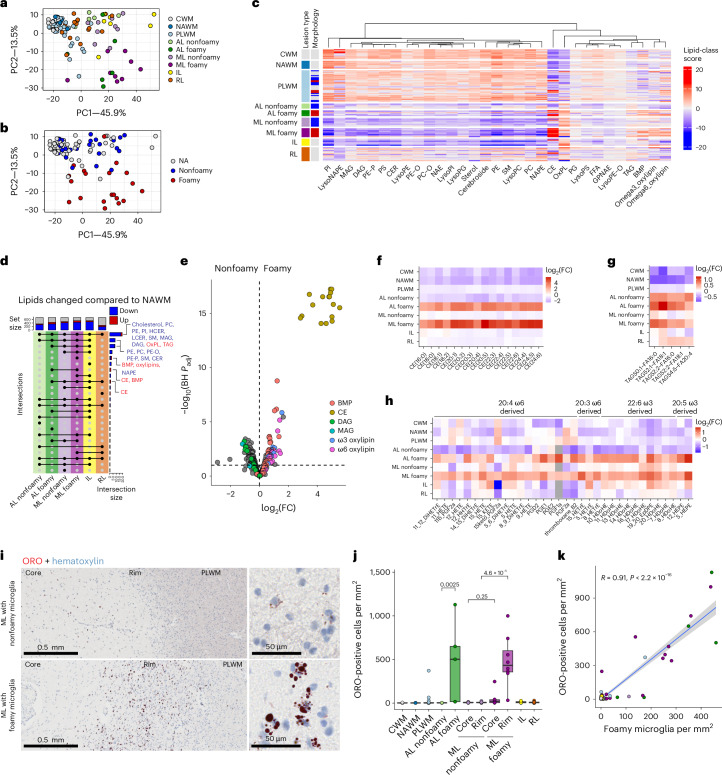
Lipidomics reveals general loss of lipids in lesions and a distinct lipid profile of lesions with foamy microglia. **a**,**b**, PCA of the lipidomics dataset colored by lesion type (**a**) or morphology (**b**). ‘NA’ indicates samples without microglial activation. **c**, Clustered heatmap depicting the first principal component for each lipid class (the ‘eigenlipid’). Hierarchical clustering was performed using Euclidean distances. **d**, UpSet plot depicting the overlap of differentially abundant lipids for each lesion type compared with NAWM. **e**, Differentially abundant lipids between lesions with foamy microglia compared with lesions with nonfoamy microglia. *P* values are calculated using limma (two sided) with BH correction for multiple testing (FDR < 10%). **f**–**h**, Normalized mean abundance of lipid classes CE (**f**), TAG (**g**) or oxylipins (**h**) across lesion types. **i**, Representative image of ORO staining of MLs with foamy and nonfoamy microglia. **j**, Quantification of ORO-positive cells across lesion types. Replicates are expressed as lesions/donors—CWM, *n* = 9/9; NAWM, *n* = 6/6; PLWM, *n* = 31/24; AL nonfoamy, *n* = 7/6; AL foamy, *n* = 5/5; ML nonfoamy, *n* = 10/9; ML foamy, *n* = 8/5; IL, *n* = 8/7; and RL, *n* = 14/6. Statistical testing was two-sided Wilcoxon rank-sum tests on ORO-quantified lesions. Data are expressed as individual values (circles), the median as the center, boxes representing the first and third quartiles and whiskers extending to datapoints within the 1.5× interquartile range. **k**, Correlation (Pearson’s) of ORO-positive cells per mm^2^ and the number of foamy microglia per mm^2^. The error band represents the 95% confidence interval. FC, fold change. Source data

While lipids are central to the biology of myelin^20^ and foamy microglia^18^, this class of biomolecules has been understudied compared to gene expression^11,21–26^ in the context of MS^27^. Therefore, we set out to perform a comprehensive targeted lipidomics analysis of MS lesions. We measured 712 lipid species across 31 lipid classes using targeted liquid chromatography coupled to tandem mass spectrometry (LC–MS/MS; Supplementary Table 1). The analysis revealed that all MS lesions had a distinct lipid composition compared to CWM, NAWM and PLWM, with variability driven by lesion presence/absence and microglial morphology (Fig. 2a–c).

**Extended Data Fig. 2:**
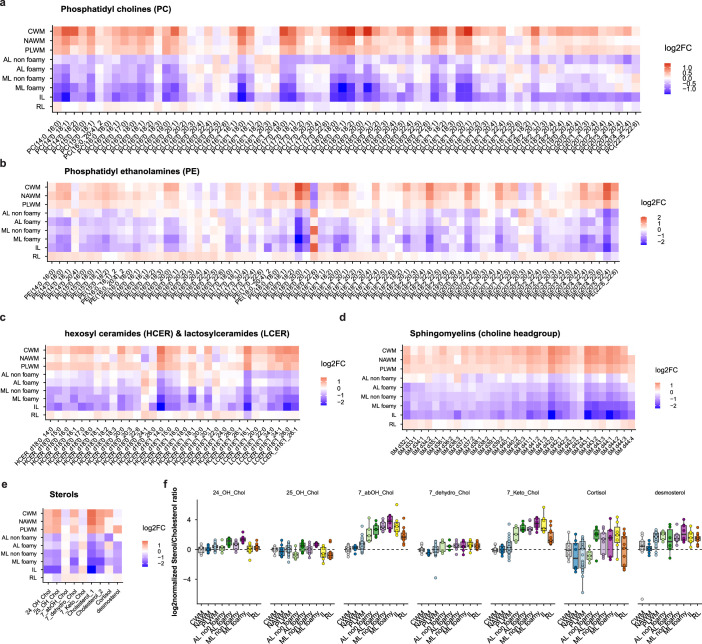
Lipidomics shows loss of lipids in MS lesions. **a**–**e**, Data represent the normalized mean log_2_-transformed levels across lesion types for phosphatidyl choline (PC, **a**), phosphatidyl ethanolamine (PE, **b**), cerebrosides hexosylceramide (HCER) and lactosylceramide (LCER) (**c**), sphingomyelins (**d**) and sterols (**e**). **f**, The change in sterol/cholesterol ratios across lesion types. We lack absolute quantification of these lipids, so we cannot report the absolute ratio, only the relative change in ratio. Data are expressed as individual values (circles), the median as the center, boxes representing the first and third quartiles and whiskers extending to datapoints within the 1.5× interquartile range. Number of replicates are specified in Table 1.

**Extended Data Fig. 3:**
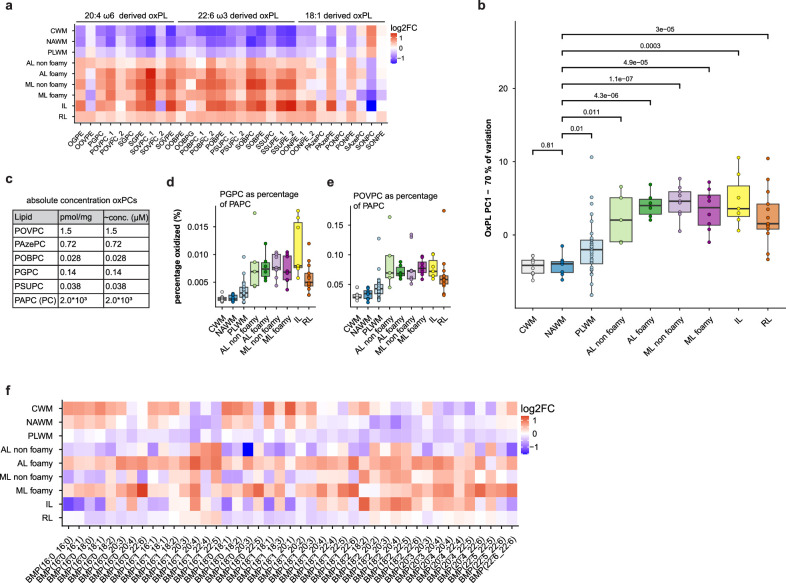
Oxidized phospholipid levels and bismonoacylglycerolphosphates. **a**, Oxidized phospholipid (oxPL) levels across lesions. Data represent the normalized mean log_2_-transformed levels. **b**, The first principal component of the oxPL levels across lesion types, summarizing all oxPL species into one score. P-values were calculated with two-sided Wilcoxon rank-sum tests without correction for multiple testing. **c**, Mean absolute levels of selected oxPL species in MS lesion. The concentration is reported as pmol per mg tissue, which is equivalent to µM assuming a tissue density of 1 g ml^−1^. Palmitoyl arachidonoyl phosphatidyl choline (PAPC), the precursor to palmitoyl 5’-oxo-valeroyl (POVPC) and palmitoyl glutaryl PC (PGPC), is given as reference. **d**,**e**, The percentage of PAPC that is oxidized to PGPC (**d**) or POVPC (**e**). For panels **b**, **d** and **e**, data are expressed as individual values (circles), the median as the center, boxes representing the first and third quartiles and whiskers extending to datapoints within the 1.5× interquartile range. **f**, Bismonoacylglycerolphosphate (BMP) levels across lesions. Data represent the normalized mean log_2_-transformed levels. Number of replicates are specified in Table 1.

Myelin-associated and membrane lipids, such as cholesterol, phospholipids and sphingolipids, were significantly reduced in all lesion types (Fig. 2c,d and Extended Data Fig. 2a–e). These changes were mainly associated with the extent of demyelination, as the decrease of these lipids was much less in RLs (Fig. 2c and Extended Data Fig. 2a–e). The ratio between cholesterol and its derivatives increased (Extended Data Fig. 2f), indicating a shift from cholesterol synthesis to cholesterol recycling and oxidation in lesions^28^. Truncated per-oxidized phospholipids (oxPLs), particularly those derived from poly-unsaturated acyl chains, were elevated in all lesion types as described previously^29,30^. Absolute quantification revealed on average ~1.5 pmol POVPC per mg tissue or lower for other oxPC species. This equates to an estimated concentration less than 1.5 µM (Extended Data Fig. 3a–e), which is 10,000-fold lower than previously used to induce demyelination in mouse models^29,31^.

Lesions with foamy microglia exhibited increased cholesterol esters (CEs) and triacylglycerols (TAG), consistent with their lipid-laden phenotype, which was confirmed by the neutral lipid staining Oil Red O (ORO)^14,16^ (Fig. 2f,g,i–k). Notably, BMPs (Extended Data Fig. 3f) and oxylipins (Fig. 2h) were also strongly increased in lesions with foamy microglia, which may result from enhanced biogenesis and/or impaired clearance, and may suggests increased lysosomal content and an inflammatory state, respectively^32–34^.

**Extended Data Fig. 4:**
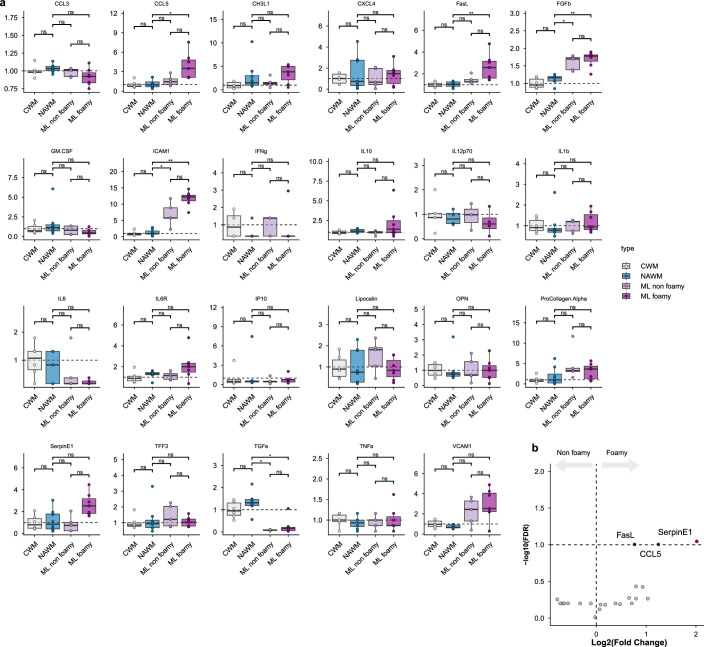
Cytokine profiling in MS lesions. **a**, Box plots of cytokine concentrations in CWM (N = 6 lesions), NAWM (N = 8 lesions) and MLs with either nonfoamy (N = 5 lesions) or foamy microglia (N = 7 lesions). All values were normalized on the CWM mean. Data are expressed as individual values (circles), the median as the center, boxes representing the first and third quartiles and whiskers extending to datapoints within the 1.5× interquartile range. **b**, Volcano plot of cytokine differences between ML with foamy (N = 7) compared ML with nonfoamy microglia (N = 5). All p-values in **a** and **b** were calculated by limma (two-sided) with BH correction for multiple testing with FDR < 10%.

To directly assess the inflammatory status of the lesions, we performed a cytokine screening of ML with either foamy or nonfoamy microglia compared to NAWM and CWM. Notably, prominent inflammatory markers, like TNF, IL6, IFNγ and IL1β, were not different between nonfoamy and foamy lesions (Extended Data Fig. 4). Only three cytokines, associated with apoptosis (FasL)^5,35^, adaptive immune system signaling^36^ (CD8^+^ T cells, CCL5) and cellular senescence (SerpinE1)^37,38^, were significantly upregulated in lesions with foamy microglia compared to nonfoamy.

### Transcriptomics and proteomics capture a distinct profile of lesions with foamy microglia

**Fig. 3:**
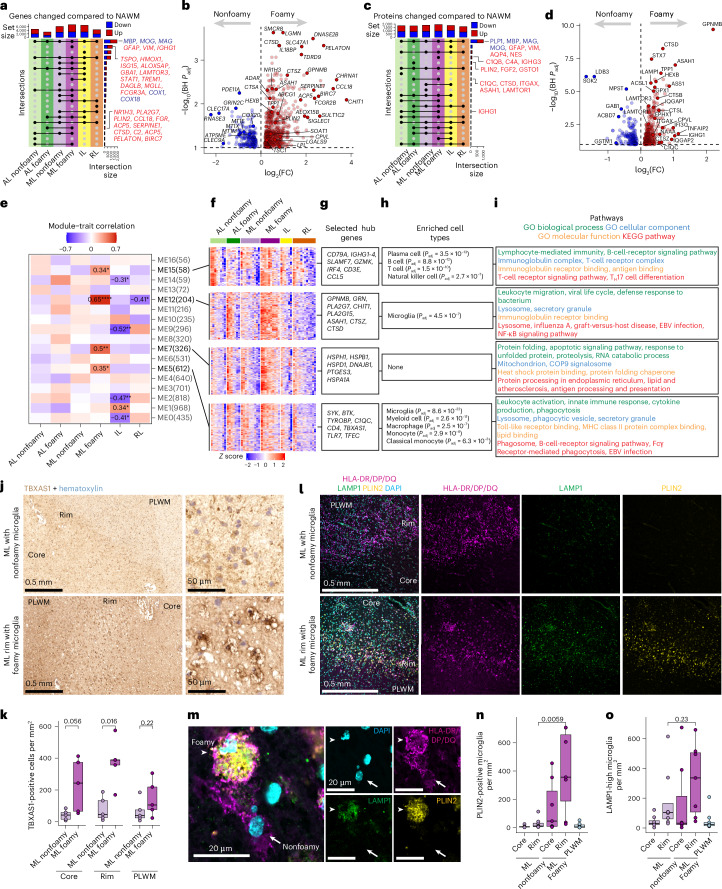
Lesions with foamy microglia have disturbed lipid metabolism and immune regulation. **a**, UpSet plot depicting the overlap of differentially expressed genes for each lesion type compared to NAWM. **b**, Differentially expressed genes between lesions with foamy microglia compared to lesions with nonfoamy microglia. **c**, UpSet plot depicting the overlap of differentially abundant proteins for each lesion type compared to NAWM. **d**, Differentially abundant proteins between lesions with foamy microglia compared to lesions with nonfoamy microglia. All *P* values in **a**–**d** were calculated using limma (two sided) with BH correction for multiple testing (FDR < 10%). WGCNA divided the transcriptome of lesions into 17 modules of coexpressed genes (**e**–**j**). Four gene modules related to MLs with foamy microglia. **e**, Module eigengenes were correlated to lesion types. Correlation was calculated using Pearson correlations adjusted with BH multiple testing correction. *P_adj_ < 0.1,**P_adj_ < 0.01, ****P_adj_ < 0.0001. **f**, Heatmaps depicting *z*-scored counts per million (CPM) values of genes in the selected modules. **g**, Selected hub genes for each module. **h**,**i**, Enriched cell types from brain or blood (**h**) and pathways (**i**) in the selected modules (one-sided Fisher’s exact test for overrepresentation analysis). GO, Gene Ontology; KEGG, Kyoto Encyclopedia of Genes and Genomes; EBV, Epstein–Barr virus; MHC, major histocompatibility complex. **j**, Representative images of TBXAS1 staining of the rim of MLs with foamy or nonfoamy microglia. **k**, Quantification of TBXAS1-positive cells per mm^2^ across PLWM, rim and core of MLs with foamy or nonfoamy microglia (*n* = 5 independent lesions per group). **l**, Representative image of PLIN2 and LAMP1 staining of the rim of MLs with foamy or nonfoamy microglia. **m**, Close-up image of a rim region of an ML containing a foamy (arrowhead) and a nonfoamy (arrow) microglial cell. **n**,**o**, Quantification of PLIN2-positive (**n**) and LAMP1-high (**o**) cells per mm^2^ across the rim and core regions of MLs with foamy or nonfoamy microglia and PLWM. Replicates are expressed as lesions/donors—PLWM, *n* = 9/9; ML nonfoamy, *n* = 8/7; and ML foamy, *n* = 7/4. Data in **k**, **n** and **o** are expressed as individual values (circles), the median as the center, boxes representing the first and third quartiles and whiskers extending to datapoints within the 1.5× interquartile range, and *P* values were calculated with two-sided Wilcoxon rank-sum tests on individual lesions. Source data

To better characterize the biological state of different WM MS lesions, we performed bulk RNA sequencing (RNA-seq) and proteomics to capture both gene expression and post-transcriptional processes (Fig. 3). We obtained expression data for 16,651 genes and 3,237 proteins, which generally showed poor correlation (Supplementary Fig. 1a–c). Notably, structural axonal proteins, whose gene transcripts primarily reside in neuronal somas, did not correlate well with protein levels, while proteins expressed by oligodendrocytes and microglia showed stronger correlation with their gene transcripts (Supplementary Fig. 1d–g). The primary drivers of variance in the transcriptomics and proteomics datasets were lesion presence/absence and the foamy phenotype (Supplementary Fig. 2), similar to the lipidomics data. Most differentially expressed genes and proteins were consistent across all lesion types (Fig. 3a,c), reflecting general lesion features such as demyelination (Supplementary Fig. 3), oligodendrocyte loss and astrogliosis. In addition, lesions with foamy microglia had a distinct transcriptome (Fig. 3b) and proteome (Fig. 3d) from those with nonfoamy microglia. We observed increased expression of genes and proteins involved in cholesterol recycling, lipid droplet formation, oxylipin production and ferroptosis (Supplementary Figs. 4 and 5), aligning well with the lipidomics dataset.

**Extended Data Fig. 5:**
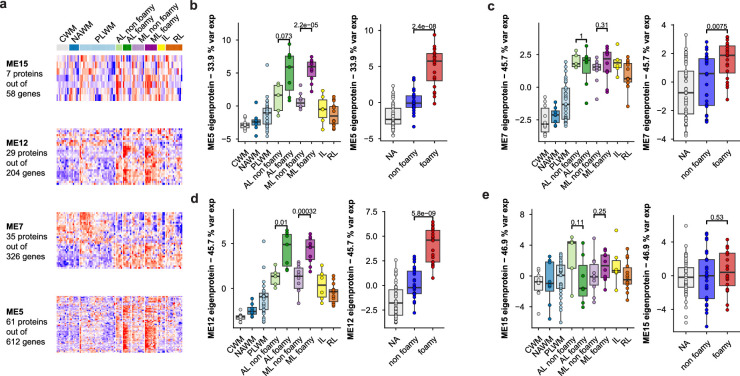
Replication of WGCNA modules on the protein level. **a**, Genes from the four modules associated with ML with foamy (Fig. 3e) microglia were extracted from the WGCNA, and their protein products were searched in the proteomics dataset. A clustered heatmap depicting z-scores of those proteins per module is depicted. **b**–**e**, Analogously to the eigengene method in WGCNA, an ‘eigenprotein’ was calculated for the proteins corresponding to one gene-module and the first principal component is plotted in a box plot, with a two-sided Wilcoxon rank-sum test to assess significance between lesion types or morphology. For panels **b**–**e**, data are expressed as individual values (circles), the median as the center, boxes representing the first and third quartiles and whiskers extending to datapoints within the 1.5× interquartile range. Number of replicates are specified in Table 1.

**Extended Data Fig. 6:**
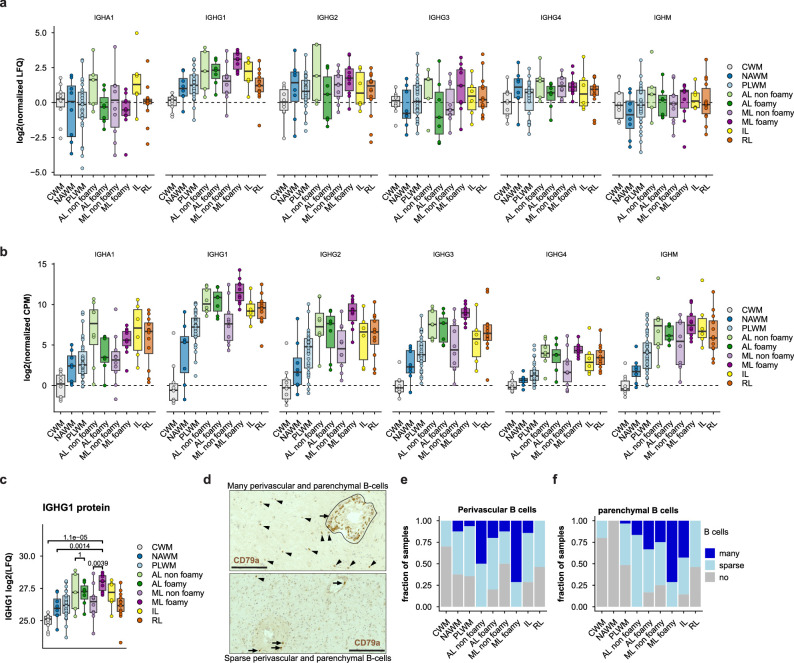
B cells and immunoglobulin expression across lesion types. **a**,**b**, Levels of immunoglobulin constant regions as determined by proteomics (**a**) or RNA sequencing (**b**). **c**, IgG1 (*IGHG1*) is strongly upregulated in lesions compared to CWM and NAWM, especially high in ML with foamy microglia. IGHG1 protein levels are also significantly higher in ML with foamy microglia compared to ML with nonfoamy microglia, this is not the case for active lesions. For panels **a**–**c**, data are expressed as individual values (circles), the median as the center, boxes representing the first and third quartiles and whiskers extending to datapoints within the 1.5× interquartile range. Number of replicates are specified in Table 1. P-values were calculated by a two-sided Wilcoxon rank-sum test. **d**, Representative images of MS lesion with high perivascular and infiltrating B cells (top) and sparse B cells (bottom). Arrowheads indicate parenchymal cells, while arrows indicate perivascular cells. Representative images are shown from 52 stainings on tissue blocks from 38 donors. **e**,**f**, Samples were scored as having many, sparse or no B-cells in the perivascular space (**e**) or infiltrating into the parenchyma (**f**).

To identify the biological networks driving the foamy phenotype in an unbiased manner, we conducted a weighted gene coexpression network analysis (WGCNA)^39^ (Fig. 3e–i and Supplementary Tables 2–4). We identified four modules—ME5, ME7, ME12 and ME15—that were significantly associated with MLs containing foamy microglia (Fig. 3e). The microglia-enriched ME12 module was most strongly linked to the foamy phenotype, while it was significantly downregulated in RLs. This module featured *GPNMB*^40^, *GRN*^41^ and *CHIT1* (ref. ^42^), marker genes typically associated to lysosomal dysfunction, as hub genes and enriched for lysosomal genes (*PLA2G15*, *ACP5*, *ASAH1*, *DNASE2B*, *LGMN*, *CTSZ*, *CTSD*), antiviral response genes (*OAS1-3*, *OASL*, *ISG15*, *HLA-A*, *HLA-B*, *HLA-DQ*, *TRIM25*, *MX1*, *MX2*) and antigen presentation genes (*HLA-A*, *HLA-B*, *HLA-DQ*; Fig. 3i). The ME5 module, associated with microglia and other myeloid cells (Fig. 3h), with hub genes *BTK* and *SYK*, consisted mainly of genes associated with damage-associated microglia^43^ (*SPP1*, *CLEC7A*, *CTSB*, *LPL*, *TREM2*), phagocytosis (*FCGR1A*, *FCGR3A*, *FCER1G*, *C1QA*, *MSR1*, *TREM2*), lipid metabolism (*PLIN2*, *PPARG*, *ALOX5*, *ALOX5AP*, *TBXAS1*, *APOC1*, *ACSL1*) and lysosomal processing (*CTSB*, *CTSS*, *CTSL*, *LYZ*, *VNN1*, *VAMP8*, *DPP4*, *TASL*). Module ME7 was related to the unfolded protein response and ER stress (*HSPA6*, *HSPH1*, *DNAJA1*, *XBP1*), while module ME15, containing *CD79A*, *CCL5* and *IGHG1* as hub genes, linked to adaptive immune cells, particularly B cells and plasma cells (Fig. 3h). Given the general poor correlation between mRNA and protein levels, we investigated whether the gene modules could be validated at the protein level. Microglia-related modules ME5 and ME12 were strongly replicated at the protein level, being significantly higher expressed in lesions with foamy microglia (Extended Data Fig. 5). Immunohistochemical analysis of two representative proteins of ME5, linked to lipid droplet and oxylipin formation (PLIN2 and TBXAS1, respectively), further supported these observations (Fig. 3j–n). Of note, LAMP1, a lysosomal marker, was increased in both foamy and nonfoamy MLs and partly overlapped with PLIN2 staining (Fig. 3l,m,o). Heat-shock protein module ME7 did, however, not replicate at the protein level. B cell module ME15 was reflected in the immunoglobulin levels measured by proteomics (Extended Data Fig. 6). We found that predominantly immunoglobulin G1 (IgG1) increased in lesions compared to CWM, and IgG1 levels were significantly higher in MLs with foamy compared to nonfoamy microglia (Extended Data Fig. 6). In addition, we observed significantly higher expression of immunoglobulin receptors and complement components and their receptors in lesions with foamy microglia (Supplementary Fig. 6), indicating increased immunoglobulin-mediated phagocytosis^11^. In summary, WGCNA, validated by proteomics, reveals that MLs with foamy microglia are characterized by markers of an enhanced adaptive immune response, increased phagocytosis, lysosomal activity, lipid storage and metabolism, and antigen presentation.

**Extended Data Fig. 7:**
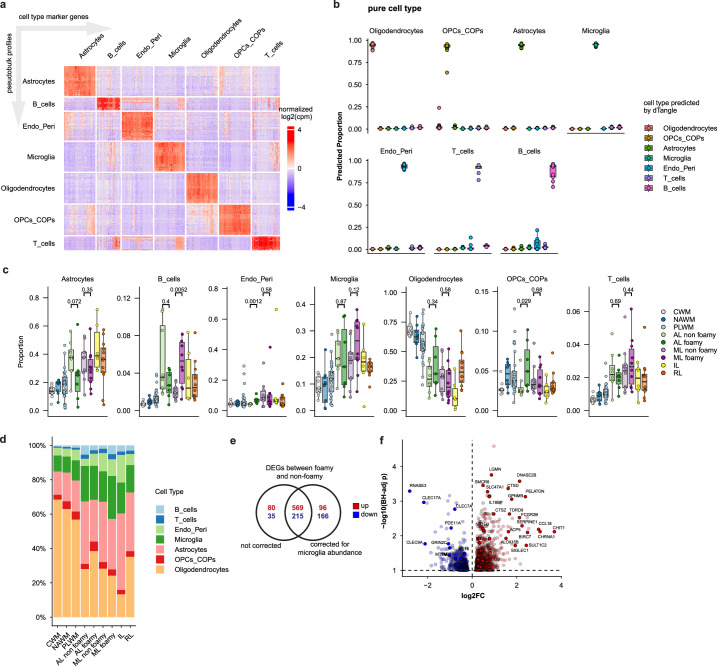
Single-cell deconvolution of the bulk RNA-seq data using snRNA-seq. **a**, Marker genes selected for each cell type for use in deconvolution. **b**, Validation of the method: the snRNA-seq was randomly split 80/20, and subsequently 80% of the data were used to predict the cell type composition of the remaining 20% of samples. For each cell type, we consistently see a very high percentage (~90%), validating the method. **c**, Box plots showing the fraction of each cell type across the different lesion types. Statistical test is the two-sided Wilcoxon rank-sum test without any correction for multiple testing. For panels **b** and **c**, data are expressed as individual values (circles), the median as the center, boxes representing the first and third quartiles and whiskers extending to datapoints within the 1.5× interquartile range. Number of replicates in **c** are specified in Table 1. **d**, Global cell type composition of each lesion type. **e**, Overlap between differentially expressed genes between foamy and nonfoamy samples with and without correction for the microglia numbers in **c**. P-values were calculated by limma (two-sided), including or excluding the log_2_-transformed microglia fractions as a covariate in the linear model. **f**, Volcano plot showing DEGs between foamy and nonfoamy samples with correction for the microglia numbers in **c**, calculated as in **e**.

To assess the cell-type composition of the lesions, we performed cell-type deconvolution of the bulk transcriptome using dtangle and a recently reported MS single-nucleus RNA-seq (snRNA-seq) dataset^24,44^. As expected, the number of oligodendrocytes was significantly lower in all lesions compared to CWM, while the number of astrocytes was increased (Extended Data Fig. 7). We also observed elevated B and T cell numbers, with B cells being most abundant in foamy MLs (Extended Data Fig. 7). This observation was confirmed by immunohistochemical analysis using the pan B cell marker CD79A, showing a high abundance of B cells in the perivascular space and infiltrating the parenchyma (Extended Data Fig. 6d–f). Microglia numbers increased across all lesions, with a tendency to be highest in MLs with foamy microglia (Extended Data Fig. 7). However, this increase did not explain the gene expression differences between foamy and nonfoamy samples, as most genes remained significantly differentially expressed after correcting for microglia numbers (Extended Data Fig. 7). This suggests that the observed WGCNA modules and differential expression are driven by gene expression specific to foamy microglia, rather than by a higher number of microglia.

### Foamy microglia represent a distinct microglial state

**Fig. 4:**
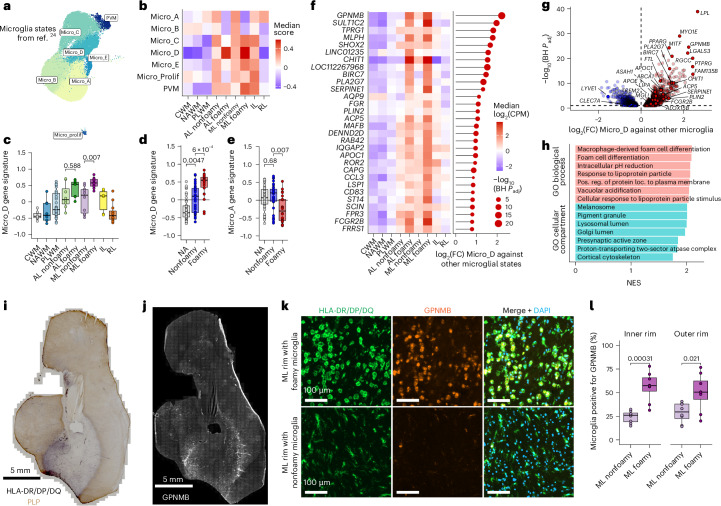
Foamy microglia represent a distinct microglial state. **a**, Microglial states in MS lesions obtained in ref. ^24^. **b**, Enrichment of the microglial state signatures from **a** in the bulk RNA-seq data using gene set variation analysis^94^. Replicate numbers are specified in Table 1. *P* values represent two-sided Wilcoxon rank-sum tests with BH adjustment for multiple testing. **c**, Micro_D gene signatures across lesion type. **d**, Micro_D gene signature across foamy or nonfoamy microglia. **e**, Micro_A gene signature across foamy or nonfoamy microglia. **f**, Top 30 Micro_D marker genes. Marker genes were filtered for microglia-selective genes (>50% counts originated from microglia). Left: the median log_2_(CPM) in the bulk RNA-seq dataset. Right: the log_2_(FC) of Micro_D marker genes against the combined other microglial states in the snRNA-seq dataset. *P* values were calculated by limma (two sided) and BH corrected for multiple testing. **g**, Differentially expressed genes between Micro_D against the other microglial states calculated by limma (two sided) and BH corrected for multiple testing (FDR < 10%). **h**, Pathways associated with Micro_D using GSEA. **i**,**j**, Adjacent sections from a representative ML with foamy microglia stained for HLA/PLP (**i**) or GPNMB (**j**). Representative images of eight independently analyzed lesions. **k**, Analysis of GPNMB (orange) and HLA-DR/DP/DQ (green) double staining in MLs with either foamy or nonfoamy microglia (*n* = 8 independent lesions for both groups). **l**, Quantification of GPNMB^+^ microglia is stratified into the inner rim (microglia within the demyelination border) and the outer rim (outside the demyelination border). In **c**–**e** and **l**, data are expressed as individual values (circles), the median as the center, boxes representing the first and third quartiles and whiskers extending to datapoints within the 1.5× interquartile range, and *P* values were calculated with two-sided Wilcoxon rank-sum tests on individual lesions. PVM, perivascular microglia; NES, normalized enrichment score. Source data

**Extended Data Fig. 8:**
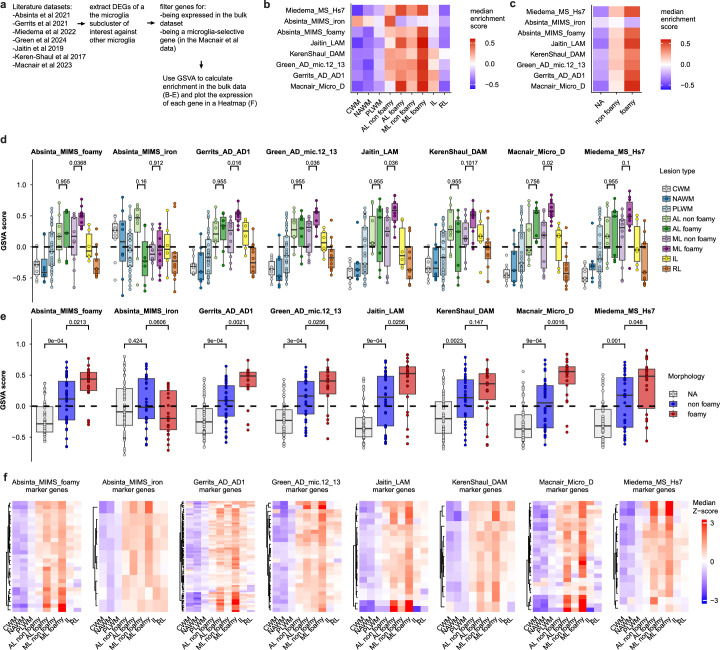
Literature microglia states associated with MS, AD and obesity are enriched in foamy MS lesions. **a**, Marker genes selection strategy based on refs. ^11,24,43,46–48^. **b**,**c**, Heatmaps showing the median enrichment score for each MS lesion type (**b**) or morphology (**c**) using GSVA. **d**,**e**, Box plots showing enrichment of each state using GSVA. Statistical test is the two-sided Wilcoxon rank-sum test with BH correction for multiple testing. For panels **d** and **e**, data are expressed as individual values (circles), the median as the center, boxes representing the first and third quartiles and whiskers extending to datapoints within the 1.5× interquartile range. The number of replicates are specified in Table 1. **f**, Median expression of state-marker genes in the different lesion types. Data are presented as z-scored log_2_ counts per million.

Given the specific molecular profile of foamy lesions, we investigated whether the foamy phenotype represents a distinct microglial transcriptional state^45^. To this end, we examined snRNA-seq-derived transcriptional microglial states reported in ref. ^24^ (Fig. 4a), determined gene signatures for each state and analyzed their expression in the bulk transcriptome, adjusted for total microglia abundance (Fig. 4b and Supplementary Fig. 7). We found that the Micro_D profile was significantly elevated in lesions with foamy microglia compared to nonfoamy, especially evident in MLs (Fig. 4c,d). Conversely, the homeostatic cluster Micro_A was significantly reduced in lesions with foamy microglia but not in those with nonfoamy microglia (Fig. 4e). There was considerable overlap between the Micro_D profile and previously reported microglial states, such as the MIMS-foamy state reported in ref. ^11^, amyloid-associated microglia in Alzheimer’s disease^43,46,47^ and lipid-associated macrophages (LAMs) in obesity^48^ (Extended Data Fig. 8). The Micro_D profile was characterized by marker genes such as *GPNMB*, *CHIT1*, *APOC1*, *PLIN2* and *ACP5* (Fig. 4f,g and Supplementary Fig. 8). Consistent with this, ontologies associated with the Micro_D state were lipid storage, phagocytosis, foam-cell differentiation and lysosomal compartment (Fig. 4h), but not pro-inflammatory signaling. This lack of classical pro-inflammatory signaling has been previously found in MS lesions by mass cytometry of microglia^49^ and matches the lipid-induced lysosomal phenotype described in other diseases^14,18,50,51^. Micro_D marker genes were also significantly enriched in lesions with foamy microglia at the protein level (Supplementary Fig. 9). *GPNMB*, a marker for lysosomal stress^40^ and previously characterized to be highly expressed in ALs^52^, emerged as the strongest Micro_D marker gene (Fig. 4f). Therefore, we performed double immunofluorescence staining on GPNMB to locate Micro_D microglia in MS lesions. GPNMB^+^ microglia were found in the rims of MLs with foamy microglia, but not in the lesion core (Fig. 4i,j). GPNMB-positive cells were significantly higher in MLs with foamy microglia compared to nonfoamy (Fig. 4k,l). This suggests that Micro_D GPNMB^+^ microglia, with markers for increased phagocytosis, lipid metabolism and lysosomal stress, represent a distinct microglial state associated with chronic lesion expansion.

### Multi-omics integration captures a molecular axis that associates with disease progression

**Fig. 5:**
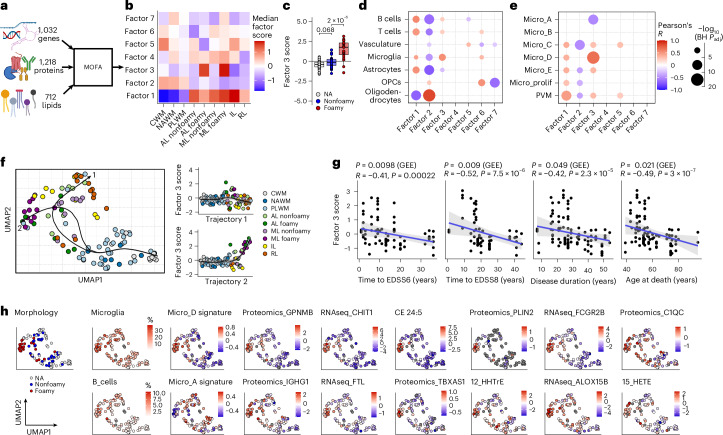
Multi-omics data integration reveals an integrated molecular profile of foamy microglia that associates with disease progression. **a**–**c**, Overview of the number of genes, proteins and lipids as input for MOFA (**a**), which delivered seven factors based on a minimum 5% variance explained cutoff depicted in a heatmap (**b**). Factor 3 significantly associates with foamy lesions (**c**). In **c**, data are expressed as individual values (circles), the median as the center, boxes representing the first and third quartiles and whiskers extending to datapoints within the 1.5× interquartile range, and *P* values were calculated with two-sided Wilcoxon rank-sum tests on individual lesions with BH correction for multiple testing. Replicates are specified in Table 1. **d**,**e**, Correlation of MOFA factors with broad cell types (**d**) and specific microglial states (**e**). Data are presented as Pearson’s *R*, with size indicating BH-adjusted *P* values. **f**, Uniform manifold approximation and projection (UMAP) with a pseudotime trajectory analysis using the MOFA factors as input. Two trajectories were observed, differentiated by factor 3 scores. Error bands represent the 95% confidence interval of a loess-fitting curve. **g**, MOFA factor 3 was associated with clinical features in a generalized estimating equation (GEE) model to account for donor identity. In addition, Spearman correlation coefficients (*R*) are given with their respective *P* values to assess variance explained by the association. Error bands indicate the 95% confidence interval. **h**, UMAP projection the same as in **f**, but colored by important variables that are associated strongly with factor 3. For cell types, the color depicts percentage, and, for Micro_A and Micro_D, it represents the gene set variation analysis score (from Fig. 4b–e). For all other plots, each variable is expressed as the normalized log_2_-transformed value of its respective unit. Panel **a** created in BioRender; Van der Vliet, D. https://biorender.com/a5ghuvi (2026).

Next, to obtain an integrated molecular profile that we could link to disease progression, we performed multi-omics factor analysis (MOFA)^53^ (Fig. 5a–h and Supplementary Fig. 10). MOFA derives latent variables from covariance in multimodal data, expressed as factors. We found that MS lesions in general, characterized by myelin loss, were captured by factors 1 and 2, whereas lesions containing foamy GPNMB^+^ microglia, represented by the Micro_D gene signature, were strongly associated with factor 3 (Fig. 5b–e). Factor 3 contained increased CE, BMP and oxylipin levels along with their oxylipin-generating enzymes ALOX15B and TBXAS1, as well as IgG1, C1QC and *FCGR2B*, while axonal and synaptic proteins were downregulated^19^ (Fig. 5h and Supplementary Figs. 11 and 12).

A slingshot pseudotime trajectory analysis^54^ revealed that factor 3 was a strong driver for the separation of RLs from foamy MLs, while ALs and MLs with nonfoamy microglia were more closely related to RLs (Fig. 5f). Notably, factor 3 also associated with more severe disease progression, as evidenced by shorter times to EDSS6 and EDSS8 and reduced disease duration (Fig. 5g). Taken together, this indicates that foamy GPNMB^+^ microglia are a main source of variability in the molecular profile of MS lesions, which associates with more severe hallmarks of pathology and disease course.

### Activity-based protein profiling uncovers MAGL as a target to improve lesion recovery

**Extended Data Fig. 9:**
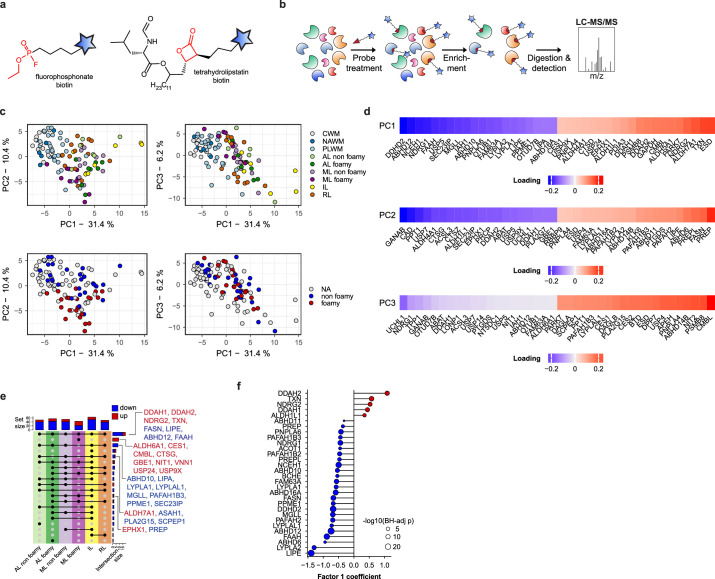
Activity-based protein profiling of MS lesions. **a**, Probes used for ABPP in this study. **b**, Schematic workflow of the ABPP method. Lysates are incubated with the probes from **a**, and subsequently targeted proteins are enriched from the mixture and analyzed by mass spectrometry. **c**, Principal component analysis (PCA) of the ABPP data colored by lesion type or morphology. **d**, Top 20 positive and negative loadings from the ABPP PCA in **c**. **e**, UpSet plot shows overlap of differentially active enzymes in the different lesion type compared to NAWM. **f**, Association of enzyme activities with MOFA factor 1. A linear model of the seven MOFA factors was fitted to the ABPP data, identifying enzymes significantly associated with factor 1. Statistics in **e** and **f** were calculated using limma (two-sided) with BH correction for multiple testing and an FDR < 10%.

To identify potential drug targets to address MLs with foamy microglia, we performed activity-based protein profiling (ABPP), a chemical biology technique that identifies active enzymes in their native biological context^55,56^ (Extended Data Fig. 9). Because the foamy phenotype was characterized by increased lipid processing and oxylipin production, we performed ABPP using fluorophosphonate and β-lactone chemical probes^57,58^ targeting serine and cysteine hydrolases (Extended Data Fig. 9), which have a crucial role in lipid and protein metabolism^55,59^.

**Fig. 6:**
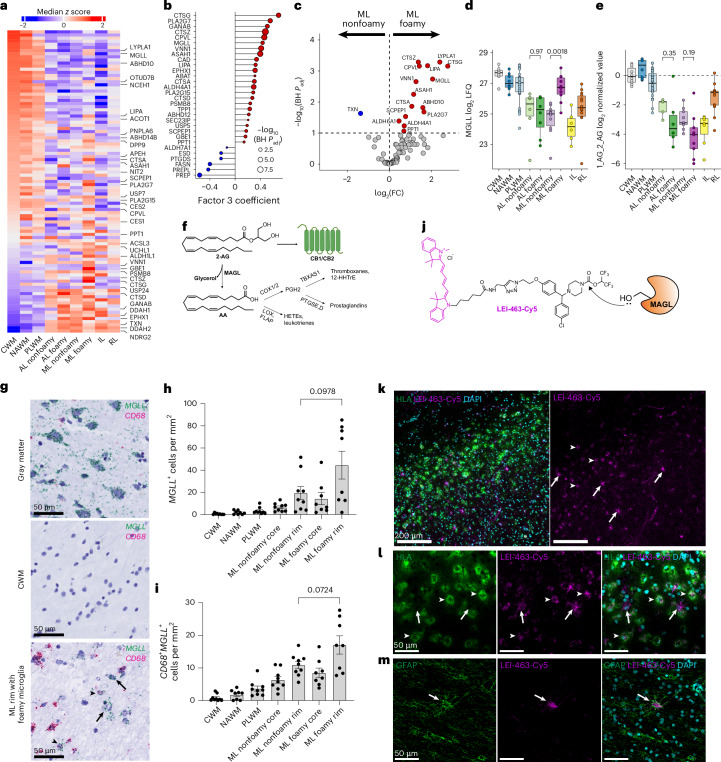
Foamy MLs have high mono-acyl glycerol lipase (*MGLL*) activity. **a**, ABPP detected 97 enzyme activities. The heatmap depicts median *z*-scored LFQ values, sorted on CWM mean values. **b**, Association of enzyme activities with MOFA factor 3. A linear model of the seven MOFA factors was fitted to the ABPP data, identifying enzymes associated with factor 3. **c**, Differentially active enzymes between MLs with foamy microglia to nonfoamy. **d**, MGLL activity across lesion types. *P* values are from **c**. **e**, MGLL-substrate 2-AG levels across lesion types show a trend for reduction in ML with foamy microglia compared to nonfoamy. In **d** and **e**, data are expressed as individual values (circles), the median as the center, boxes representing the first and third quartiles and whiskers extending to datapoints within the 1.5× interquartile range. Replicates are specified in Table 1. For **b**–**e**, *P* values were calculated by limma (two sided) and BH corrected for multiple testing (FDR < 10%). **f**, Schematic representation of MAGL-mediated hydrolysis to AA, an oxylipin precursor. **g**–**i**, Double ISH of MAGL transcripts (*MGLL)* and *CD68* as a microglial marker in gray matter, CWM and ML rim (**g**) with quantification of all MGLL^+^ cells (**h**) and MGLL^+^ CD68^+^ double positive cells (**i**). Data are expressed as mean ± s.e.m. with individual data points (circles) overlaid. *P* values were calculated by a Welch and Brown–Forsythe analysis of variance test in GraphPad Prism 10. CWM, *n* = 9; NAWM, *n* = 8; PLWM, *n* = 9; ML nonfoamy, *n* = 9; ML foamy, *n* = 8. **j**, Structure of LEI-463-Cy5, a fluorescence probe to visualize active MAGL. **k**, Representative image of a foamy ML stained with LEI-463-Cy5 (magenta) and an antibody against HLA (green). **l**,**m**, Close-up image of the colocalization of LEI-463-Cy5 and HLA (**l**) or GFAP (**m**). Arrows indicate MAGL-positive astrocytes, while arrowheads indicate MAGL-positive microglia. The images in **k** and **l** are representative of five independent lesions stained with LEI-463-Cy5. Source data

**Extended Data Fig. 10:**
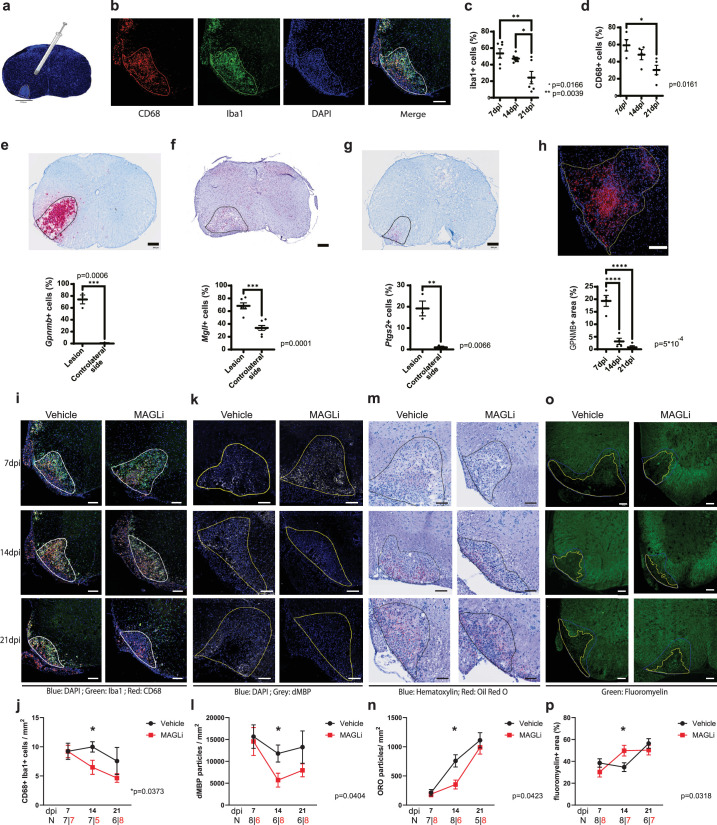
MAGL inhibition improves lesion progression and resolution. **a**, Location of LPC injection in the mouse spinal cord. **b**, LPC-induced lesion at 7 days post injection (dpi) shows microgliosis marked by staining for CD68 and Iba1. **c**, Quantification of the number of Iba1-positive cells at 7, 14 and 21 dpi. 7dpi N = 6 mice, 14 dpi N = 7, 21 dpi N = 6 mice. **d**, Quantification of the number of CD68-positive cells at 7, 14 and 21 dpi. 7 dpi N = 4 mice, 14 dpi N = 4, 21 dpi N = 5 mice. **e**–**g**, In situ hybridization of *Gpnmb* (**e**, N = 3 mice), *Mgll* (**f**, N = 7 mice) and *Ptgs2* (**g**, N = 3 mice) mRNA in LPC-induced lesions at 7 dpi with quantification. The lesion injection site was compared to the contralateral mirrored area (two-sided Student’s t-test). **h**, GPNMB protein levels in LPC-induced lesions. GPNMB-positive area was quantified at 7, 14 and 21 dpi (N = 4 mice per time point, Student’s t-test compared to 7 dpi). **i**–**p**, Mouse spinal cords were injected with LPC to induce demyelinating lesions, and subsequently the mice were treated with either vehicle or MAGL inhibitor MAGLi-432 (MAGLi, 5 mg kg^−1^) daily from 2 dpi until the indicated time point. Afterward, the lesions were analyzed for microgliosis (CD68, Iba1) (**i**–**j**), myelin debris staining by a dMBP antibody (**k**–**l**), Oil Red O staining (**m**–**n**) and myelin content by fluoromyelin (**o**–**p**). All p-values represent two-sided Student’s t-tests. Data are expressed as the mean ± s.e.m. with individual data points overlaid (circles). For panels **j**, **l**, **n** and **p**, replicates are indicated in the figures and represent independent mice. Split color images in **i** and **k** are shown in Supplementary Fig. 15. Scale bars = 100 µm (**b**, **h, i, k, m** and **o**). Scale bars = 200 µm (**e**–**g**). Source data

We identified 97 active hydrolases in MS lesions, of which many were downregulated (Fig. 6a and Extended Data Fig. 9e,f), potentially reflecting the loss of axons and oligodendrocytes, and key lipid-metabolizing cells. Several metabolic enzyme activities associated with MOFA factor 3 and were upregulated in MLs with foamy microglia (Fig. 6a–c). Notably, these included the lysosomal enzymes ASAH1, LIPA, CTSA, CTSG, CTSZ, PLA2G15 and PPT1, enzymes part of the Coordinated Lysosomal Expression and Regulation network^60^, as well as *PLA2G7*, previously associated with atherosclerotic plaques^61^ and with the LAM^48^ and MIMS-foamy microglial states^11^. Notably, monoacylglycerol lipase (MAGL) activity, an enzyme associated with lipid droplets^62^, showed an intriguing dynamic profile. Its activity was reduced in all lesion types compared to CWM and NAWM, reflecting oligodendrocyte and axonal loss, but its activity was restored in MLs with foamy microglia with approximately fourfold increase compared to MLs with nonfoamy microglia (Fig. 6c,d). MAGL activity also significantly associated with MOFA factor 3 (Fig. 6b), and its mRNA levels were highest in the Micro_D cluster (Supplementary Fig. 8f), suggesting expression of MAGL directly by foamy microglia. MAGL catalyzes the conversion of the endocannabinoid (eCB) 2-arachidonoylglycerol (2-AG) into arachidonic acid (AA) in the brain, which is a precursor for oxylipins^33,63^ (Fig. 6f and Supplementary Fig. 13). Consistent with this, 2-AG levels were the lowest in MLs with foamy microglia (Fig. 6e), where oxylipins were significantly increased (Fig. 2h). Thus, we selected MAGL for further validation. Double in situ hybridization (ISH) of the MAGL-encoding gene *MGLL* and *CD68* as a microglial marker confirmed that *MGLL* transcripts were highest in MLs with foamy microglia (Fig. 6g–i), whereas *MGLL* expression was absent in CWM and NAWM (Fig. 6g,h). Activity-based histology with an LEI-463-Cy5 as a MAGL-specific fluorescence probe that only labels active enzyme^64^ (Fig. 6j) confirmed that MAGL activity was found in HLA-positive foamy microglia as well as in astrocytes in the rim of MLs (Fig. 6k–m). To test whether inhibition of MAGL activity has therapeutic potential, we examined the effects of the MAGL-inhibitor MAGLi-432 (ref. ^65^) in a lysophosphatidylcholine (LPC)-induced focal demyelination model of the mouse spinal cord (Extended Data Fig. 10), which emulates the microglial phenotypes observed in lesions with foamy microglia^66,67^. Consistent with this, the expression of *Gpnmb* was strongly induced in these spinal cord lesions (Extended Data Fig. 10e,h). In addition, *Mgll* and *Ptgs2* expression was induced in these lesions (Extended Data Fig. 10f,g), indicating an upregulation of oxylipin biosynthetic enzymes. Treatment with MAGLi-432 from 2 days post-LPC-injection (dpi) reduced microgliosis and lipid accumulation, improved myelin debris clearance and lesion recovery at 14 dpi (Extended Data Fig. 10i–p), indicating a functional role for MAGL in lesion progression and recovery.

### CSF oxylipins associate with the presence of foamy microglia

**Fig. 7:**
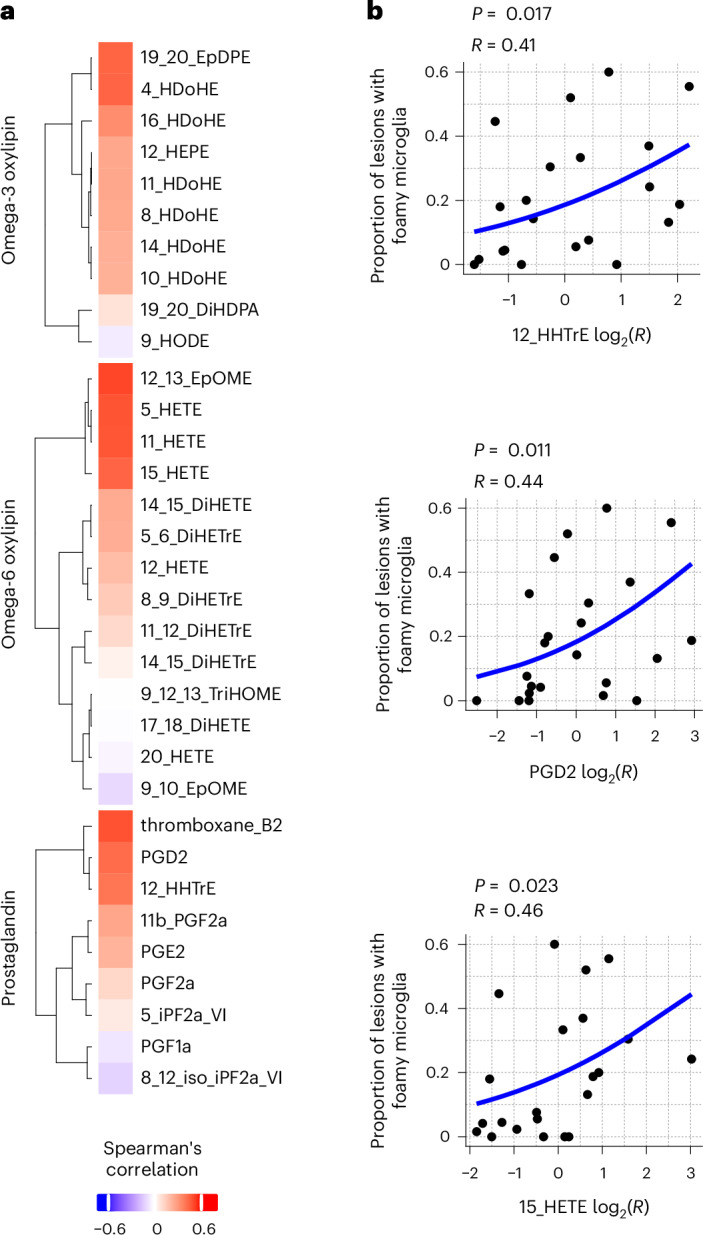
CSF oxylipin levels are associated with a higher foamy microglial load. **a**, Spearman correlations of CSF oxylipin levels to the proportion of foamy lesions in patients. To obtain the proportion of foamy lesions, the number of WM lesions classified as foamy was divided by the total number of WM lesions found in the brain of the donor, similar to the analysis in Fig. 1g. **b**, Linear models showing the significant association across selected oxylipins to the proportion of foamy lesions. *P* values represent likelihood ratio tests after fitting a quasibinomially distributed generalized linear model. In addition, Spearman correlation coefficients (*R*) are given to assess the variance explained by the association.

Finally, because oxylipins and prostaglandins are excreted factors, we explored whether these lipids could be detected in the CSF of the same cohort of MS donors. While there was no statistical difference between the control and MS donors in oxylipin levels, potentially due to age as a confounding factor^68^, CSF levels of many oxylipins positively correlated with the proportion of foamy lesions in their brain (Fig. 7a,b). For example, AA-derived oxylipins 12-HHTrE, PGD2 and 15-HETE, which are strongly associated with MOFA factor 3 (Fig. 5h and Supplementary Fig. 14), significantly correlated with the proportion of lesions with foamy microglia. These results may suggest that oxylipins produced by foamy microglia or infiltrating macrophages can be detected in the CSF.

## Discussion

Understanding the drivers of chronic lesion expansion in progressive MS remains a critical unmet challenge. While much is known about immune mechanisms underlying early relapsing-remitting stages of MS, the pathological basis of irreversible progression remains poorly defined. One hallmark of progressive MS is the presence of slowly expanding demyelinating lesions with smoldering inflammation at their borders, often characterized by iron rims^6,7,11,13,69^. Despite their strong association with neurological decline, the molecular and cellular mechanisms that distinguish expanding lesions from resolving or remyelinated ones remain elusive. In particular, the role of lipid metabolism and microglial heterogeneity in chronic lesion pathology has been understudied^3^.

In this study, we addressed this gap by integrating histological, lipidomic, transcriptomic, proteomic and chemical proteomic analyses of human postmortem MS WM lesions. We identified a distinct population of foamy, GPNMB^+^ microglia/macrophages associated with lesion pathology and disease progression. These cells, enriched at the rims in some mixed active/inactive lesions and throughout some ALs, exhibit a molecular profile of lipid accumulation, lysosomal stress, sustained phagocytosis and antigen presentation, but lack classical pro-inflammatory gene expression, which may potentially reflect metabolic reprogramming associated with the chronic nature of the lesion^14,18,50^. Not every MS donor had MLs and ALs with foamy microglia at the time of death, and the proportion of lesions with foamy microglia varied strongly between individuals (Extended Data Fig. 1c), revealing a potential source of heterogeneity between MS donors. Across 250 MS donors, we found that the proportion of lesions with foamy microglia correlated with faster progression to high EDSS scores, while lesions with nonfoamy microglia were not associated with fast disease progression. This finding may suggest that the foamy phenotype is not simply a byproduct of demyelination but a defining feature of lesion persistence and expansion. By contrast, lesions with nonfoamy microglia or those undergoing remyelination did not show these lipid-rich features. Thus, the classification of lesions by the foamy phenotype, profiled across multiple molecular platforms, emerged as a robust and clinically meaningful marker of lesion characteristics and disease course. Future mechanistic studies in in vitro and in vivo models may help to establish whether foamy microglia/macrophages are key drivers of disease progression.

Through MOFA, we identified a latent molecular axis (MOFA factor 3) that captured the integrated transcriptomic, proteomic and lipidomic signature of lesions with foamy microglia. This axis featured elevated CEs, BMPs and oxylipins, as well as enhanced expression of Fcγ receptors, complement components, and antigen presentation machinery. Lesions rich in foamy GPNMB^+^-microglia also exhibited high local levels of IgG1 and plasma cell infiltration, potentially suggesting a possible mechanism of antibody-driven hyperphagocytic activation^70,71^. While CD20-targeted therapies deplete peripheral B cells, they fail to resolve chronically expanding lesions, possibly due to the presence of terminally differentiated CD20^−^ antibody-secreting cells in the lesion parenchyma^72^. In contrast, brain-penetrant BTK inhibitors, which target both B cells and myeloid cells, may better modulate this lesion compartment and recently showed a promising reduction in disability progression^73,74^.

Using ABPP, we identified MAGL as a highly active enzyme in lesions with foamy microglia, with expression enriched in activated microglia. MAGL is the main enzyme in the brain responsible for the hydrolysis of >85% 2-AG into AA, a precursor of bioactive oxylipins^63,75^. These oxidized signaling lipids act as chemoattractants for adaptive immune cells, potentially driving T cell and B cell infiltration^33^, enhancing T_H_17 cell^76^ and plasma cell differentiation, and increasing immunoglobulin production, collectively contributing to neuroinflammation and a sustained demyelinating milieu^63,77–81^. Inhibition of MAGL leads to elevated 2-AG levels and reduced production of free AA and oxylipins. Increased 2-AG activates cannabinoid receptors CB1 and CB2 (refs. ^82,83^), which reduces excitotoxicity^84,85^, dampens autoimmunity^86^ and promotes a pro-repair, neuroprotective phenotype^87,88^. Consistent with these mechanisms, MAGL inhibition has been shown to ameliorate experimental autoimmune encephalomyelitis and cuprizone-induced demyelination by reducing excitotoxicity, gliosis and T cell infiltration, while enhancing remyelination^89–92^. Here we demonstrated that MAGL inhibition in a preclinical model of focal demyelination also promoted lesion recovery, reduced microgliosis and improved myelin debris clearance. Notably, treatment with the MAGL inhibitor was started 2 days after lesion induction, making sure that the observed outcomes were driven by better lesion recovery, not by preventing lesion formation. Together, these findings suggest that targeting MAGL may offer a disease-modifying approach for MS progression by promoting lesion resolution and repair rather than merely providing symptomatic relief through indirect cannabinoid receptors activation^93^. MAGL inhibitors are currently being evaluated in clinical trials (for example, NCT06782490), providing a timely opportunity to test this therapeutic strategy in patients.

The foamy, GPNMB^+^ microglial phenotype in MS lesions bears striking transcriptional similarity to LAMs in obesity, amyloid-responsive microglia in Alzheimer’s disease and foam cells in atherosclerosis^18,46,48,50,51^. These states converge on common features—lipid accumulation, lysosomal overload and chronic activation without classical inflammation^18^. Our findings reinforce the concept of a shared, maladaptive microglial activation program that contributes to chronic tissue injury in diverse pathologies. This shared biology suggests that therapeutic strategies targeting lipid metabolism, such as MAGL or other lipid-metabolizing enzymes, may have cross-disease applicability and could be harnessed to modify progression in MS and beyond. In parallel, biomarkers such as GPNMB^40,52^, CHIT1 (ref. ^42^), and oxylipins in CSF or plasma^68^ may help stratify patients and monitor treatment response.

While our study provides a comprehensive multi-omics characterization of MS WM lesions, several limitations should be acknowledged. First, our analyses are based on postmortem human tissue, which offers valuable pathological insights but reflects static end-stage molecular profiles rather than dynamic disease processes. As such, temporal relationships, such as the transition from nonfoamy to foamy microglia or causal relationships between lipid accumulation and lesion expansion, remain inferred rather than directly observed. Second, although our integrative approach enables robust molecular profiling, bulk transcriptomic and proteomic data cannot fully resolve cellular heterogeneity or spatial context. While we validated key findings with immunohistochemistry and single-nucleus transcriptomics reference datasets, further spatial transcriptomic or single-cell studies will be important to disentangle the interactions between microglia, B cells, and other CNS-resident or infiltrating cells within lesions. Third, our functional validation was limited to a preclinical toxin-induced demyelination model, which recapitulates aspects of lesion pathology but lacks the full spectrum of MS-related immune responses. Future studies in human-derived systems or clinical trials will be critical to test the translational relevance of targeting MAGL and other lipid pathways in foamy microglia.

In conclusion, this study establishes that foamy GPNMB^+^ microglia/macrophages are associated with expanding lesions and progressive disease in MS. Their metabolic and immune phenotypes suggest the presence of a unique pathophysiological mechanism, independent of classical inflammation, that sustains lesion activity and offers new therapeutic targets. By focusing on microglial lipid metabolism, we outline a strategy to interrupt chronic lesion progression and advance treatment options for progressive MS.

## Methods

### Human brain tissue

Human brain samples from 38 donors (28 secondary progressive MS and 10 matched nondemented controls) were obtained from the NBB (brainbank.nl, project 1327). All procedures of the NBB were approved by the Medical Ethical Committee of Amsterdam Academic Medical Centre, and all donors gave written consent to the NBB for the use of their data and tissue for research. Fresh-frozen tissue blocks containing subcortical WM with or without MS lesions were dissected, snap-frozen in liquid nitrogen and stored at −80 °C. Mirrored pieces of this tissue were formalin fixed and paraffin embedded and stained to characterize different MS lesion stages (mixed active/inactive, active, inactive, remyelinated) by the NBB. Selection criteria for donors were a postmortem delay (time interval between the demise of the donor and freezing of the tissue) below 12 h, a pH of the CSF higher than 5.5 and a clinician-confirmed diagnosis of secondary progressive MS for patients with MS and the absence of any neurological disease in controls, nor neurological disease-indicating pathology found in their brains as examined by a neuropathologist. The postmortem delay ranged between 6.5 to 11.5 h, with a median of 8.75 h. The pH of the CSF ranged from 5.8 to 6.8, with a median of 6.4. Donor characteristics were matched as closely as possible (Extended Data Fig. 1a), but there were differences in age between controls and MS donors.

### Lesion characterization and definitions

Lesions were classified using our previously defined criteria^1,6^ in line with the classification system mentioned in ref. ^11^. In short, CWM is WM from nondemented control donors, absent any sign of demyelination or aberrant microglial activation. NAWM is macroscopically intact WM from a patient with MS, and within the same tissue block, no lesion or inflammation is visible. PLWM is WM adjacent to a WM lesion. Depending on the lesion type the PLWM is adjacent to, sometimes there is considerable inflammation, or even the presence of foamy microglia. ALs (type 2, also called acute) were defined as (partially) demyelinated areas with increased microglial presence throughout the lesion and no hypocellular core. MLs (type 3, also called mixed, chronic active, smoldering or slowly expanding) were defined as completely demyelinated areas with a hypocellular gliotic core, with an active rim of macrophages at the border of demyelination. Inactive lesions (IL, type 4) were characterized by complete demyelination, the absence of an active HLA^+^ rim and a hypocellular gliotic core. Remyelinated lesions (RL, type 6, also called shadow plaques) were characterized by partial myelination. In addition, residual myelin should not only be myelin debris, but also axonal myelin. Microglial activation was sometimes still present, but not to a larger density of cells than the surrounding PLWM. In addition to these classically defined lesion types, we profile the morphology of the microglia found in the AL and ML^66^. AL and ML with either foamy or ramified and ameboid (together called nonfoamy) microglia were distinguished, in line with our previously published work^6,17^.

### Analysis of lesion proportions and clinical features

Clinical and pathological data were obtained from the NBB for MS brain donors collected between 1990 and 2021. All lesions identified by macroscopic inspection, or on postmortem magnetic resonance imaging appearance, throughout the brain and in three standard locations in the brain stem and spinal cord were pathologically characterized as described above and are analyzed for the clinical correlations. In total, 8,708 lesions were analyzed in 250 MS donors (average of 34.8 lesions/donor). Proportions of lesions were defined as counts of lesions of interest divided by all WM lesions. Time to EDSS6 and EDSS8 was defined as the year in which EDSS6 or EDSS8 was reached minus the year in which the first symptoms were reported. Disease duration was defined as the number of years between the first reported symptoms and death. Lesion counts were correlated to clinical features using a generalized linear model with a quasibinomial distribution of counts of lesions against other lesions, as previously described in ref. ^6^. *P* values to test the model were calculated using the likelihood-ratio test and corrected for multiple testing using the Bonferroni method.

### Statistics, randomization, blinding and differential expression

No power analysis was performed to predetermine sample sizes, but our sample sizes are similar to those reported in previous publications^24,25,95^. Samples were always randomized before analysis. Researchers were not blinded to classifications of samples, because our measurements are quantitative and not sensitive to subjective interpretation. A replicate refers to a unique lesion isolated. Never in this study is the same lesion measured repeatedly. The number of unique lesions isolated per lesion classification is specified in Table 1. Not every lesion was successfully analyzed by all techniques described in this study; missing lesions are specified in Table 1 as well. No datapoints have been excluded after obtaining the data. The only reason a datapoint is not in the analysis is the failure to obtain it.

All differential expression/abundance analyses on -omics analyses were performed using the limma linear modeling framework^96^. Normality and equal variances were not formally tested.

For the RNA-seq data, we constructed a linear model taking into account covariates, using the formula *y* = ~0 + lesion type + RQN + sex, with lesion type being the design and RQN (RNA quality number) and sex being covariates. We ran limma’s linear modeling function ‘lmFit’ using the modeling design as above, using ‘voom’ for precision weight analysis^97^. For all other datasets, we ran the lmFit function on log_2_-expression values without ‘voom’ and without taking any covariates along in the model (*y* = ~0 + lesion type).

For calculating differential expression between foamy and nonfoamy microglia, which can appear in several backgrounds (MLs, ALs and also PLWM), we constructed a linear model using lesion type as a covariate, without specifying the morphology (seven groups—CWM, NAWM, PLWM, AL, ML, IL and RL) and specifying the morphology as separate parameters. For the RNA-seq, the linear model was *y* = ~0 + morphology + lesion type (seven groups) + RQN + sex, and for all the other datasets, the linear model was *y* = ~0 + morphology + lesion type (seven groups).

These linear models were fed into the limma eBayes algorithm with default settings. Thus, log_2_ fold changes and Benjamini–Hochberg (BH) corrected *P* values were extracted using the TopTable function (two-sided tests). We generally accept a false discovery rate (FDR) of 10%, meaning that we consider variables with a BH-adjusted *P* value lower than 0.1 statistically significant.

### Cryosectioning and isolation of MS lesions

Fresh-frozen tissue was sectioned at 10 μm for stainings or 50 µm for tissue collection in a cryostat (Thermo Fisher Scientific, HM525 NX) between −20 °C and −16 °C with Thermo Fisher Scientific MX35 Ultra microtome blades. Sections for stainings were mounted on Epredia Superfrost slides (J1800AMNZ), dried overnight using silica beads and subsequently sealed and stored at −80 °C until further use. Borders of a demyelinated lesion were identified based on hematoxylin and Sudan Black staining and inspection under a light microscope. A surgical scalpel was used to demarcate the lesioned area, after which the lesion and peri-lesion were separated from 50 µm sections in a cryostat. Continuous-stained sections were prepared to ensure the lesion borders were closely matched by the isolation method. Before and after every sample collected a slide, it was prepared for histological confirmation of lesion pathology. Tissue was collected in 1.5 ml sterile RNase-free SafeLock Eppendorf tubes, weighed and stored at −80 °C. Samples isolated for RNA extraction were stored in Trisure (Bioline, BIO-38032).

### Immunohistochemistry (single staining, CD79A, GFAP, CD3, TBXAS1)

Frozen sections were taken from the −80 °C and warmed up to room temperature before opening the seal. The sections were then incubated in 4% PFA in PBS for 30 min, followed by peroxide blocking (1% H_2_O_2_, 0.5% Triton X-100 in PBS) for 20 min. The sections were subsequently incubated overnight at 4 °C with one of the following primary antibodies: GFAP (1:1,000, D1F4Q, Cell Signaling Technology), CD79A (1:200, M705001-2, DAKO), CD3 (1:100, A0452, DAKO) or TBXAS1 (1:200, 160715, Cayman Chemical) in PBS containing 1% BSA, 0.5% Triton X-100 and 10% horse serum. Subsequently, secondary antibody was applied from the DAKO REAL EnVision Kit (DAKO, K500711-2) for 1 h at room temperature. Horseradish peroxidase immunoreactivity was then visualized with 3,3′-diaminobenzidine (DAB) using the same kit according to the manufacturer’s instructions (substrate was diluted 1:50 in buffer). Then, the sections were washed and stained in hematoxylin solution (50 g l^−1^ KAl(SO_4_), 1 g l^−1^ hematoxylin, 0.2 g l^−1^ NaIO_3_, 50 g l^−1^ chloral hydrate, 1 g l^−1^ citric acid). After washing, the sections were dehydrated in a dehydration series of 50% EtOH (3 min), 70% EtOH (3 min), 96% EtOH (5 min), 100% EtOH (5 min), 100% EtOH (5 min), xylene (10 min) and xylene again (10 min). Then, the sections were dried and mounted in Entellan (Merck Millipore, 1079600500) using 24 × 50 mm coverslips (Corning, CLS2975245). Sections were imaged on a Zeiss Axioscan 7 and further processed using QuPath software (v0.5.0)^98^.

### Immunohistochemistry (double staining, HLA^+^ PLP)

Sections were fixed and peroxide blocked as described above and subsequently incubated in primary antibodies against HLA-DR/DQ/DP (1:1000; DAKO, M0775) in 1% BSA, 0.5% Triton X-100, 10% horse serum in PBS for 1 h at room temperature. Subsequently, the DAKO REAL EnVision Kit (DAKO, K500711-2) was applied for 1 h at room temperature. Horseradish peroxidase immunoreactivity was then visualized using DAB/Ni^2+^ (0.5 mg ml^−1^ DAB tetrahydrochloride, 2 mg ml^−1^ nickel ammonium sulfate, 0.01% H_2_O_2_ in PBS) for ~10 min. Subsequently, a PLP antibody was incubated (1:1,000, clone plpc1; Bio-Rad, MCA839G) in 1% BSA, 0.5% Triton X-100, 10% horse serum in TBS overnight at 4 °C. The next day, DAB staining was developed and the sections were mounted as described for the single staining.

### Immunofluorescence stainings and analysis (GPNMB, PLIN2, LAMP1)

For GPNMB and HLA, frozen sections were used; these were taken from the −80 °C and warmed up to room temperature, before fixing in 4% PFA in PBS for 30 min.

Stainings for LAMP1 and PLIN2 were performed in formalin-fixed paraffin-embedded tissue sections (8 µm). Formalin-fixed paraffin-embedded sections were deparaffinized in xylene and subsequently rehydrated through a series of ethanol to demineralized water, followed by antigen retrieval in citrate buffer of pH 6 in the microwave at 700 W for 14 min.

Subsequently, sections were blocked (10% donkey serum, 0.5% Triton X-100 in PBS) for 2 h at room temperature. Sections were subsequently incubated with primary antibodies against GPNMB (1:500; Cell Signaling Technology, E4D7P), HLA-DR/DQ/DP (1:1,000; DAKO, M0775), LAMP1 (1:200; Abcam, ab24170) and PLIN2 (1:200; Progen Biotechnik, GP40), in 0.5% Triton X-100, 5% donkey serum in PBS, overnight at 4 °C. Appropriate secondary antibodies (1:1,000; Jackson Immuno, donkey anti-IgG (H + L)) were incubated subsequently for 2 h at room temperature in PBS with 0.5% Ttriton X-100. Autofluorescence was quenched by incubating the section in 0.5% Sudan Black B in 70% ethanol for 2 min. Subsequently, the sections were briefly rinsed in 50% ethanol and then in PBS. Nuclei were stained with 1 µg ml^−1^ DAPI (Sigma) in PBS and the sections were mounted under a coverglass (Corning) in mounting medium (100 mg ml^−1^ Mowiol 4-88, 25% glycerol, 25 mg ml^−1^ DABCO in 0.1 M Tris–HCl, pH 8.5).

Images were captured using an Axioscan 7 slide scanner (Zeiss), and quantification of positive cells in each lesion was performed using QuPath (v0.5.0). Regions of interest (ROIs) were selected based on demyelination and the border of microglia/macrophages in adjacent HLA/PLP stainings. Then, analysis of HLA and GPNMB was performed using ‘Cell detection’ and ‘Object classifier’ commands. Cell detections were performed within the ROI using DAPI staining to detect cellular nuclei. A Random trees classifier was trained to separate microglia/macrophages from other detections. Objects were filtered for ‘cells’, and all features with the correct channel were selected. Approximately 50 points for each class were annotated by hand. The classifiers were loaded and applied sequentially to the selected image areas. The measurements were exported and further analyzed in R (v4.4.1).

### ORO staining

Neutral lipid content in lipid droplets was visualized by an ORO staining^16^. ORO powder (Sigma) was dissolved in 100% isopropanol at 0.5 g l^−1^ (stock). The working solution was prepared by mixing the ORO stock with water in a ratio of 5:4 (55.6% IPA with ORO, 44.4% H_2_O) and heating to 60 °C. Sections were taken from −80 °C, warmed up to room temperature, fixed in 4% PFA (15 min, room temperature) and subsequently washed in demineralized water and isopropanol/water (in a ratio of 5:4) before incubating the sections for 15 min in ORO working solution at 60 °C. Then, sections were washed in isopropanol/water (5:4) before further washing in demineralized water. Hematoxylin staining was performed as described above, and the sections were then washed in PBS and mounted in Mowiol 4-88 mounting medium.

### *MGLL* and *CD68* ISH

Duplex in ISH was performed using the Ventana Discovery Ultra automated staining platform (Roche Diagnostics), following the manufacturer’s standard protocol for RNA detection. Frozen sections were postfixed with 4% PFA and treated with Cell Conditioning 1 (Roche, 950-124) for target retrieval. RNAscope probes targeting *MGLL* and *CD68* mRNA (Advanced Cell Diagnostics, Bio-Techne) were hybridized at 43 °C for 2 h using the Discovery Ultra’s onboard protocols. Signal amplification and detection were performed using duplex chromogenic labeling, with MGLL visualized in green and CD68 in red using proprietary chromogenic substrates provided in the RNAscope Duplex Detection Kit. All reagents and amplification steps were integrated into the Ventana automated workflow. Slides were counterstained with hematoxylin, dehydrated in graded alcohols, cleared in xylene and mounted using VectaMount (Vector Laboratories). Whole-slide brightfield images were acquired at ×40 magnification using a Hamamatsu NanoZoomer digital slide scanner. Quantitative analysis of ISH signals was performed using the HALO image analysis platform (Indica Labs), applying the RNAscope ISH algorithm module. ROIs were manually annotated to define specific areas based on adjacent myelin and microglia stainings (HLA/PLP). The algorithm was configured to detect and quantify red (*CD68*) and green (*MGLL*) chromogenic puncta per cell. Positive cells were defined based on a minimum threshold of signal dots per nucleus, with settings optimized for signal-to-noise ratio and validated across control and experimental samples. Data were exported as the number of positive cells per mm^2^.

### MAGL activity-based histology using LEI-463-Cy5

The activity-based histology procedure was performed as detailed in ref. ^64^. In short, tissue was freshly sectioned at 10 µm and fixed in 4% PFA in PBS for 10 min at room temperature. Then, the sections were incubated with 1 µM LEI-463-Cy5 in 1% BSA, 20 mM HEPES buffer (pH 7.2). Afterward, the tissue was serially washed in PBS, water, 50% THF/H_2_O, water and finally in PBS. Subsequently, the sections were stained with antibodies as described for immunofluorescence stainings above, without Sudan Black quenching of autofluorescence. Autofluorescence was captured in an empty channel and used to control the other channels, as described in ref. ^64^.

### Proteomics sample preparation

Lysates were prepared from tissues by glass-bead mechanical lysis in 20 mM HEPES, 1 mM MgCl_2_, 2 U ml^−1^ benzonase. After protein quantification using the Bradford assay (Bio-Rad), lysates were snap-frozen in LN_2_ and stored at −80 °C until further use. Three micrograms of protein were resuspended in 20 µl of sodium deoxycholate (SDC) buffer (2% SDC, 10 mM Tris(2-carboxyethyl) phosphine, 10 mM Tris (pH 8.5), 40 mM chloroacetamide) supplemented with complete mini ethylenediaminetetraacetic acid-free protease inhibitor cocktail (Roche). The samples were denatured at 95 °C for 5 min. Cooled, reduced and alkylated samples were mixed with 162 µl of 50 mM ammonium bicarbonate, followed by the addition of trypsin (Promega) and LysC (Wako) in 1:50 and 1:75 ratios, respectively. The digestion took place overnight at 37 °C. The digestion was quenched with formic acid (FA) at the final concentration of 2% and centrifuged at 20,000*g* for 20 min to remove the SDC. The acidified supernatant containing 800 ng of peptides was loaded onto the EvoSep StageTips (EV2018) according to the manufacturer’s protocol.

### LC–MS/MS analysis proteomics

The samples were analyzed on EvoSep One liquid chromatography system (EvoSep) coupled to Exploris 480 mass spectrometer (Thermo Fisher Scientific). The peptides were eluted from the EvoSep StageTips and separated on an EvoSep analytical column (15 cm × 150 µm, 1.9 µm; EV-1106) using a 44-min gradient (30 SPD program) and the data were acquired in data-independent mode. The following mass spectrometric parameters were used for the full MS scan: scan mass range set to 375–1,100 *m*/*z*, resolution of 60,000 at 200 *m*/*z*, AGC target set to standard, maximum injection time set to auto. For the MS/MS spectra, the following parameters were used: quadrupole isolation window of 15 Da with 1 Da overlap between the windows (total of 40 windows), the precursor mass range was set to 400–1,000 *m*/*z*, resolution of 15,000 at 200 *m*/*z*, normalized AGC target was set to 1000%, injection time set to auto, normalized collisional energy was set to 27% and isotope exclusion set to on (or activated).

### Raw data processing proteomics

All raw files were processed with DIA-NN software (v1.8.1) with the deep learning in silico spectral library generation option. The digestion was set to trypsin with one missed cleavage allowed. Cysteine carbamidomethylation was set as a fixed modification and methionine oxidation was set as a variable modification. The threshold FDR for the protein identification was set to 1%. The full-scan mass accuracy was 6 ppm, and the optimized MS/MS mass accuracy was set to 22 ppm. All other settings were set to default. The UniProt human database, with 20,398 entries, was used for the search (released in April 2023). The quantification was based on the unique peptides for the downstream analysis. Two samples were excluded from the downstream analysis as they contained a very high percentage of missing values.

All the downstream analyses were performed in R (v4.4.1). Proteins that had a label-free quantification (LFQ) value in at least 70% (found, total ≥ 0.7) of samples in one group (lesion type) were included, and immunoglobulin variable regions were excluded. This led to 3,237 proteins included in downstream analysis. Missing values were imputed by sampling from a normal distribution around the lowest value found for a given protein, with an s.d. of one-third of the original s.d. The imputed dataset was used only for principal component analysis (PCA), while all other analyses were performed on the original dataset without imputation.

### Cytokine profiling

Cytokine concentrations were measured using a custom-made Human Luminex Discovery Assay (LXSAHM-26) according to the manufacturer’s protocol. Lysates from tissue samples were prepared as described for proteomics. Twenty-five microliters of 1 mg ml^−1^ lysate were thawed and diluted 1:2 in calibrator diluent buffer (RD6-52). The standard cocktails were reconstituted in RD6-52 buffer and further diluted 1:3 following the manufacturer’s instructions. In total, 50 µl of sample or standard was mixed with 50 µl of diluted microparticles cocktail and incubated for 2 h at room temperature on a horizontal orbital microplate shaker operating at 800 rpm. After the incubation, the plate was washed thrice with a magnetic plate washer, using 150 µl of wash buffer and allowing 1 min before removing the liquid. Next, 50 µl of diluted Biotin-Antibody Cocktail was added to each well and the plate was incubated for 1 h at room temperature while shaking at 800 rpm, followed by another washing step and the addition of 50 µl of diluted Streptavidin-PE. After 30-min incubation and a washing step, the microparticles were resuspended in 100 µl of wash buffer. The plate was analyzed with FLEXMAP 3D using Standard PTM settings and doublet discriminator gates set at 8,000 and 16,500. After assigning the microparticle region for each measured analyte, 50 µl of the samples was acquired. Cytokine concentrations were quantified by the instrument software (BioPlex Manager) using a five-parameter logistic curve.

### RNA extraction

Isolated lesions were dissolved in 500 µl Trisure (Bioline, BIO-38032). Subsequently, 100 µl CHCl_3_ was added to the tube and the solution was centrifuged (15 min, 11,000 rcf, 4 °C). The aqueous layer was carefully removed and 500 µl were added to the same tube for an additional extraction. After combining the two aqueous layers, 1 equal volume 70% EtOH was added and the solution was added to an RNAeasy column (QIAgen). The RNA was washed with RW1 buffer, after which any DNA contamination was removed by incubation with DNase (15 min, room temperature). After this, the column was washed in subsequent steps with RW1 buffer, then with RPE buffer and finally with 80% EtOH. The column was air dried for 5 min before eluting the RNA in 16-µl RNAse-free water. One microliter of the extracted RNA was used for analysis on a Denovix DS-11 spectrofotometer to analyze the purity and concentration.

### RNA-seq and analysis

Isolated RNA was sent for total RNA-seq at Genomescan (Leiden). RNA integrity was assessed, delivering an RNA quality (RQN/RIN) value, which is used in downstream analysis as a covariate. No minimum RQN value was set as a requirement. After library prep, a library quality control (QC) was performed, which gave satisfactory results. Subsequently, the libraries were sequenced on a NovaSeq 6000 sequencer for a target library size of 30 million reads. Alignment was performed with HISAT (v2.2.0) against the human Ensembl GRCh38 reference genome. Ensembl identifiers were converted into gene names using AnnotationDbi and the human reference genome database Org.Hs.eg.db (v3.16.0) from Bioconductor.

The raw count data were subsequently loaded in R (v4.4.1) and a data analysis pipeline was conducted using the edgeR, voom and limma packages in R^96,97,99^. Detailed filtering criteria and analysis choices are specified in Supplementary Methods. Methods for weighted gene-coexpression network analysis (WGCNA)^39^, single-cell deconvolution and gene set enrichment can be found in Supplementary Methods.

### PCA

PCA was performed using the ‘prcomp’ function in base R. ‘Scale’ was set to FALSE, to preserve highly variant variables to have more weight in the PCA. PCA was performed on the full proteomics, lipidomics and ABPP datasets. For RNA-seq, PCA was performed on the 1,000 most variable genes. The obtained principal components were rotated to focus loadings of variables onto a single component using the ‘varimax’ function. Correlation of principal components to metadata was performed using the PCAtools package, using the function ‘eigencorplot’. The number of components was determined using the elbow criterion, in PCAtools automated in the function ‘getelbow’.

### MOFA

MOFA was performed based on the workflow discussed in ref. ^53^, using MOFA2 (v1.8.0). Data input for MOFA was the full lipidomics dataset (712 lipids) and a reduced RNA-seq and proteomics dataset. Only highly variable transcripts were selected by filtering for genes with an s.d. higher than 2× mean(s.d.) of the full dataset, which yielded 1,032 transcripts. Proteins were selected by filtering for proteins with an s.d. higher than 1× mean(s.d.) of the full dataset, which yielded 1,218 proteins. A total of 92 samples had data for all three data modalities. A total of 11 samples had data for two data modalities and 7 samples had data from only one data modality (Supplementary Fig. 10a).

We ran the MOFA model with largely default settings. Scale_views was set to ‘TRUE’, convergence mode was ‘medium’ and maximum iterations was 2,000. We obtained seven factors that explained a minimum of 5% variance in at least one data modality. The total variance explained by seven factors were 53.5% for the RNA-seq input, 57.7% for the proteomics input and 72.8% for the lipidomics dataset. Factor values were tested for differential expression across groups using two-sided Wilcoxon rank-sum test using the rstatix package (v.0.7.2), with correction for multiple testing using BH FDR.

Dimensionality was further reduced using uniform manifold approximation and projection using the package uwot (v.0.1.16) using a n_neighbours parameter of 20. Pseudotime trajectories were calculated using Slingshot (v2.6.0) using clusters generated by MClust (v6.1) using a G parameter of 5. The starting cluster was defined as the cluster containing CWM samples. Generated trajectories were extracted using the ‘slingCurves’ function and plotted onto the uniform manifold approximation and projection using ‘geom_path’. Trajectories were plotted against the original input data (factor values) and a smooth fit was plotted using the ‘loess’ fitting function of ggplot2.

MOFA factor 3 was associated with the clinical features in a generalized estimating equation model, accounting for donor identity, because our samples were nested within donors (100 samples from 28 MS donors). To this end, we used the function ‘geeglm’ from the package geepack (v1.3.10), with a Gaussian distribution and an exchangeable correlation structure.

### Lipidomics sample preparation

Samples (50-µm sections) were lysed, and protein concentrations (Bradford method) were quantified. Two study QC pools (normal WM pool and lesion pool) were prepared using 10–100 μl of more than 50% of all the samples. A total of 11 QC aliquots were made separately from the normal WM pool and lesion pool.

Purchased or synthesized standards, internal standards (IS) were dissolved in methanol, ethanol, chloroform or ACN. These stock solutions were further diluted and mixed to make the standard stock solutions and IS stock solutions. The eCB synthesis IS mix contained 18:1, 18:1-PE-N-17:0, p18:1, 18:1-PE-N-17:0, 18:1-OH-PE-N-17:0, p18:1-OH-PE-N-17:0, OH-OH-PE-N-17:0. The lipids IS mix contained deuterated version of ceramides, (lyso)phospholipids, diacylglycerols, triacylglycerols and CEs. The signaling IS mix contained deuterated version of oxylipins, eCBs, free fatty acids and bile acids. The eCB synthesis calibration standards contained standards of precursors of eCBs; the signaling calibration standards contained oxylipins, eCBs, free fatty acids and bile acids; and the oxidized lipids calibration standards contained oxidized versions of phosphatidylcholines. The IS mixes and calibration standards were stored at −80 °C. The mixed IS working solutions were prepared and stored at −20 °C till further use.

Aliquots of sample lysates (~1 mg protein per ml, 50 μl) were thawed on ice. To each sample, 10-μl IS work solution was added. Calibration samples were prepared by spiking 10 μl of each calibration standard into 50 μl of water. Extraction was performed by 100-μl extraction buffer (0.2 mM ammonium formate) and 1,000-μl extractant (MTBE:BuOH, 50:50, vol/vol). Samples were then mixed in a Next Advance Bullet Blender (5 min, 90% speed, room temperature), followed by centrifugation (16,000*g*, 10 min, 4 °C). In total, 950 μl of the organic layer was transferred into clean, precooled tubes and concentrated in a SpeedVac vacuum concentrator (Thermo Fisher Scientific), followed by adding 50 µl of reconstitution solution (MeOH:ACN, 30:70, vol/vol) and agitating for 15 min. The reconstituted samples were centrifuged (16,000*g*, 10 min, 4 °C) and 40 µl were transferred into autosampler vials with inserts. Samples were kept at −80 °C till LC–MS analysis.

Samples were randomized and run in one batch. Each batch included QC samples and blank samples. QC samples are used to assess data quality. Method blanks (proc blanks) are used to check for background signal. These samples are composed of analyte-free matrix and have undergone all steps of the sample preparation procedure using only reagents. The detailed LC/MS–MS procedures for all lipidomics platforms are specified in the Supplementary Methods.

### Chemical proteomics (ABPP) sample preparation

The chemical proteomics workflow was based on the previously reported procedures discussed in ref. ^57^. Lysates were prepared by glass-bead mechanical lysis of isolated lesions in 20 mM HEPES, 1 mM MgCl_2_, 2 U ml^−1^ benzonase. After protein quantification using the Bradford method (Bio-Rad), lysates were snap-frozen in LN_2_ and stored at −80 °C until further use.

Lysates (100 µl, 1 mg ml^−1^ protein) were thawed on ice and subsequently treated with a cocktail of 10 µM FP-biotin and 10 µM THL-biotin as described previously^57^. As controls, ten heat and SDS-inactivated (1% SDS, 5 min, 95 °C) lysates were taken along in the procedure. Probe-treated lysates were precipitated using the MeOH/CHCl_3_ precipitation. The protein pellet was washed in MeOH and subsequently redissolved in PBS containing 0.5% SDS and 5 mM DTT. Proteins were dissolved by sonication and the solution was incubated at 65 °C for 15 min to reduce cysteines. Afterward, cysteines were alkylated using iodoacetamide and excess iodoacetamide was quenched using DTT. Subsequently, the samples were added to a suspension of 5-µl high-capacity streptavidin agarose beads (Thermo Fisher Scientific, 20361) and 15-µl control agarose beads in PBS (Thermo Fisher Scientific, 26150) containing 0.25% SDS. The suspension was incubated for 2 h at room temperature while rotating head-overhead to let probe proteins bind to the beads. After incubation, the beads were washed with PBS containing 0.5% SDS (4×) and subsequently with PBS (5×). On-bead digestion was performed with 0.25 µg sequence-grade trypsin (Promega) in 100 mM Tris, 100 mM NaCl, 1 mM CaCl_2_ and 2% (vol/vol) acetonitrile overnight at 37 °C while shaking at 1,000 rpm. Trypsin was quenched with 10 µl FA. Peptides were then desalted on Oasis C18 plates (Waters) and eluted in 60% acetonitrile with 0.5% FA. The solvent was evaporated in a SpeedVac (45 °C, Eppendorf), and samples were stored at −80 °C until further use.

### LC–MS/MS for chemical proteomics (ABPP)

Samples were randomized and assigned to six blocks, each containing 23 samples. Each block also contained four reference samples that were measured in every measurement block as described in ref. ^100^. Per block of samples, peptides were then reconstituted in 3% acetonitrile, 0.1% FA in H_2_O and analyzed on a QExactive HF LC–MS/MS (Thermo Fisher Scientific). All gradients and solutions were prepared according to previously published procedures^57^. Before and after each block, the LC/MS machine was cleaned, recalibrated and its performance was assessed.

### Data analysis ABPP with chemical proteomics

Raw spectral data from the chemical proteomics experiments were analyzed using MaxQuant (v2.0.1.0) using largely default settings^101^. Match across runs was enabled. The ‘proteingroups.txt’ output file from MaxQuant was imported in R. Identified proteins were filtered for potential contaminants identified by MaxQuant, and additional criteria for the proteins were that (1) a protein was identified with two unique peptides, (2) the ratio of protein raw intensity of native, heat inactivated lysate was at least 2.0 in at least five of ten QC pairs, (3) the protein is annotated as a serine hydrolase, or has a annotation in UniProt as ‘charge-relay system’ or ‘nucleophile’ as a catalytic residue and (4) the protein has an LFQ value for more than 60% samples in at least one biological group (lesion type). A total of 97 enzymes met these criteria and were further processed.

The LFQ values were then corrected for LC/MS performance by block design following the procedure described in ref. ^100^. In short, the median log_2_ LFQ for a protein in the reference samples was compared to the same samples in other blocks. The difference in log_2_ median was subtracted from the same protein in the experimental samples. Analysis of the reference samples themselves showed that this correction improved the Pearson’s correlations of the reference samples to each other (data not shown). All subsequent analyses were then performed using the corrected log_2_-transformed LFQ values.

Missing values were imputed using the random Gaussian distribution centered around the minimum LFQ value found for that protein in the dataset, with an s.d. equal to one-third of the s.d. of that protein in the dataset. This is based on the assumption that, if a protein was not found, it was most likely below the detection threshold, and therefore very low.

Enzyme activity values were associated with the MOFA factors derived from the other datasets using limma. The ABPP dataset was log_2_-transformed, centered around 0 and then fed into a linear model using the design *y* ~ f1 + f2 + f3 + f4 + f5 + f6 + f7. *P* values for the derived coefficients were calculated using the eBayes function, and subsequently adjusted for multiple testing using the BH method with an FDR of 10%.

### CSF lipidomics

Three hundred microliters of fresh-frozen CSF were spiked with 10-µl IS calibration standard, and subsequently extracted using the same method as described for the lesions. For the CSF lipids, only the signaling lipids platform was measured, which includes the oxylipins and prostaglandins.

Integrated MS/MS peaks (response ratio) were log_2_-transformed, centered around 0, and missing values were not imputed. These response ratio values were correlated to the proportion of foamy lesions in the WM of the originating donor. Given the non-normal distribution of the proportions (between 0 and 1), the Spearman correlation was used. Then, to calculate if there were significant associations, the CSF oxylipin levels were modeled in a quasibinomial distributed generalized linear model using the counts of lesions with foamy microglia against other lesion types, as described for the clinical correlations presented in Fig. 1g,h. *P* values were determined using the likelihood-ratio test, and, because this was an exploratory analysis of oxylipins in the CSF, we did not correct for multiple testing.

### LPC spinal cord injection in mice

#### Animals

Adult male and female C57BL/6JRj mice (14 weeks old; 20–30 g) were used in this study and obtained from Janvier Labs (France). Mice were housed under a 12-h light/12-h dark cycle with ad libitum access to food and water. All animal experiments were conducted in accordance with the Swiss Federal Act on Animal Protection and were approved by the Cantonal Veterinary Office Basel-Stadt (license 3095).

#### Anesthesia

Animals received a subcutaneous injection of buprenorphine (0.2 mg kg^−1^) for 45 min to 2 h before surgery for pre-emptive analgesia. Anesthesia was induced with ~3.5% isoflurane and maintained at ~2% isoflurane through a face mask. Throughout the procedure, oxygen saturation, pulse and body temperature were continuously monitored.

### Surgical procedure

Mice were placed on a heated surgical platform and the dorsal fur was shaved and disinfected with Betadine. Local anesthesia (0.1 ml of 0.1% lidocaine and 0.025% bupivacaine) was administered subcutaneously at the incision site. A midline skin incision (~2 to 3 cm) was made above the thoracic spine. Paraspinal muscles were carefully retracted to expose the vertebral column. The intervertebral space between T10 and T12 was identified and cleaned of overlying muscle. The animal was stabilized by gently clamping the tissue anterior to the injection site with a hemostat. The meninges were pierced using a 30-G needle adjacent to the central dorsal vessel. A Hamilton syringe fitted with a custom-pulled glass capillary was inserted at a 65 ° angle, targeting the ventral WM. A total of 1 μl of 1% LPC (L-α-lysophosphatidylcholine; Sigma, L4129) dissolved in 0.9% NaCl was injected in two steps—0.5 μl, followed by a 0.5 mm retraction of the syringe and injection of an additional 0.5 μl. After a 1-min delay, the syringe was slowly withdrawn. The paraspinal muscles were sutured using 6-0 Prolene (C-1), and the skin was closed with sutures.

### Postoperative care

Animals received meloxicam (5 mg kg^−1^, subcutaneous) immediately after surgery and daily for the following 2 days. They were housed in a warmed recovery cabinet until fully awake. Postoperative monitoring included daily scoring for 3 days (and weekly thereafter), with close attention to body weight, wound healing and hindlimb motor function. Weight loss was limited to ≤10% and typically recovered within 2–3 days.

### RO7232432 treatment

Mice received daily intraperitoneal injections of RO7232432 (MAGLi-432) at 5 mg kg^−1^ (10 ml kg^−1^ dosing volume) starting from the second day after LPC injection. The treatment continued once daily until the designated experimental endpoint. Doses were prepared freshly in vehicle solution and administered at the same time each day to minimize variability. Control groups received vehicle only.

### Reporting summary

Further information on research design is available in the Nature Portfolio Reporting Summary linked to this article.

## Online content

Any methods, additional references, Nature Portfolio reporting summaries, source data, extended data, supplementary information, acknowledgements, peer review information; details of author contributions and competing interests; and statements of data and code availability are available at 10.1038/s41593-026-02302-3.

## Supplementary information

Supplementary Information Supplementary Figs. 1–15 and Supplementary Methods.

Reporting Summary

Supplementary Tables Supplementary Tables 1–4.

## Source data

Source Data Fig. 2 Statistical source data for the ORO quantification in Fig. 2j.

Source Data Fig. 3 Statistical source data for the TBXAS1, PLIN2 and LAMP1 quantification in Fig. 3k,n,o.

Source Data Fig. 4 Statistical source data for the GPNMB/HLA quantification in Fig. 4l.

Source Data Fig. 6 Statistical source data for the RNAscope analyses in Fig. 6h,i.

Source Data Extended Data Fig. 1 Statistical source data containing study metadata and quantitative microglia burden for Extended Data Fig. 1a,d.

Source Data Extended Data Fig. 10 Statistical source data for Extended Data Fig. 10c–h,j,l,n,p.

## Data Availability

RNA-seq data are available from the Gene Expression Omnibus database under accession GSE279972. Mass spectrometry data are available through the ProteomeXchange Consortium through PRIDE upon publication (proteomics—PXD056856 and ABPP—PXD056899). All processed data are also available on Zenodo (10.5281/zenodo.17735822)^102^.

## Code availability

R code supporting the analyses described in this paper is available on Zenodo (10.5281/zenodo.17735822)^102^.
