# Supplementary material for: Foamy microglia link oxylipins to disease progression in multiple sclerosis
Source: Nat Neurosci. 2026 May 21;29(7):1585–98. doi: 10.1038/s41593-026-02302-3 (PMC13337515; doi:10.1038/s41593-026-02302-3)
Supplement: Supplementary file 1 — Supplementary Figs. 1–15 and Supplementary Methods. [file 41593_2026_2302_MOESM1_ESM.pdf]

nature neuroscience

Supplementary information https://doi.org/10.1038/s41593-026-02302-3

Foamy microglia link oxylipins to disease progression in multiple sclerosis

In the format provided by the authors and unedited

Supplementary Figures

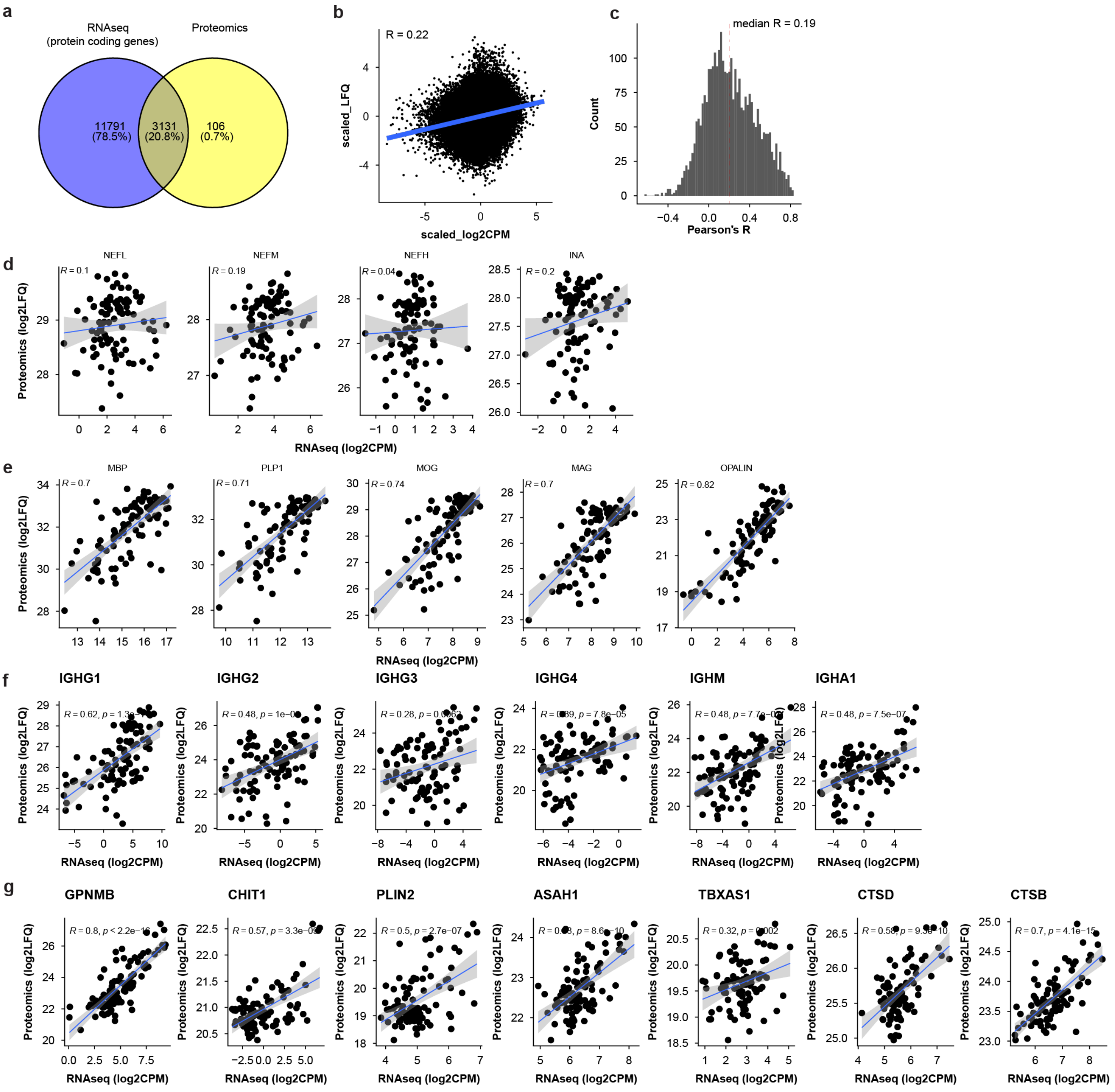

Supplementary Fig. 1 | RNA protein correlation in MS lesions. a, overlap between protein-coding genes detected by RNA sequencing or proteomics. **b,** global correlation of RNA and protein levels. All genes/proteins were z-scored and a Pearson correlation coefficient was calculated on the global level, with all genes/protein added together. **c,** correlations were calculated (Pearson) for Individual gene-protein pairs and the obtained R values were plotted in a histogram, showing that some genes have no correlation with their protein product, while other genes are strongly correlated. **d-g,** example correlations of proteins as calculated in **c** for proteins of the axon (**d**), myelin sheath (**e**), immunoglobulins (**f**) or proteins related to foamy microglia (**g**). Error bands in **d-g** represent the 95% confidence interval.

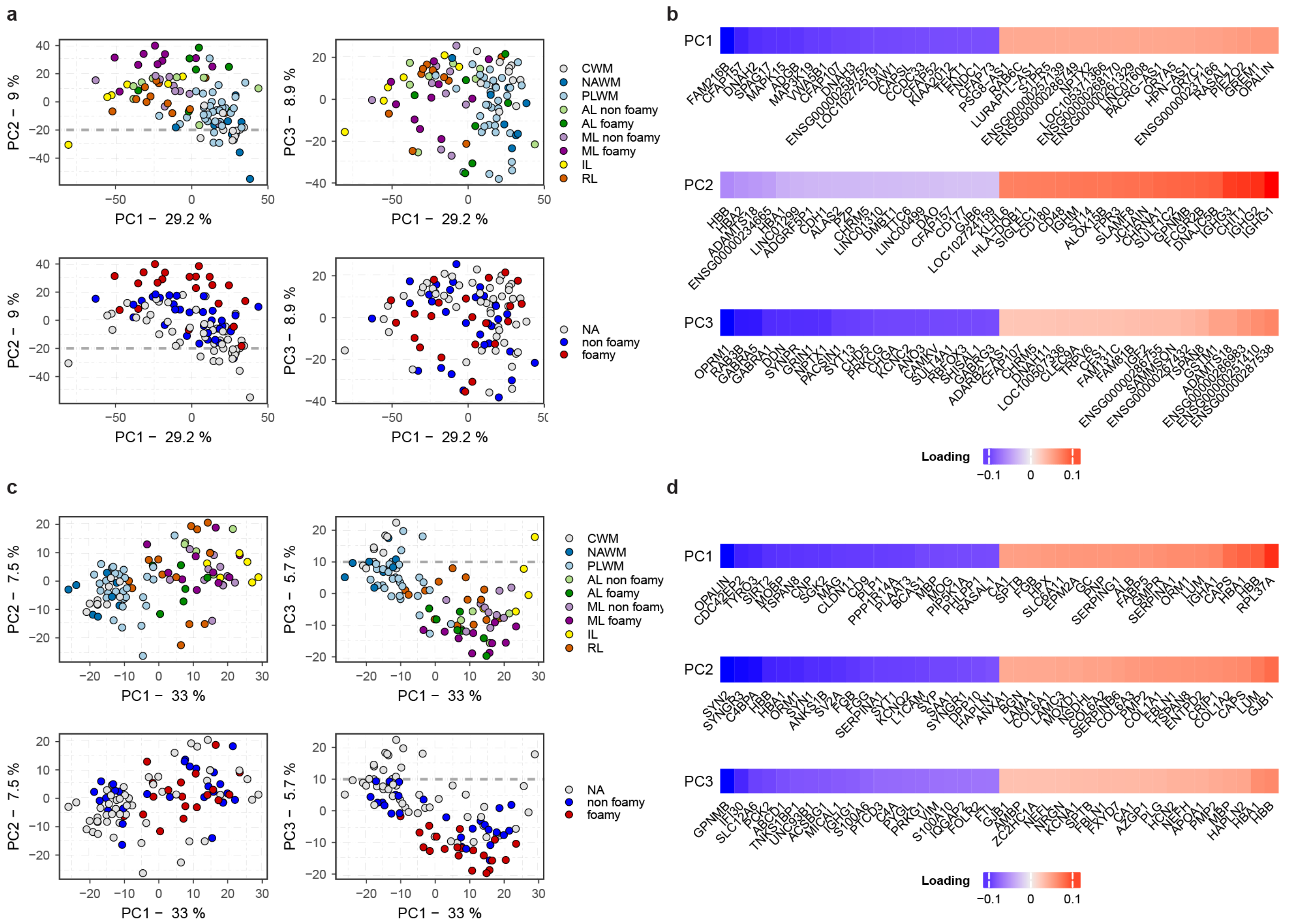

Supplementary Fig. 2 | PCA of the RNAseq and proteomics data sets. a+c, PCA plots showing the first 3 principal components for the RNAseq data (**a**) or the proteomics data (**c**), coloured by either lesion type or morphology. **b+d,** top 20 positive and negative loadings for the first three principal components for the RNAseq data (**b**), or the proteomics data (**d**).

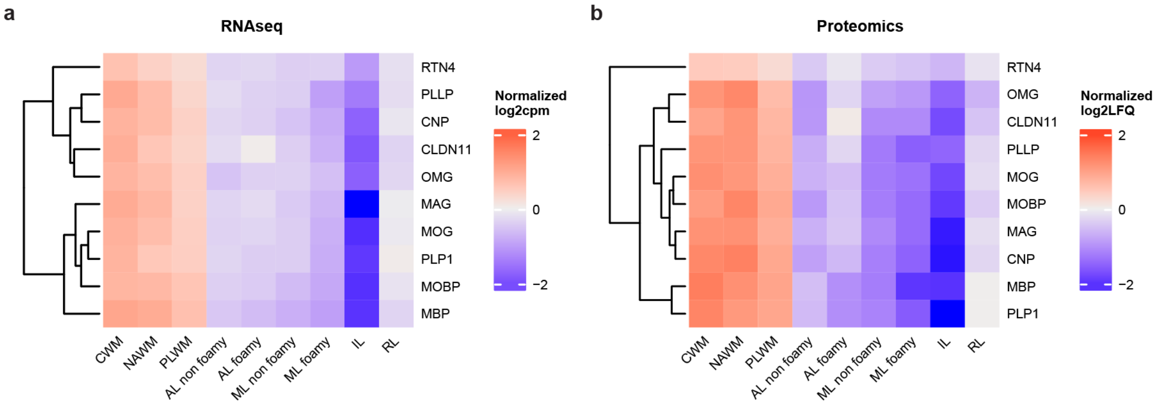

Supplementary Fig. 3 | Myelin protein levels decrease in all lesion types. a, Mean myelin gene expression (**a**) of protein levels (**b**) across lesion types. Data is expressed as the log2 normalized relative expression value: counts-per-million for **a** and label-free quantification for **b**.

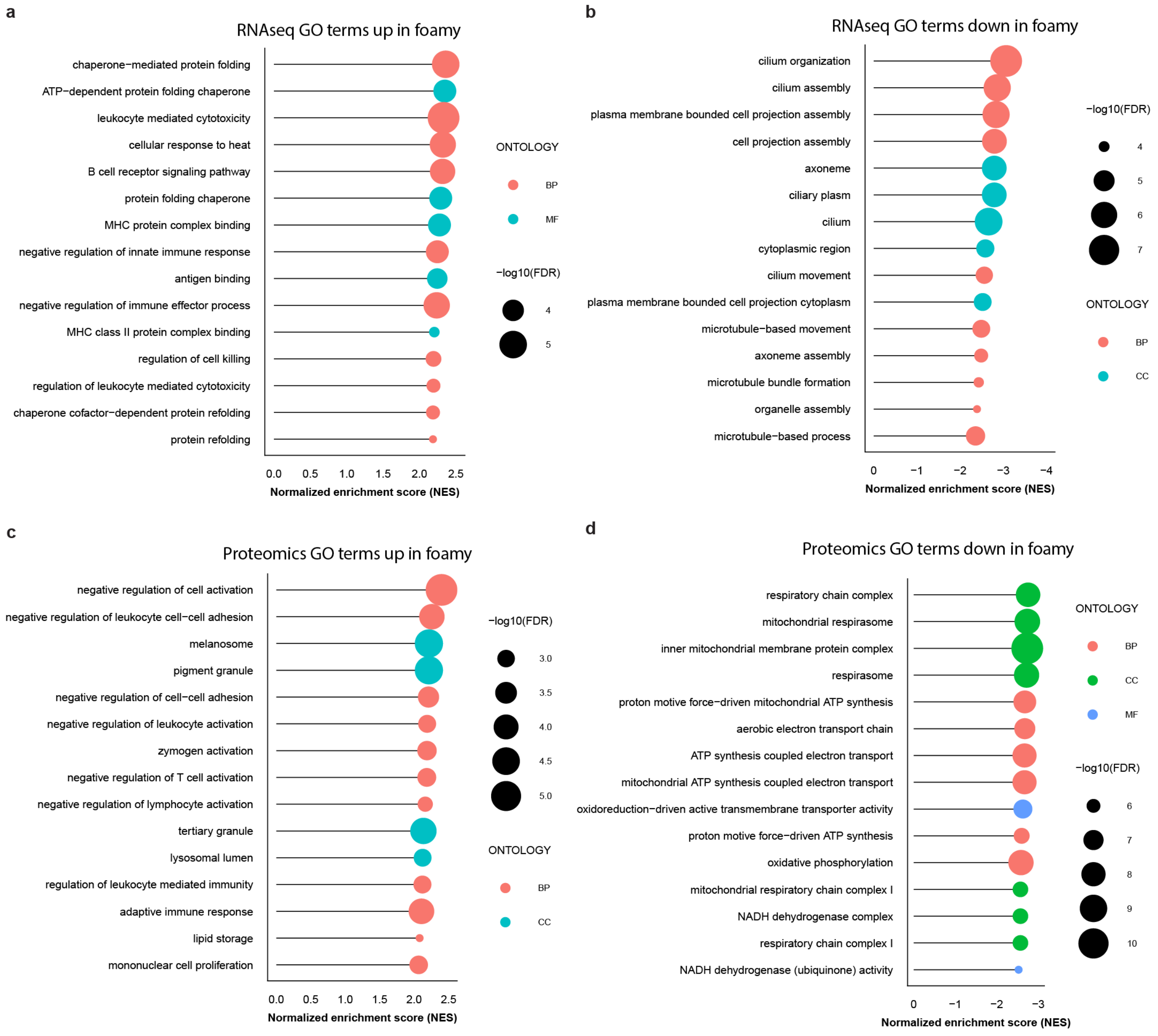

Supplementary Fig. 4 | Pathways associated with foamy microglia compared to non-foamy microglia. Top 15 pathways enriched in the differential expression analysis between lesions with foamy microglia versus non-foamy microglia of RNA sequencing data (a-b) and proteomics (c-d) using gene set enrichment analysis (GSEA). BP, biological process; CC, cellular compartment; MF, molecular function. P-values were calculated through the gseGO function of clusterprofiler with BH-correction for multiple testing.

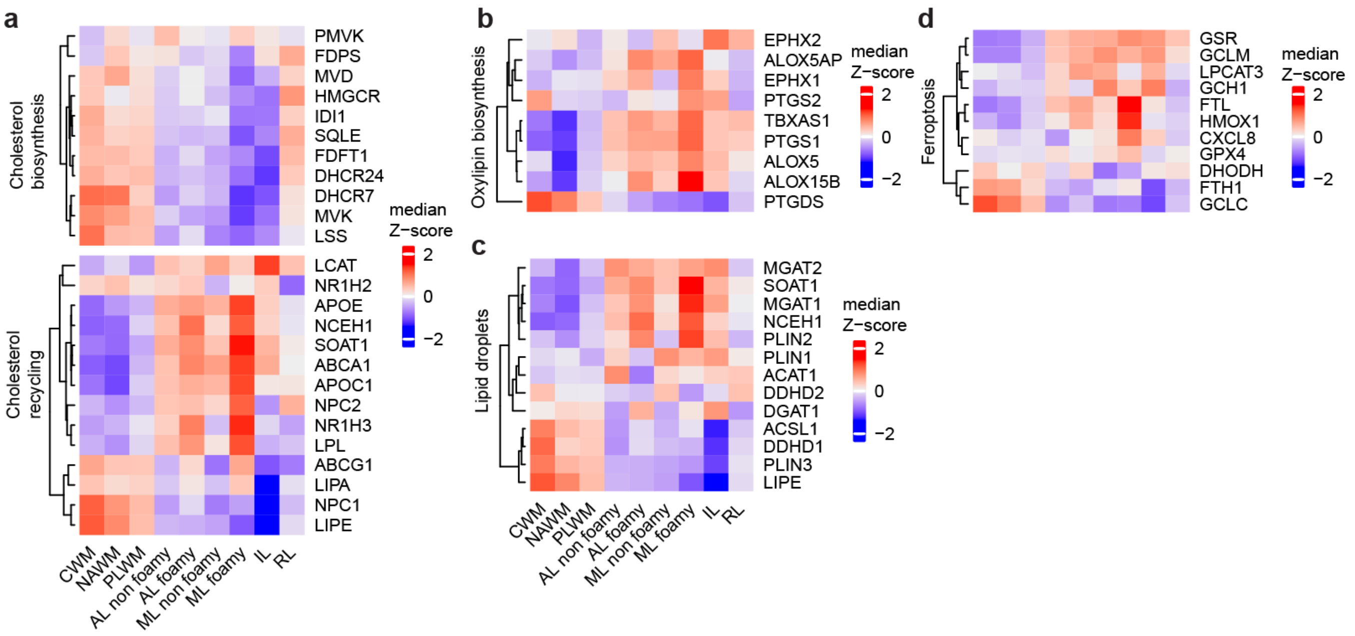

Supplementary Fig. 5 | Genes involved in lipid metabolism are increased in lesions, most strongly in lesions with foamy microglia. Heatmaps depicting Z-scores of gene expression of genes involved in cholesterol synthesis and recycling (a), oxylipin biosynthesis (b), lipid droplet formation (c) and ferroptosis and lipid peroxidation (d).

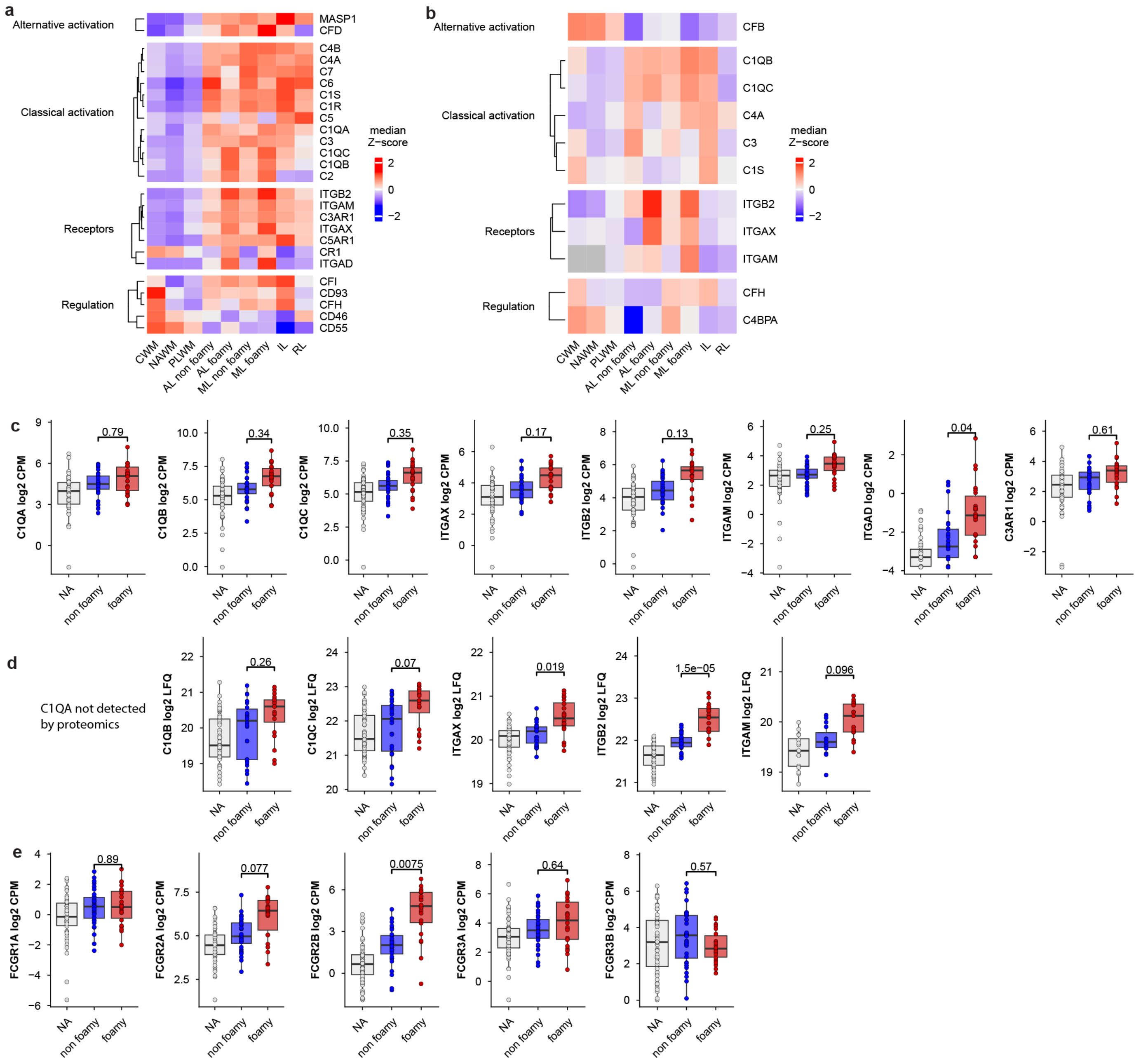

Supplementary Fig. 6 | Expression of antibody-mediated phagocytosis pathways. a-b, Heatmap depicting genes (**a**) or proteins (**b**) of the complement system. Data is expressed as the median Z-scored log2 counts per million (**a**) or LFQ (**b**) per lesion type. **c-d,** expression of C1Q genes , as well as iC3b receptors on the gene level (**c**) or protein level (**d**). **e**, Gene expression of Fc gamma receptors. For panels **c-e,** data is expressed as individual values (circles), the median as the centre, boxes representing the first and third quartile and whiskers extending to datapoints within the 1.5xinter-quartile range. Statistics are calculated using limma (two-sided) with BH-correction for multiple testing.

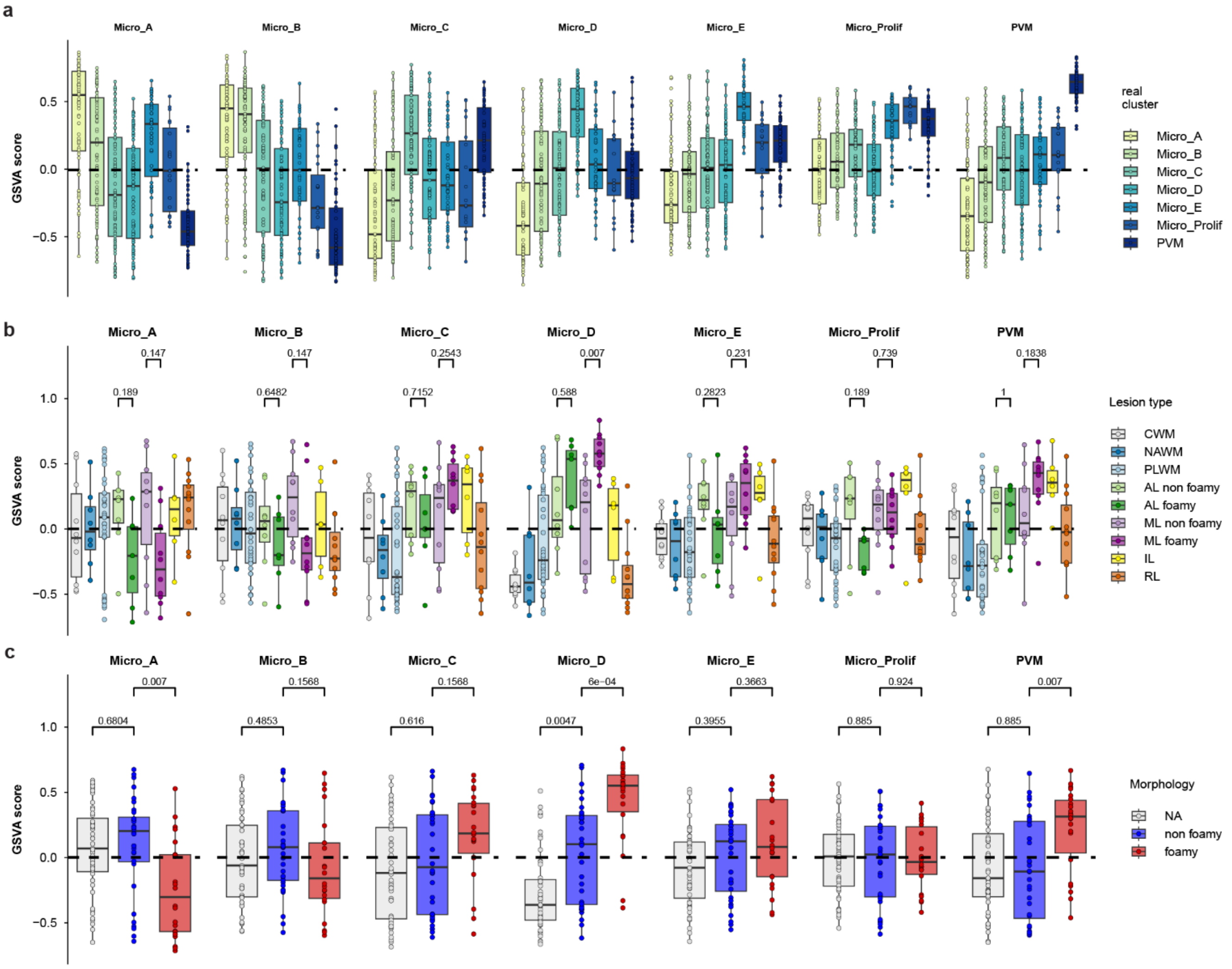

Supplementary Fig. 7 | The Micro_D state is enriched in foamy MS lesions. a, validation of the method. Marker genes for each microglial state enriched their own microglial state, except for micro_B. **b-c,** boxplots showing enrichment of each state using GSVA. Statistical test is a wilcoxon rank sum test with BH correction for multiple testing. Data is expressed as individual values (circles), the median as the centre, boxes representing the first and third quartile and whiskers extending to datapoints within the 1.5xinter-quartile range.

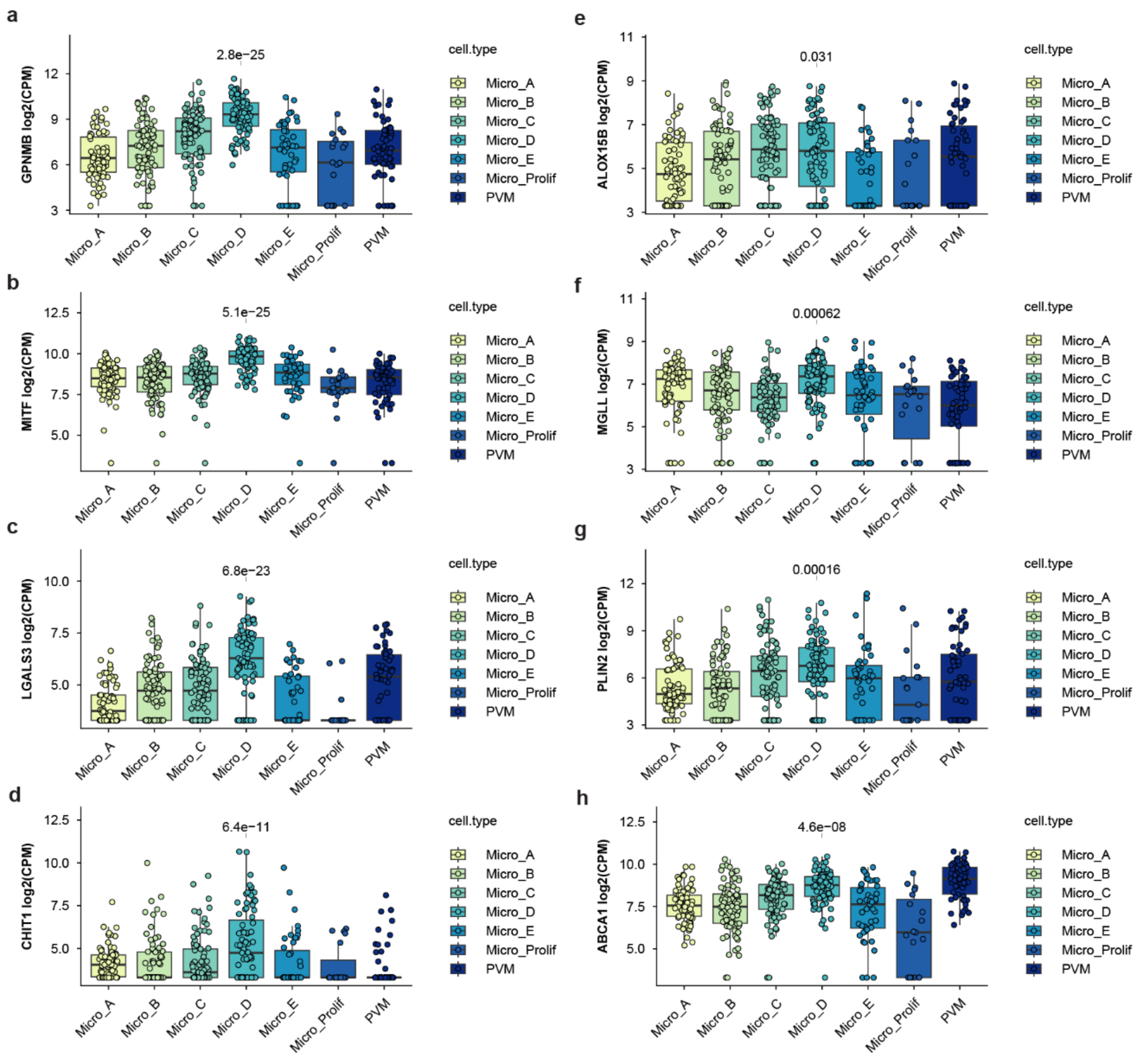

Supplementary Fig. 8 | Marker genes for the Micro_D microglia state. a-d, top marker genes for Micro_D. **e-h,** selected genes involved in lipid metabolism that are enriched in the Micro_D profile. P-values represents the comparison of Micro_D to the combined other profiles. P-values were calculated using Limma and BH-corrected for multiple testing. Data is expressed as individual values (circles), the median as the centre, boxes representing the first and third quartile and whiskers extending to datapoints within the 1.5xinter-quartile range.

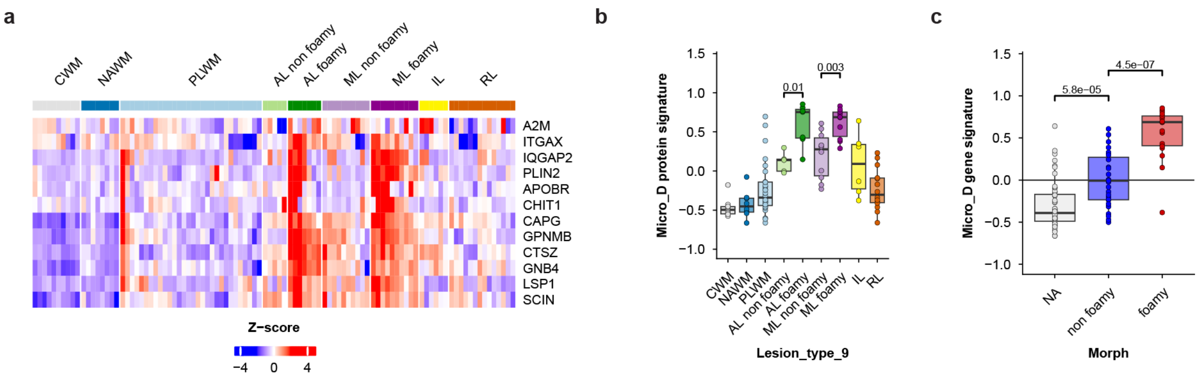

Supplementary Fig. 9 | Micro_D marker genes on the protein level. a, Protein Z-scores of proteins encoded by Micro_D marker genes shows strong upregulation in lesions with foamy microglia. **b-c,** The proteins from **a** were summarized into one protein signature score using gene set variation analysis (GSVA). Statistics represent p-values from two-sided Wilcoxon rank sum tests. For panels **b** and **c,** data is expressed as individual values (circles), the median as the centre, boxes representing the first and third quartile and whiskers extending to datapoints within the 1.5xinter-quartile range.

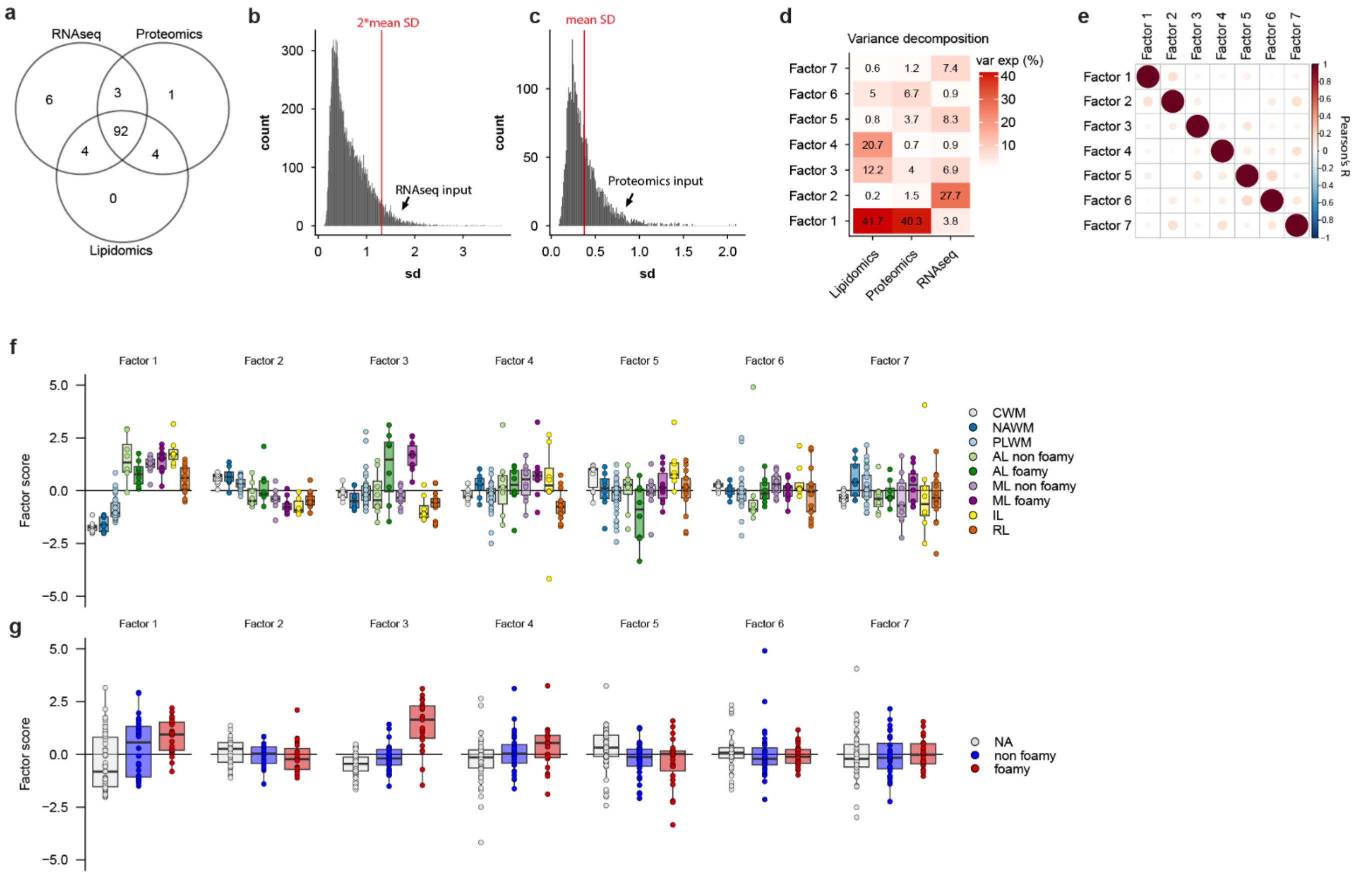

Supplementary Fig. 10 | Multi-omics data integration. a, overview of the sample-overlap between the different data modalities. Most samples have been analyzed by all three omics-technologies. However, for some samples there was not enough material, or the data was excluded because of insufficient data quality. **b-c,** data input for RNAseq and proteomics was restricted to highly variable genes (**b**) or proteins (**c**). **d,** variance decomposition of the seven derived MOFA factors. **e,** Pearson correlation of the MOFA factors shows factors are mostly orthogonal to each other. **f-g,** boxplots showing the distribution of MOFA factor scores across lesion type (**f**) or morphology (**g**). For panels **f** and **g**, data is expressed as individual values (circles), the median as the centre, boxes representing the first and third quartile and whiskers extending to datapoints within the 1.5xinter-quartile range.

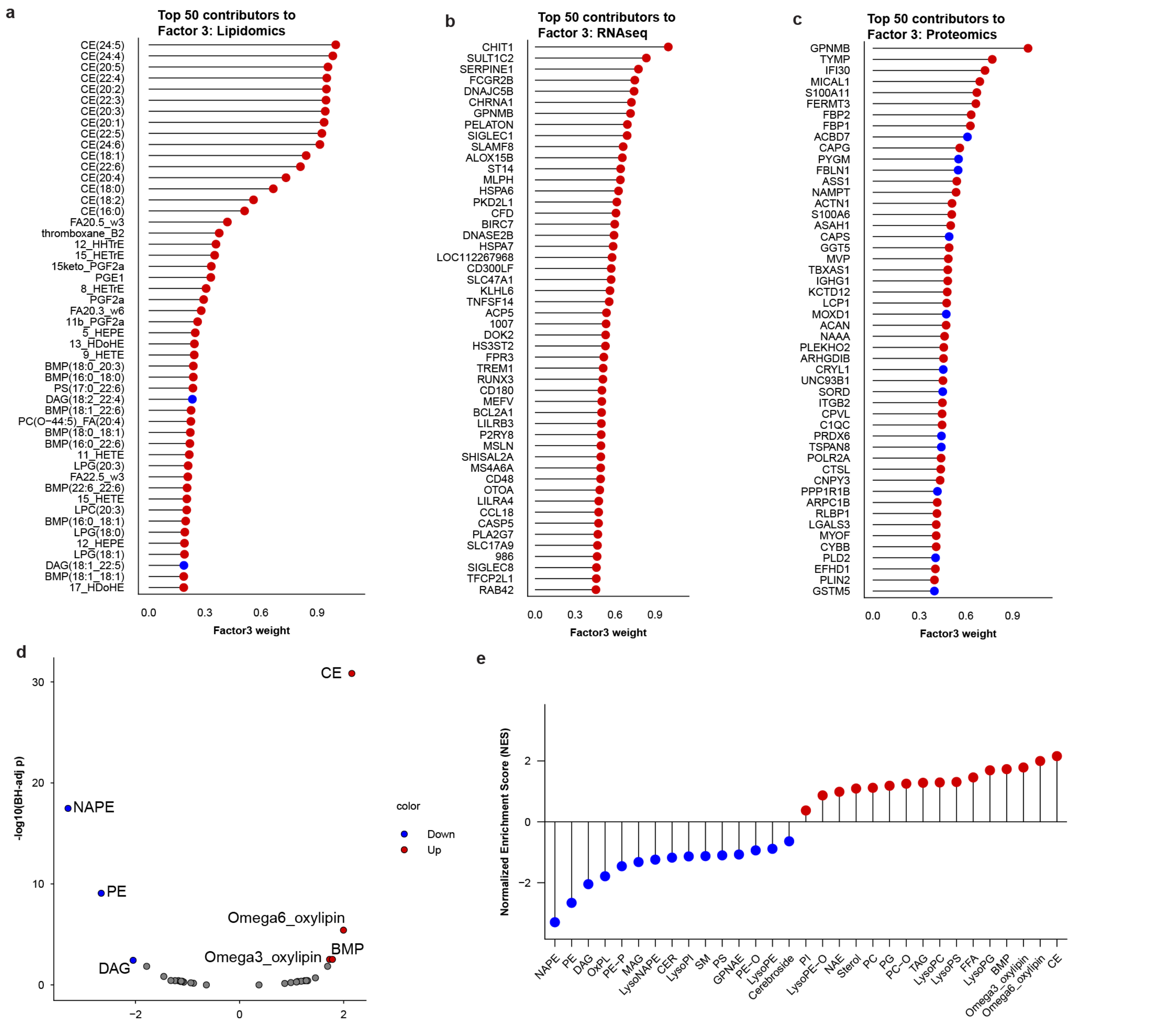

Figure S15

Supplementary Fig. 11 | Top loadings of MOFA factor 3. a-c, Top 50 lipid (**a**), gene (**b**) or protein (**c**) relative loadings of MOFA factor 3. Data is expressed as the relative loading while the color indicates directionality (red: positively associated with factor 3, blue: negatively associated with factor 3). **d-e,** enrichment of lipid classes enriched in factor 3 using GSEA with the lipid class as custom “genesets”. P-values and enrichment scores were calculated through the gseGO function of clusterprofiler with BH-correction for multiple testing.

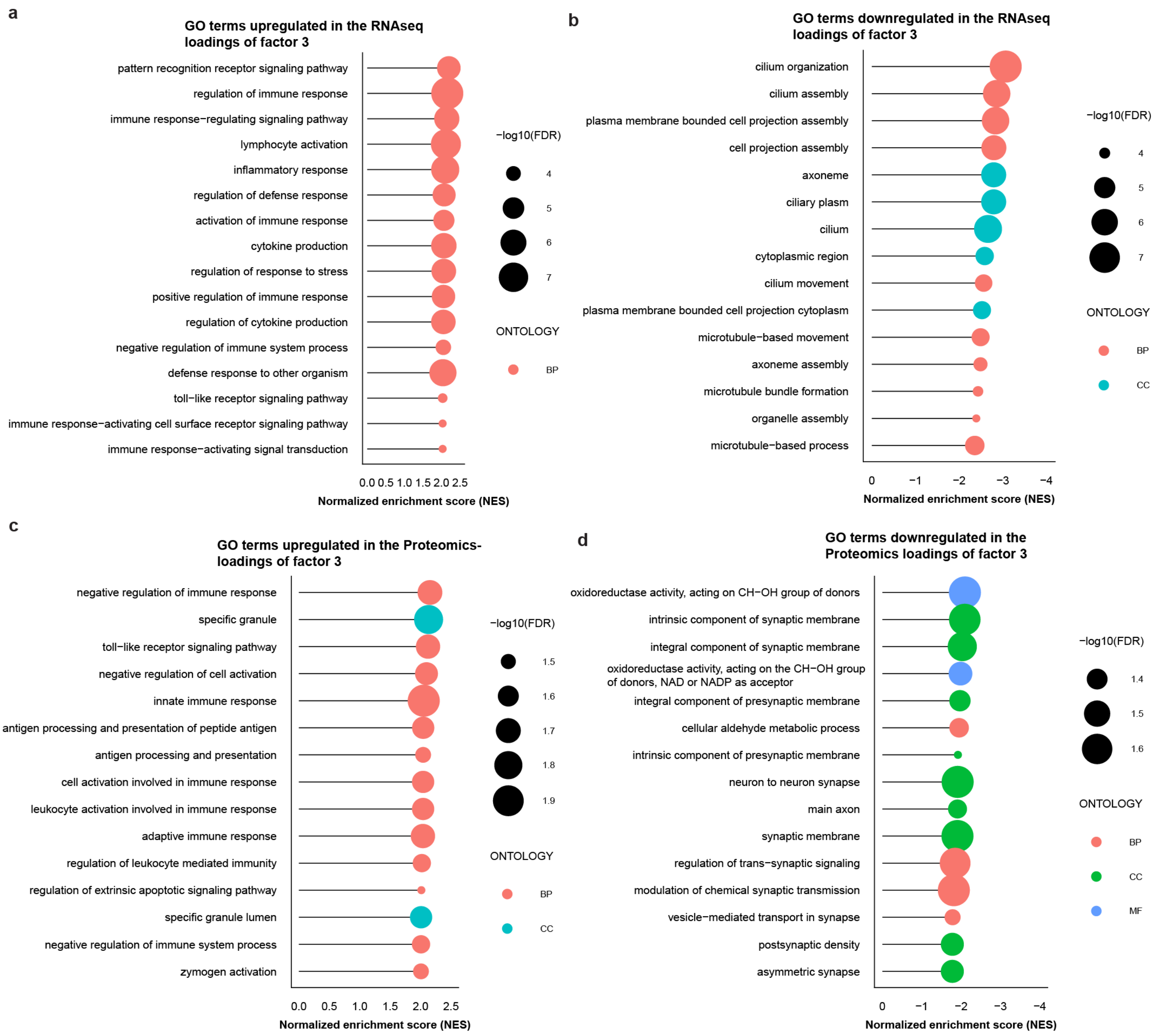

Supplementary Fig. 12 | Pathways associated with factor 3. Top 15 pathways enriched in factor 3 loadings for genes (a-b) and proteins (c-d) using gene set enrichment analysis (GSEA). BP, biological process; CC, cellular compartment; MF, molecular function. P-values were calculated through the gseGO function of clusterprofiler with BH-correction for multiple testing.

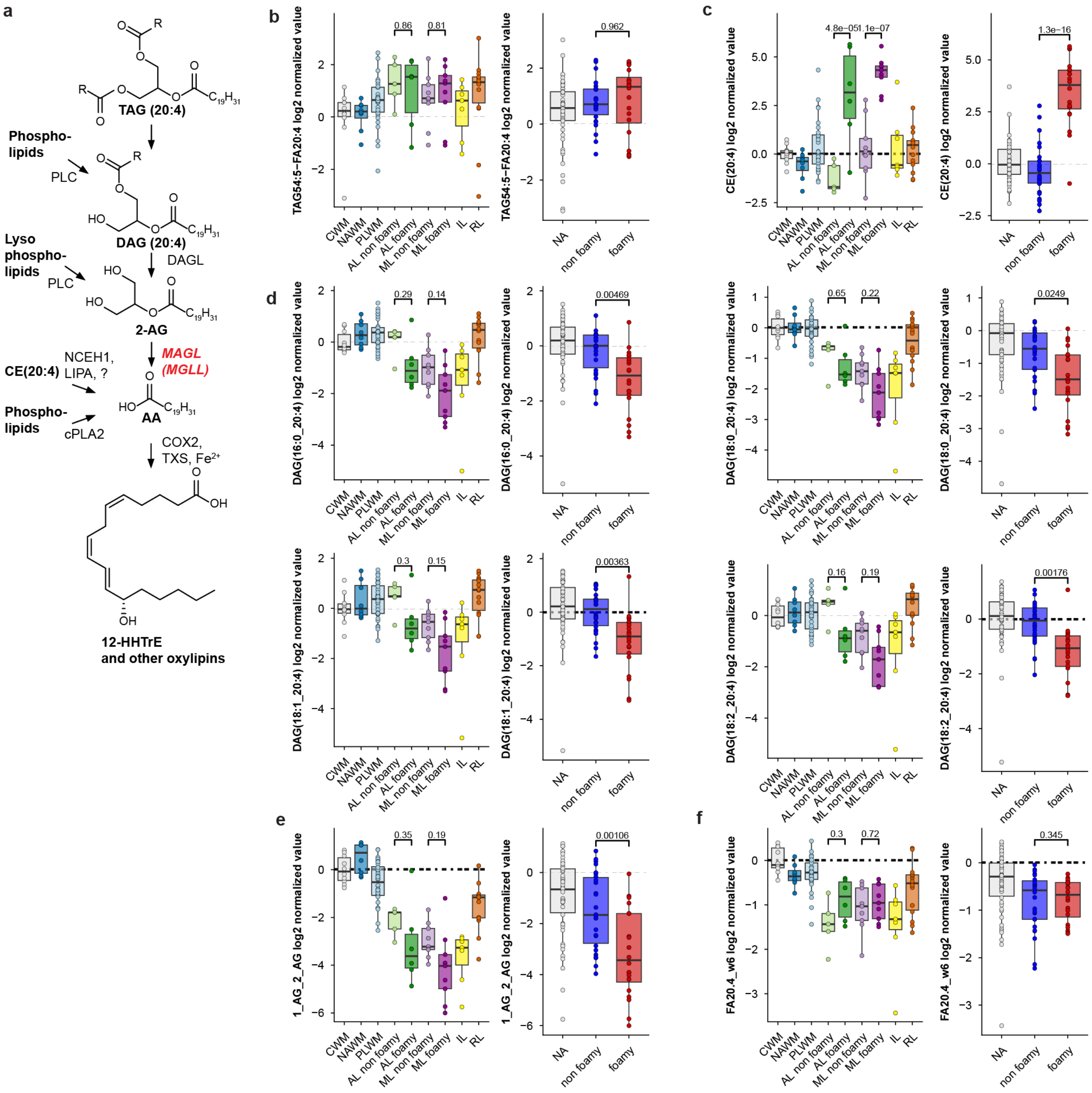

Supplementary Fig. 13 | Metabolic pathways for the production of free arachidonic acid. a, the pathway starting from AA-containing TAG, which is hydrolysed to DAG, MAG and subsequently AA. MAGL plays a rate-limiting role in the supply of AA for oxylipin production by controlling the conversion of 2-AG to AA. **b-f,** boxplots indicating the levels of lipids indicated in **a**. TAG levels are increased in all lesion types, but AA-containing DAG and 2-AG are decreased in lesions with foamy microglia compared to non-foamy, while oxylipin production was upregulated suggesting an increased flux to arachidonic acid. For panels **b-f**, data is expressed as individual values (circles), the median as the centre, boxes representing the first and third quartile and whiskers extending to datapoints within the 1.5xinter-quartile range. Statistics are from limma (two-sided) with BH-correction for multiple testing.

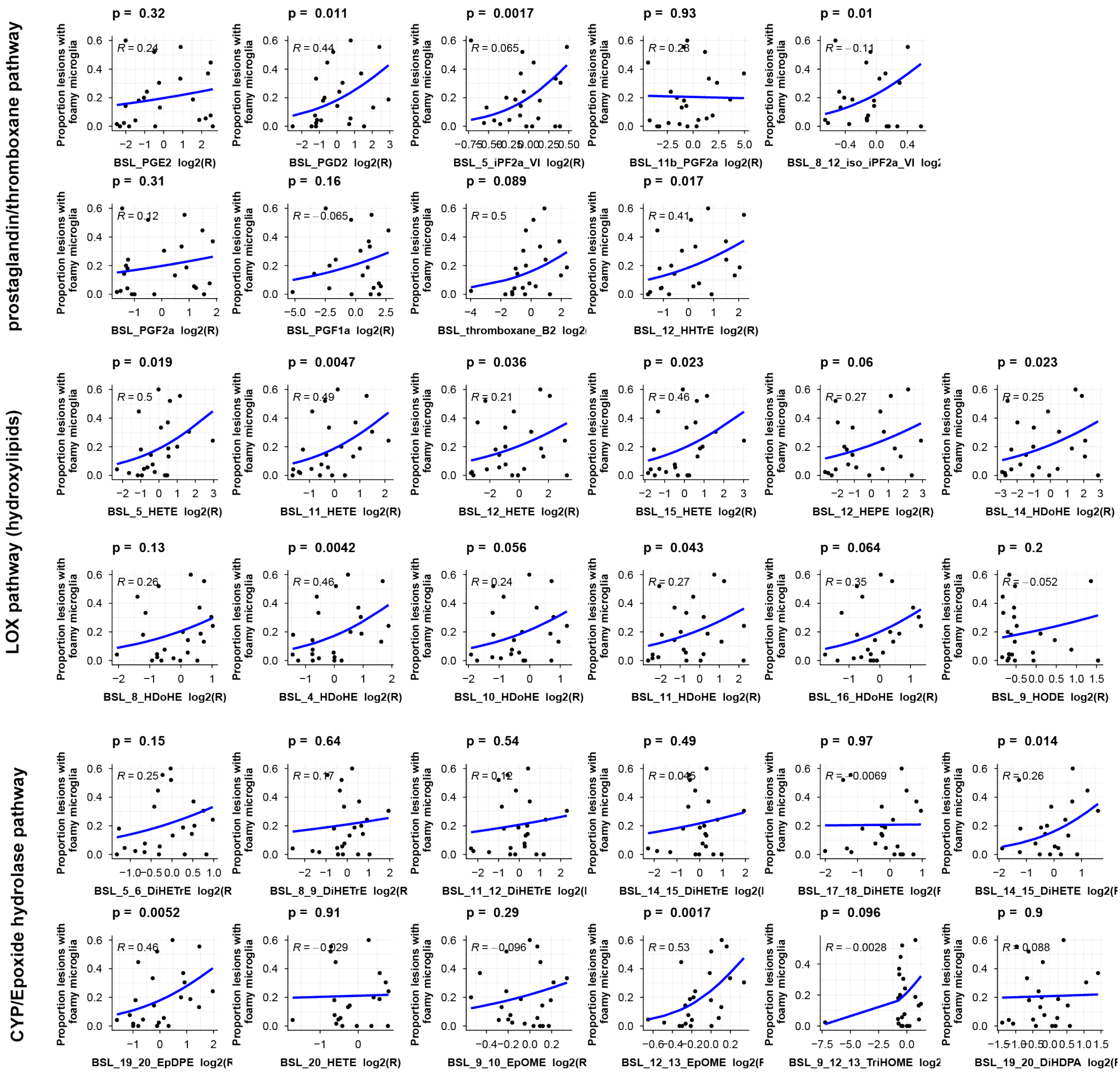

Supplementary Fig. 14 | A generalized linear model showing the association between CSF oxylipins to the proportion of foamy lesions, p-values are from a likelihood-ratio test. P-values were not corrected for multiple testing. Spearman correlations were performed to assess the variance explained by the association expressed as the spearman correlation coefficient *R*.

a Extended Data Fig. 10i | split channels

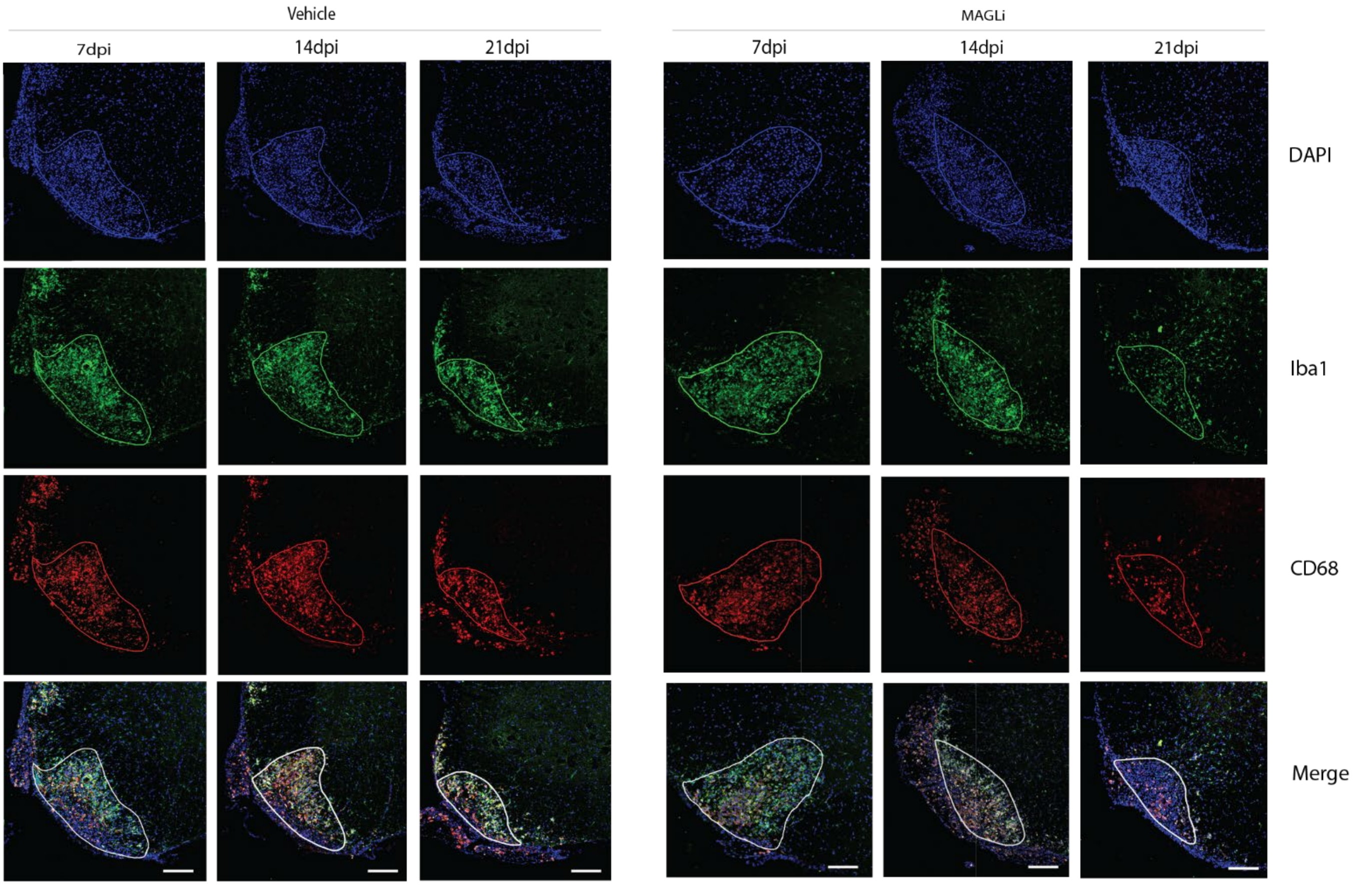

b Extended Data Fig. 10k | split channels

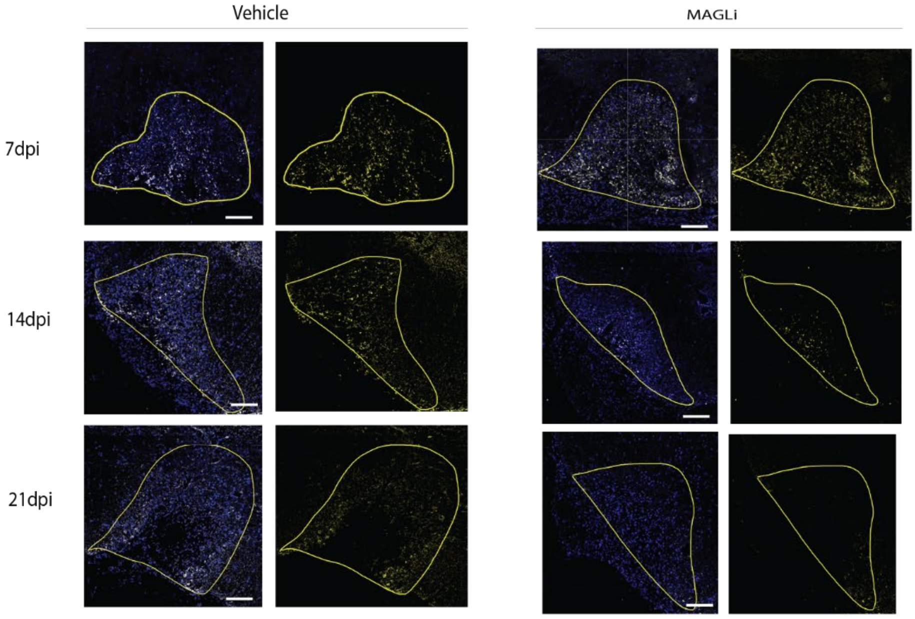

Blue: DAPI ; Yellow: dMBP

Supplementary Fig. 15 | split channel images for Extended Data Fig. 10i (a) and 10k (b). For detailed information see the caption of Extended Data Fig. 10.

Supplementary Methods

Lipidomics LC/MS-MS platforms

Signaling lipids platform (oxylipins, endocannabinoids)

The signaling lipids platform covers 196 metabolites, including various isoprostane classes together with their respective prostaglandin isomers from different poly unsaturated fatty acids (PUFA), including n-6 and n-3 PUFAs such as dihomo-γ-linoleic acid (DGLA) and arachidonic acid (n-6) and eicosapentaenoic acid (EPA) and docosahexaenoic acid (DHA) (n-3). Endocannabinoids, endocannabinoid analogues and bile acids are also included in this platform. Reference standards were used for each analyte for peak identification, 47 deuterated internal standards were used for the correction of variations from sample preparation and LC-MS runs[16].

A QTRAP 7500 (AB Sciex, Concord, ON, Canada) was coupled to an Exion LC AD (AB Sciex, Concord, ON, Canada). MS/MS experiments were done with a Turbo V source (AB Sciex, Concord, ON, Canada) operated with ESI probe. An Acquity UPLC BEH C18 column (Waters) was used to measure the samples. The three-pump LC system consisted of mobile phase A (MPA, H_2O with 0.1 % acetic acid), mobile phase B (MPB, 90 % CH_3CN, 10 % MeOH with 0.1 % acetic acid) and mobile phase C (MPC, IPA with 0.1 % acetic acid). The injection volume was 5 µL stacked with 10 µL of mobile phase A. The flow rate was 0.7 mL, min, and each run takes 16 min. The gradient started at 20 % MPB and 1% MPC. The MPB progressed from 20 % to 85 % between 0.75 min and 14 min while the MPC ascended from 1 % to 15 % between 11 min to 14 min after which conditions were kept for 0.3 min and then the column was re-equilibrated at initial conditions until 16 min. An electrospray ionization source (ESI) was used with parameters: interface temperature 600 °C, curtain gas 45 psi, CAD gas 9 psi, gas 1 and gas2 both 65 psi. The mass spectrometer operated in polarity switching mode and all analytes were monitored in dMRM mode. Data was acquired using Sciex OS Software V2.0.0.45330 (AB Sciex). Assigned MRM peaks from the acquired data were integrated using SCIEX OS (version 2.1.6) Software and signals were corrected using proper internal standards.
Blank effects (BE) for each analyte were checked by comparing proc blank samples to quality control (QC) samples. The precision and reproducibility of the analytical process were checked using the relative standard deviations (RSDs) of the QCs.
51 analytes complied with the high-confidence acceptance criteria of QC RSDs ≤ 15% and BE ≤ 40% in both QC pools (Normal white matter pool and lesion pool). 11 analytes had one or two of the QC RSDs within 15-30.6% or BE > 40% in one or both QC pools. These metabolites with high blank effect (>40%) were also reported as we know the blank effects are from the extractant, which is consistent and under control levels. The data is reported as relative response ratios (target area, ISTD area; unit free).

HILIC MS/MS based lipids platform (phospholipids, sphingolipids, BMPs)

This forward phase Lipid platform covers 1320 nonpolar lipids targets. The samples were measured using 3 separate acquisition methods. Acquisition method 1 measurement contains classes of phosphatidylcholine, phosphatidylinositol, phosphatidylserine, phosphatidylglycerol, and bis(monoacylglycerol)phosphates.

Acquisition method 2 measurement contains sphingomyelins, hexosylceramides, lactosylceramides and phosphatidylethanolamines. The acquisition method 3 measurement contains triglycerides. The identification of metabolites in this platform are based on the dimensions of specific MS/MS transitions and retention time (RT), Using HILIC columns, the lipids from the same class elute in a narrow RT window, while different lipid classes elute at different RTs. For each class, one or more deuterated internal standards were used to check the RTs and variations from sample preparation and LC-MS runs.

A QTRAP 6500+ (AB Sciex, Concord, ON, Canada) was coupled to an Exion LC AD (AB Sciex, Concord, ON, Canada). MS/MS experiments were done with a Turbo V source (AB Sciex, Concord, ON, Canada) operated with ESI probe. A Phenomenex Luna® amino column (100 mm × 2 mm, 3 μm) was used for separation. The mobile phase A was 1 mM ammonium acetate in chloroform: acetonitrile (1:9), while mobile phase B was 1 mM ammonium acetate in acetonitrile: water (1:1). The gradient started at 20 % MPB and 1% MPC. The MPB progressed from 20 % to 85 % between 0.75 min and 14 min while the MPC ascended from 1 % to 15 % between 11 min to 14 min after which conditions were kept for 0.3 min and then the column was re-equilibrated at initial conditions until 16 min. Two injections were made to accommodate all the MRM transitions of the targeted lipid features. The injection volume was 5 μL for the first acquisition run and 1 μL for the second acquisition run. The column temperature was kept at 35 °C. The injector needle was washed with isopropanol:water:dichloromethane (94:5:1, v, v, v) after each injection. Assigned MRM peaks from the acquired data were integrated using SCIEX OS (version 2.1.6) Software and signals were corrected using proper internal standards.

Blank effects for each analyte were checked by comparing proc blank samples to quality control (QC) samples. The precision and reproducibility of the analytical process were checked using the relative standard deviations (RSDs) of the QCs. With acquisition method 1, 305 analytes with QC RSDs <30% and blank effects < 40% in both QC pools were reported. 58 of the analytes had QC RSDs <15% in both QC pools. With acquisition method 2, 173 analytes with QC RSDs <30% and blank effects < 40% in both QC pools were reported. 70 of the analytes had QC RSDs <15% in both QC pools. With acquisition method 3, 5 TGs with QC RSDs <30% and blank effects < 40% in both QC pools were reported. All data is reported as relative response ratios.

Reverse phase (RP) lipids platform (ceramides, DAGs, CE)

The RP MS/MS-based lipids platform covers 186 lipids, including ceramides, diglycerides, and cholesterol esters. The identification of metabolites in this platform are based on the dimensions of specific MS/MS transitions and retention time (RT), Using reversed phase columns, the lipids from the same class elute at RTs that can fits into linear regression models involving carbon number and double bond number (RT mapping)[18]. For each class, one or more deuterated internal standards were used to check the RTs and variations from sample preparation and LC-MS runs.

A QTRAP 7500 (AB Sciex, Concord, ON, Canada) coupled to an Exion LC AD (AB Sciex, Concord, ON, Canada). MS/MS experiments were done with a Turbo V source (AB Sciex, Concord, ON, Canada) operated

with ESI probe. An Acquity UPLC BEH C8 column (Waters) was used to measure the samples. The gradient was the following: starting conditions 10% B and 10% C; increase of B from 10% to 40% between 1 min and 2 min; maintaining B at 40% and C at 10% between 2 min and 7 min; increase of C from 10% to 45% between 7 min and 8 min; maintaining B at 40% and C at 45% between 8 min and 10 min; returning to initial conditions at 10.5 min and re-equilibration for 1.5 min. The triple quadrupole mass spectrometer operated in polarity switching mode and all analytes were monitored in dMRM mode. Data was acquired using Sciex OS Software V2.0.0.45330 (AB Sciex).

Assigned MRM peaks from the acquired data were integrated using SCIEX OS (version 2.1.6) Software and signals were corrected using proper internal standards.

Blank effects for each analyte were checked by comparing proc blank samples to quality control (QC) samples. The precision and reproducibility of the analytical process were checked using the relative standard deviations (RSDs) of the QCs.
In total 89 analytes with QC RSDs <30% in both QC pools and blank effects < 40% were reported. 43 of these analytes had QC RSDs <15% in both QC pools. The data is reported as relative response ratios.

Endocannabinoid synthesis lipid platform (NAPE, lysoNAPE, GPNAE, free fatty acids)

The endocannabinoid synthesis platform covers *N*-acyl-phosphatidylethanolamines (NAPEs), 2-lyso-*N*-acyl-phosphatidylethanolamines (lyso-NAPEs), glycerol-phospho-acylethanolamines (GP-NAEs), free fatty acids (FFAs), which profiles the metabolic pathways of *N*-acyl ethanolamines (NAEs).

A QTRAP 6500+ (AB Sciex, Concord, ON, Canada) coupled to an Exion LC AD (AB Sciex, Concord, ON, Canada). MS/MS experiments were done with a Turbo V source (AB Sciex, Concord, ON, Canada) operated with ESI probe. The separation was performed in a BEH C8 column (50 mm × 2.1 mm, 1.7 μm) from Waters Technologies (Mildford, MA, USA) maintained at 40°C, with the flow rate at 0.4 mL, min. The mobile phase was consisted of 2 mM $HCOONH_4$, 10 mM formic acid in water (A), ACN (B), IPA (C). The gradient was the following: starting conditions 20% B and 20% C; increase of B from 20% to 40% between 1 min and 2 min; maintaining B at 40% and C at 20% between 2 min and 7 min; increase of C from 20% to 50% between 7 min and 8 min; maintaining B at 40% and C at 50% between 8 min and 10 min; returning to initial conditions at 10.5 min and re-equilibration for 1.5 min. The triple quadrupole mass spectrometer operated in polarity switching mode and all analytes were monitored in dMRM mode. Data was acquired using Sciex OS Software V2.0.0.45330 (AB Sciex).

Assigned MRM peaks from the acquired data were integrated using SCIEX OS (version 2.1.6) Software and signals were corrected using proper internal standards.

Blank effects for each analyte were checked by comparing proc blank samples to quality control (QC) samples. The precision and reproducibility of the analytical process were checked using the relative standard deviations (RSDs) of the QCs.

In total 68 analytes with QC RSDs <30% in both QC pools and blank effects < 40% were reported. 31 of these analytes had QC RSDs <15% in both QC pools. The data is reported as relative response ratios.

Cholesterol synthesis and oxysterols platform

The cholesterol synthesis and oxysterols platform covers precursors of cholesterol from the Bloch and Kandutsch-Russell pathways, including lathosterol and desmosterol. Oxysterols, which are cholesterol metabolites that can be produced through enzymatic or radical oxidation, are also covered. Reference standards or corresponding deuterated internal standards were used for each analyte for peak identification, 5 deuterated internal standards were used for the correction of variations from sample preparation and LC-MS runs.

A QTRAP 7500 (AB Sciex, Concord, ON, Canada) coupled to an Exion LC AD (AB Sciex, Concord, ON, Canada). MS/MS experiments were done with a Turbo V source (AB Sciex, Concord, ON, Canada) operated with ESI probe. The separation was performed in a BEH C18 column (50 mm × 2.1 mm, 1.7 µm) from Waters Technologies (Mildford, MA, USA) maintained at 40 °C, with the flow rate at 0.6 mL, min. The mobile phase was consisted of 0.1% acetic acid in water (A), ACN (B), MeOH (C). The gradient was the following: starting conditions 27% B and 27% C; increase of B & C from 27% to 30% between 1 min and 2 min; increase of B & C from 30% to 48% between 1 min and 2 min; maintaining B & C at 48% between 6.5 min and 7 min; increase of B & C from 48% to 50% between 7 min and 10 min; maintaining B & C at 50% between 10 min and 11 min; returning to initial conditions at 11 min and re-equilibration for 1 min. The triple quadrupole mass spectrometer operated in polarity switching mode and all analytes were monitored in dMRM mode. Data was acquired using Sciex OS Software V2.0.0.45330 (AB Sciex).

Assigned MRM peaks from the acquired data were integrated using SCIEX OS (version 2.1.6) Software and signals were corrected using proper internal standards.

Blank effects for each analyte were checked by comparing proc blank samples to quality control (QC) samples. The precision and reproducibility of the analytical process were checked using the relative standard deviations (RSDs) of the QCs. In total 5 analytes with QC RSDs <30% in both QC pools and blank effects < 40% were reported. Desmosterol and 7-dehydrocholesterol had high RSDs in lesion QC pools, but acceptable RSDs in white matter QC pools and were kept. The data is reported as relative response ratios.

Oxidized phospholipids platform

The oxidized phospholipids (OxPLs) platform covers a class of lipid molecules formed when phospholipids undergo oxidative modification. Reference standards of oxidized phosphatidylcholines (oxPCs) were used for peak identification. More OxPCs and OxPEs were identified based on MS/MS transitions and HRMS/MS at positive mode. Deuterated internal standards of PCs, LPCs and eCBs were used for the correction of variations from sample preparation and LC-MS runs.

A QTRAP 7500 (AB Sciex, Concord, ON, Canada) coupled to an Exion LC AD (AB Sciex, Concord, ON, Canada). MS/MS experiments were done with a Turbo V source (AB Sciex, Concord, ON, Canada) operated with ESI probe. The separation was performed in a BEH C8 column (50 mm × 2.1 mm, 1.7 μm) from Waters Technologies (Mildford, MA, USA) maintained at 40°C, with the flow rate at 0.4 mL, min. The mobile phase was consisted of 2 mM ammonium acetate, ACN (B), IPA (C). The gradient was the following: starting conditions 10% B and 10% C; increase of B from 10% to 45% between 1 min and 2.5 min; maintaining B at 45% and C at 10% between 2.5 min and 4 min; increase of C from 10% to 48% between 4 min and 7.5 min; maintaining B at 45% and C at 48% between 7.5 min and 10 min; returning to initial conditions at 10.5 min and re-equilibration for 1.5 min. The triple quadrupole mass spectrometer operated in polarity switching mode and all analytes were monitored in dMRM mode. Data was acquired using Sciex OS Software V2.0.0.45330 (AB Sciex).

Assigned MRM peaks from the acquired data were integrated using SCIEX OS (version 2.1.6) Software and signals were corrected using proper internal standards.

Blank effects for each analyte were checked by comparing proc blank samples to quality control (QC) samples. The precision and reproducibility of the analytical process were checked using the relative standard deviations (RSDs) of the QCs.

In total 26 analytes with QC RSDs <30% in both QC pools and blank effects < 40% were reported. The data is reported as relative response ratios.

To estimate absolute concentrations, a calibration line containing OxPCs and PC (16:0, 20:4) was prepared separately from the study samples. A response (area ratio) – concentration relationship was established with the calibration line, and the concentrations of OxPCs and PC (16:0, 20:4) were calculated, but only for those lipids for which a standard was available.

RNA sequencing data processing

The raw count data were loaded in R (v4.4.1) and a DGEList object was created using EdgeR. Samples with library sizes < 2 million were excluded. This threshold was determined by inspecting count distributions across the samples. These criteria led to the exclusion of four samples, leaving 105 samples for subsequent analysis. Count distributions were found to be satisfactory and mostly uniform after the exclusion of the 4 samples with low library sizes.

Criteria for gene filtering (n=61,541) were having > 2 counts per million (CPM) on average in at least one sample type using the 'cpmbygroup' function in EdgeR. Additionally, we excluded immunoglobulin variable regions and only kept constant regions. This left 16,652 genes for further analysis. CPM values were calculated using log2(counts+0.5 per million) with normalization using the trimmed-mean method (TMM) in EdgeR. These data were inspected for correlation with technical and biological covariates using principal component analysis (PCA). CPM values used in PCA analysis, MOFA and WGCNA and all heatmap visualizations were corrected for covariates using the 'RemoveBatchEffect' function in *Limma*, with the design *y = ~0 + Lesion type + RQN + sex*, Lesion type categories (9 groups) being the design, and RQN and sex being the covariates to be regressed out.

Weighted gene coexpression network analysis (WGCNA)

A WGCNA-based co-expression network was constructed using the WGCNA package (v1.72-5), and its dependencies fastcluster (v1.2.6), dynamicTreeCut (v1.63-1), preprocessCore (v1.66.0 and impute (v1.78.0). The transcriptomics input was corrected for *RQN* and sex as described above. Because we were interested in the differences between lesions and not necessarily between lesions and control or normal appearing white matter, we excluded all non-lesion samples. Indeed, a constructed network using all samples dissected the data into fewer modules, with two main modules that represent genes upregulated in all lesions, and genes downregulated in all lesions (data not shown). Genes for WGCNA input were selected for high variance, including those genes with a standard deviation within lesion samples higher than the mean standard deviation, yielding an input dataset of 52 lesion samples and 6,547 genes.

To determine the power threshold β, the function PickSoftThreshold was used, where we tested powers between 1 and 20, and then assessed scale-free topology. At β = 13, the threshold (R^2 between log(freq) and log(connectivity) = 0.85) for approximate scale-free topology was reached. Next, WGCNA Modules were generated using the BlockwiseModules function, with corType set to "bicor", TOMtype set to "signed", networkType set to "signed", a power β of 13, mergeCutHeight set to 0.2 and a minimum module size of 30. A signed network was chosen to preserve the directionality of the generated modules, improving interpretability of GO terms associated with the module. The biweight midcorrelation ("bicor") was chosen over the Pearson correlation to make the method less sensitive to outliers and more robust, which generates more meaningful modules, as the authors of WGCNA recommend[1].

This dissected the dataset into 16 modules ranging in size from 56 to 968 genes. 435 genes were not assigned to any module (Module 0). Module eigengenes were calculated using the function 'moduleEigengenes'. The module eigengenes consistently explained high percentage of variation within the

module (except for Module 0), ranging from 38.5% to 59.2% indicating that these eigengenes represent the modules well.
Module eigengenes were correlated to traits, in this case the lesion types, using the pearson correlation, and with subsequent calculation of p-values by the function 'corPvalueStudent'. Acquired p-values were corrected for multiple testing of the 17 modules using the Benjamini-Hochberg method with an FDR set at 10%. Hub genes were identified based on module membership (kME > 0.7) and gene significance (GS > 0.5, following standard WGCNA procedures described by Langfelder & Horvath[1].

GO terms associated with module-genes were found using clusterProfiler (v4.6.1) and the org.Hs.eg.db_3.19.1. We used the enrichGO function with all WGCNA input genes as background (universe), the module genes as input, ontology set to "ALL", minGSSize set to 10 and MaxGSSize set to 500, and a p-value cutoff of 0.05. KEGG pathways were found using the enrichKEGG function, with the same inputs. Heatmaps showing expression data for the modules were generated using the ComplexHeatmap package[2] (v2.20.0) with the module genes log2 Z-scored counts per million as input.

Cell-type enrichment was performed using the function *UserListEnrichment* with a reference list of biomarkers of various cell types from CellMarker 2.0, a database containing an up-to-date manually curated collection of markers of various cell types in different human tissues[3]. All the markers from brain and blood were extracted from the dataset and pre-processed into an input reference list including 915 categories (Cell types) and 6517 markers (genes). All modules were tested for cell type enrichment, except module 0. P-values were corrected with Bonferroni multiple testing correction, which is default in the WGCNA workflow and *UserListEnrichment* function.

snRNAseq dataset processing & deconvolution

Single-nucleus RNA sequencing data was obtained from Macnair et al.[4] White matter samples were selected and counts from the same broad cell type (7 cell types) were combined into one pseudobulk per sample. Then, a DGEList object was created using EdgeR and the samples were normalized using the 'TMMwsp' method in EdgeR. This was chosen over normal TMM, because the snRNAseq data contains many zero's which affects normalization. Samples were then filtered for minimum library size of 20,000. Genes were filtered for highly expressed genes by requiring a mean CPM in at least one cell type of 10 using the 'cpmbygroup' function. This gave a dataset of 533 pseudobulk profiles across 7 cell types (astrocytes, oligodendrocytes, OPCs, microglia, vasculature, B_cells and T_cells) with 15,084 genes.

Marker genes were calculated using limma, where we tested each celltype against the combined other cell types. Differentially expressed genes were determined using voom and eBayes functions as described above. Further criteria for marker genes were a log2FC of at least 4 against the average of the other cell types, an adjusted p-value of smaller than 0.00001, and that the marker gene was unique. We then restricted the list to the top 100 best marker genes (ranked by logFC*-log10(adjusted p-value)) for deconvolution. For B cells, we excluded immunoglobulin genes from the analysis, because their expression per B-cell is extremely variable and can be extremely high, leading to overestimation of the number of B-cells. Given

these criteria, not every cell type had 100 marker genes. The number of marker genes were between 75 and 100. Plotting these marker genes against the pseudobulk profiles consistently gave very clean profiles (Extended Data Fig. 7a).

Deconvolution was subsequently performed using the R package 'dtangle' (version 2.0.9). The input data for dtangle were: 1) The log2(cpm) bulk data normalized by edgeR without any corrections for covariates. 2) The log2(cpm) pseudobulk data from snRNAseq normalized by edgeR as described above, and 3) The lists of marker genes
Further settings were a gamma of 0.9433902 as recommended by the authors of dtangle for RNAseq data, and the option "summary_fn" was set to "mean".

To validate the method and the marker genes, the pseudobulk dataset was randomly split into two parts (80% training, 20% test). We then used the dTangle algorithm with the same settings, and the same marker genes to predict the cell type composition of the pure pseudobulk profiles in the test dataset. This consistently gave very high percentages of the correct cell type around ~90% (Extended Data Fig. 7b).

Evaluation of microglia subclusters

Microglial states were analyzed from the single-nucleus RNA sequencing data from Macnair et al 2023[4].White matter samples were selected and counts from microglia subclusters (7 microglia clusters: Micro_A, Micro_B, Micro_C, Micro_D, Micro_E, PVM, Micro_Prolif) were combined into pseudobulk per sample. Then, a DGEList object was created using EdgeR and the samples were normalized using the 'TMMwsp' method in EdgeR. Pseudobulk profiles were then filtered for minimum library size of 10,000. Genes were filtered for highly expressed genes by requiring a mean CPM in at least one subcluster of 10 using the 'cpmbygroup' function. This gave a dataset of 442 pseudobulk profiles across 7 subclusters with 11,575 genes.

Marker genes for each microglia subcluster were calculated using using limma, where we tested each microglia subcluster against the combined others. Differentially expressed genes were determined using voom and eBayes functions as described above. Further criteria for marker genes were a positive FC, a BH-adjusted p-value of smaller than 0.05, and that the gene was expressed mainly in microglia (more than 50% of all counts in the complete snRNAseq dataset came from microglia). Given these criteria, we identified between 24 and 328 marker genes for each subcluster. The marker genes were ranked based on logFC, and the top 30 markers genes were selected to compose a gene-signature. To then estimate the relative presence of each microglial subcluster in the bulk RNAseq dataset, we used gene-set variation analysis (GSVA)[5] with each marker gene list as a gene-set. To control for microglial numbers in the bulk RNAseq data, we took the estimated microglial proportion as described above, and we used Limma's 'RemoveBatchEffect' function to correct for the log2-transformed microglia abundance, as well as sex and RQN. This corrected dataset was used in GSVA[5]. Further settings for GSVA were kcdf set to 'Gaussian' and maxDiff set to 'TRUE'. This resulted in a GSVA score per microglial state between -1 and 1 for all samples. We then tested all microglia subcluster signatures between lesion types, and between foamy and non-foamy samples using the Wilcoxon-rank sum test with Benjamini-Hochberg correction for multiple testing.

Deconvolution of the microglia states was also tried using dtangle as described for the broad cell types. However, this did not yield good results because there is too much collinearity between the distinct microglia subclusters. Validation of this method using a part of the snRNAseq pseudobulk did not purely enrich the right cell type, but also enriched other microglial subclusters.

Gene set enrichment analysis (GSEA)

GSEA was performed using log2-fold changes as input with the ClusterProfiler package (v4.6.2), function 'gseGO'. Ontology was set to "ALL", the database was Org.Hs.eg.db (v3.16.0), maxGSSize=100, minGSsize=10, eps=1e-300 and pvaluecutoff=0.05. MOFA factor loadings from proteomics and RNAseq were also investigated using GSEA, using the same settings, with the relative factor loadings (from -1 to 1) as input. Lipid class enrichment was performed using fgsea (v1.24.0) using custom lipid class lists (31 classes).

References

1. Langfelder, P. & Horvath, S. WGCNA: an R package for weighted correlation network analysis. *BMC Bioinformatics* **9**, 559 (2008).
2. Gu, Z., Eils, R. & Schlesner, M. Complex heatmaps reveal patterns and correlations in multidimensional genomic data. *Bioinformatics* **32**, 2847–2849 (2016).
3. Hu, C. *et al.* CellMarker 2.0: an updated database of manually curated cell markers in human/mouse and web tools based on scRNA-seq data. *Nucleic Acids Res.* **51**, D870–D876 (2023).
4. Macnair, W. *et al.* snRNA-seq stratifies multiple sclerosis patients into distinct white matter glial responses. *Neuron* **113**, 1–15 (2024).
5. Hänzelmann, S., Castelo, R. & Guinney, J. GSVA: gene set variation analysis for microarray and RNA-Seq data. *BMC Bioinformatics* **14**, 7 (2013).
