# Supplementary material for: Foamy microglia link oxylipins to disease progression in multiple sclerosis
Source: Nat Neurosci. 2026 May 21;29(7):1585–98. doi: 10.1038/s41593-026-02302-3 (PMC13337515; doi:10.1038/s41593-026-02302-3)
Supplement: Supplementary file 2 — Reporting Summary [file 41593_2026_2302_MOESM2_ESM.pdf]

Corresponding author(s): Prof. Mario van der Stelt
Prof. Inge Huitinga

Last updated by author(s): Apr 2, 2026

Reporting Summary

Nature Portfolio wishes to improve the reproducibility of the work that we publish. This form provides structure for consistency and transparency in reporting. For further information on Nature Portfolio policies, see our Editorial Policies and the Editorial Policy Checklist.

Statistics

For all statistical analyses, confirm that the following items are present in the figure legend, table legend, main text, or Methods section.

n/a	Confirmed	
☐	☒	The exact sample size (*n*) for each experimental group/condition, given as a discrete number and unit of measurement
☐	☒	A statement on whether measurements were taken from distinct samples or whether the same sample was measured repeatedly
☐	☒	The statistical test(s) used AND whether they are one- or two-sided<br>*Only common tests should be described solely by name; describe more complex techniques in the Methods section.*
☐	☒	A description of all covariates tested
☐	☒	A description of any assumptions or corrections, such as tests of normality and adjustment for multiple comparisons
☐	☒	A full description of the statistical parameters including central tendency (e.g. means) or other basic estimates (e.g. regression coefficient) AND variation (e.g. standard deviation) or associated estimates of uncertainty (e.g. confidence intervals)
☐	☒	For null hypothesis testing, the test statistic (e.g. *F*, *t*, *r*) with confidence intervals, effect sizes, degrees of freedom and *P* value noted<br>*Give P values as exact values whenever suitable.*
☒	☐	For Bayesian analysis, information on the choice of priors and Markov chain Monte Carlo settings
☒	☐	For hierarchical and complex designs, identification of the appropriate level for tests and full reporting of outcomes
☐	☒	Estimates of effect sizes (e.g. Cohen's *d*, Pearson's *r*), indicating how they were calculated

Our web collection on statistics for biologists contains articles on many of the points above.

Software and code

Policy information about availability of computer code

Data collection	MaxQuant v2.0.1.0, DIA-NN v1.8.1, HISAT v2.2.0, HTSeq v2.0.2, SCIEX OS v2.1.6, QuPath v0.5.0, HALO (Indica Labs)
Data analysis	All data anlysis was performed in R v4.2.2 or R v4.4.1using Rstudio environment in Graphpad Prism 10.0. Key packages used in R were tidyverse (v2.0.0), magrittr (v2.0.3), rstatix (v0.7.2), ggpubr (v0.6.0), ggplot2 (v3.5.1), ComplexHeatmap (v2.2.0), cowplot (v1.1.3) and ggrepel (v0.9.5), WGCNA v1.72-5, clusterprofiler v.4.6.1, org.hs.eg.db v3.16.0, EdgeR v4.2.1, Limma 3.60.4, MOFA2 v1.8.0, uwot v0.1.16, slingshot v2.6.0, GSVA v1.52.3, Mclust v6.1, geepack v1.3.10, fastcluster v1.2.6, dynamicTreeCut (v1.63-1), preprocessCore (v1.66.0), impute (v1.78.0), fgsea v1.24.0, dtangle v2.0.9,

For manuscripts utilizing custom algorithms or software that are central to the research but not yet described in published literature, software must be made available to editors and reviewers. We strongly encourage code deposition in a community repository (e.g. GitHub). See the Nature Portfolio guidelines for submitting code & software for further information.

Data

Policy information about availability of data

All manuscripts must include a data availability statement. This statement should provide the following information, where applicable:

- Accession codes, unique identifiers, or web links for publicly available datasets
- A description of any restrictions on data availability
- For clinical datasets or third party data, please ensure that the statement adheres to our policy

RNA sequencing data is available from the gene expression omnibus (GEO) database under accession number GSE279972. Mass spectrometry data is available through the ProteomeXchange Consortium via PRIDE upon publication (Proteomics: PXD056856 and ABPP: PXD056899).
All processed data are also available via Zenodo: DOI: 10.5281/zenodo.17735822

Research involving human participants, their data, or biological material

Policy information about studies with human participants or human data. See also policy information about sex, gender (identity/presentation), and sexual orientation and race, ethnicity and racism.

Reporting on sex and gender	In our study we have selected samples from humans with both sexes and we matched the groups closely that the male/female ratio is approximately 50/50. This was done to reach conclusion that would be appliccable for both sexes and not just one group. Gender was considered in this study and is not known to the researchers.
Reporting on race, ethnicity, or other socially relevant groupings	Race or ethnicity was not considered during human subject selection and is not known to the researchers.
Population characteristics	Human brain samples from 38 donors (28 Secondary Progressive MS and 10 matched non-demented controls) were obtained from the Netherlands Brain Bank). Selection criteria for donors were a post-mortem delay (PMD, time-interval between the demise of the donor and freezing of the tissue) below 12 hours, a pH of the CSF higher than 5.5, and a clinician-confirmed diagnosis of secondary progressive MS for MS patients and absence of any neurological disease in controls, nor neurological disease-indicating pathology found in their brains as examined by a neuropathologist. The PMD ranged between 6:30 hours to 11:30, with a median of 8:45 hours. The pH of the CSF ranged from 5.8 to 6.8, with a median of 6.4. Age ranged from 39 to 95 with a median of 62 years.
Recruitment	Donors applied for the Netherlands Brain bank Donor programme
Ethics oversight	All procedures were approved by the medical ethics committee of the VU medical center (Amsterdam, The Netherlands)

Note that full information on the approval of the study protocol must also be provided in the manuscript.

Field-specific reporting

Please select the one below that is the best fit for your research. If you are not sure, read the appropriate sections before making your selection.

☒ Life sciences ☐ Behavioural & social sciences ☐ Ecological, evolutionary & environmental sciences

For a reference copy of the document with all sections, see nature.com/documents/nr-reporting-summary-flat.pdf

Life sciences study design

All studies must disclose on these points even when the disclosure is negative.

Sample size	No power calculations were performed to determine sample size. Based on previous studies we aimed for 7-8 replicates for each lesion type. However, during isolation of the lesions these numbers changed because some lesions were too small to isolate, while sometimes new lesions appeared. The number of replicates was based on our previous studies (Hendrickx et al. 10.3389/fimmu.2017.01810, Chen et al 10.1093/brain/awae414, macnair et al 10.1016/j.neuron.2024.11.016, Melief et al. 10.1186/s40478-019-0705-7)
Data exclusions	4 samples from RNAsequencing analysis were excluded based on a too low library size for proper analysis. 3 samples for proteomics were excluded based on too many missing values (similar to a low library size). No samples have excluded based on results, also not if they were judged to be an outlier.
Replication	Due to scarcity of tissue and limitations on number of tissue blocks approved by the NBB no validation cohort was set up. Data was validated with immunohistochemistry. Because of the unique nature of lesions and MS patients the work herein cannot be directly replicated.
Randomization	Samples were always randomized upon arrival and before measurement using numbers generated by random.org
Blinding	No formal blinding was applied because our measurements are quantitative and not sensitive to human subjective interpretation. In addition, all samples were processed and measured while being labeled with a sample number, not the experimental category itself, so the chances of bias are small.

Reporting for specific materials, systems and methods

We require information from authors about some types of materials, experimental systems and methods used in many studies. Here, indicate whether each material, system or method listed is relevant to your study. If you are not sure if a list item applies to your research, read the appropriate section before selecting a response.

Materials & experimental systems

n/a	Involved in the study
☐	☒ Antibodies
☒	☐ Eukaryotic cell lines
☒	☐ Palaeontology and archaeology
☐	☒ Animals and other organisms
☒	☐ Clinical data
☒	☐ Dual use research of concern
☒	☐ Plants

Methods

n/a	Involved in the study
☒	☐ ChIP-seq
☒	☐ Flow cytometry
☒	☐ MRI-based neuroimaging

Antibodies

Antibodies used	PLP1 (plpc1, MCA839G, BioRad,1:1000), HLA-DR/DP/DQ (CR3/43, M0775, DAKO,1:1000), CD79a (JCB117, M705001-2, DAKO, 1:2000), CD3e (A0452, DAKO, 1:100), GFAP (D1F4Q, 12389, Cell signaling, 1:1000), GPNMB (E4D7P, 38313, Cell signaling, 1:500), TBXAS1 (160715, Cayman Chemical, 1:200), PLIN2 (GP40, PROGEN2, 1:200), LAMP1 (Ab24170, Abcam, 1:200).
Validation	Antibodies against PLP1, HLA and CD3e have been extensively validated by previous work of our group (Luchetti et al. Acta Neuropathologica 2018, Hendrickx et al. Journal of Neuroimmunology 2017, Fransen et al. Brain 2020).<br>PLIN2 antibodies have been validated previously by Menezes Maya-Monteiro et al. Neuroendocrinology 2021;111:263–272.<br>LAMP1 antibodies was previously validated by us (Van den Bosch et al. Nature Communications (2024) 15:1667<br>CD79a was previously validated by us (Klotz et al. Nature Medicine 31, 2016–2026 (2025))<br>Antibodies against GPNMB (https://www.cellsignal.com/products/primary-antibodies/gpnmb-e4d7p-rabbit-monoclonal-antibody/38313), TBXAS1 (https://www.caymanchem.com/product/160715/thromboxane-synthase-polyclonal-antibody?srsltid=AfmBOoq-rBR-aUTnQqdkhEvrDFnBcw-Vsr3XJZsDHYBFBxX0NEdfZTJg) and GFAP (https://www.cellsignal.com/products/primary-antibodies/gfap-d1f4q-rabbit-monoclonal-antibody/12389) have validated by the manufacturer in human samples.

Animals and other research organisms

Policy information about studies involving animals; ARRIVE guidelines recommended for reporting animal research, and Sex and Gender in Research

Laboratory animals	C57BL/6JRj mice obtained from Janvier Labs (France).
Wild animals	NA
Reporting on sex	We have used both male and female mice.<br>Sex was not considered in the design of the study. To the best of our knowledge, there are no reported effects of sex in the formation of demyelinating lesions in the spinal cord using lysophosphatidylcholine (LPC).
Field-collected samples	NA
Ethics oversight	All animal experiments were conducted in accordance with the Swiss Federal Act on Animal Protection and were approved by the Cantonal Veterinary Office Basel-Stadt, Switzerland (license No. 3095).

Note that full information on the approval of the study protocol must also be provided in the manuscript.

Plants

Seed stocks	*Report on the source of all seed stocks or other plant material used. If applicable, state the seed stock centre and catalogue number. If plant specimens were collected from the field, describe the collection location, date and sampling procedures.*
Novel plant genotypes	*Describe the methods by which all novel plant genotypes were produced. This includes those generated by transgenic approaches, gene editing, chemical/radiation-based mutagenesis and hybridization. For transgenic lines, describe the transformation method, the number of independent lines analyzed and the generation upon which experiments were performed. For gene-edited lines, describe the editor used, the endogenous sequence targeted for editing, the targeting guide RNA sequence (if applicable) and how the editor was applied.*
Authentication	*Describe any authentication procedures for each seed stock used or novel genotype generated. Describe any experiments used to assess the effect of a mutation and, where applicable, how potential secondary effects (e.g. second site T-DNA insertions, mosiacism, off-target gene editing) were examined.*
